# Chelation-Driven Dissolution and Single-Crystal Growth of Hybrid Metal Organochalcogenide Semiconductors by Polydentate Amines

**DOI:** 10.1021/jacs.5c10260

**Published:** 2025-10-04

**Authors:** Rattapon Khamlue, Petcharaphorn Chatsiri, Tomoaki Sakurada, Jesadaporn Chotimook, Pimpan Leangtanom, Pongkamon Prayongkul, Thassanant Atithep, Jintara Padchasri, Pinit Kidkhunthod, Martin Vacha, Pichaya Pattanasattayavong, Watcharaphol Paritmongkol

**Affiliations:** † Department of Materials Science and Engineering, School of Molecular Science and Engineering, Vidyasirimedhi Institute of Science and Technology (VISTEC), Rayong 21210, Thailand; ‡ Department of Chemical and Biomolecular Engineering, School of Energy Science and Engineering, Vidyasirimedhi Institute of Science and Engineering (VISTEC), Rayong 21210, Thailand; § Department of Materials Science and Engineering, Institute of Science Tokyo, Ookayama 2-12-1, Meguro-ku, Tokyo 152-8552, Japan; ∥ Yokohama Technical Center, AGC Inc., Yokohama, Kanagawa 230-0045, Japan; ⊥ Frontier Research Center (FRC), Vidyasirimedhi Institute of Science and Technology (VISTEC), Rayong, 21210, Thailand; ¶ 530102 Synchrotron Light Research Institute (Public Organization), 111 University Avenue, Muang, Nakhon Ratchasima 30000, Thailand; ∇ Department of Chemical Engineering, Faculty of Engineering, Chulalongkorn University, Bangkok 10330, Thailand

## Abstract

The ability to grow single crystals is critical to modern society, particularly forming the foundation of the semiconductor industry that drives transformative innovations. While inorganic semiconductors are typically grown using melt-based methods, and organic semiconductors via solution processing, these approaches are often unsuitable for hybrid organic–inorganic semiconductors, which tend to exhibit low thermal stability and high solvent resistance. Here, we report a general solution-based strategy to overcome this challenge by exploiting polydentate amine solvents to dissolve and recrystallize previously intractable metal organochalcogenides (MOCs)—an emerging family of hybrid semiconductors, with potentials for robust luminescence, strong exciton binding energy, and electronic anisotropy. Using silver phenylselenide as a model system, we show that chelation-driven solubility in amines enables the growth of up to 1.02 cm × 0.54 cm single crystals with enhanced spectral purity, and that this strategy is general across MOC derivatives with diverse organic and chalcogen components. Recrystallization from these solvents yields high-quality single crystals of 15 MOCs, including eight previously unreported structures and three adopting noncentrosymmetric or polar space groups. Mechanistic studies reveal that dissolution proceeds via fragmentation into neutral, nanoscale polymeric units rather than ionic dissociation. These findings not only deepen the understanding of MOC solubility but also establish a broadly applicable platform for accessing single crystals of hybrid semiconductors. Together with the observation that several MOCs adopt 2D layered and noncentrosymmetric architectures, this work positions MOCs as a new family of next-generation, solution-processable semiconductors and opens avenues for crystallizing other insoluble hybrid materials.

## Graphical abstract

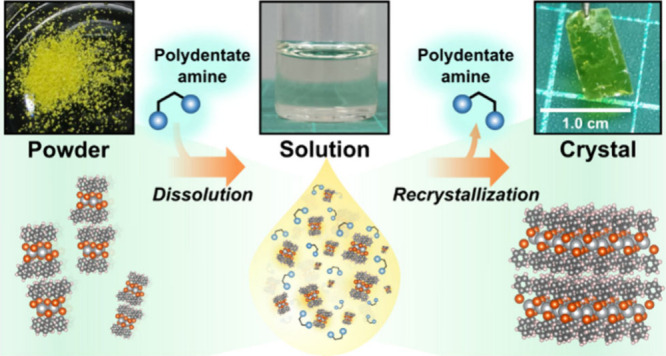

## 1 Introduction

Single crystals, characterized by their absence of grain boundaries and minimal defect densities compared to polycrystalline forms, are foundational to modern technologies.^1,2^ This is particularly the case in the field of semiconductors where the ability to grow silicon single crystals with exceptional electrical, optical, and thermal properties compared to other forms has driven numerous innovations from integrated circuits and artificial intelligence to photovoltaics.^3,4^ However, the growth of high-quality single crystals with consistent properties and diverse chemistries remains a major challenge in modern science.

In general, single crystals of inorganic materials are grown by high-temperature and high-pressure techniques, such as melt growth or annealing, while organic compounds often crystallize via solution- or vapor-based approaches.^2,5^ However, these methods are not universally compatible, especially for hybrid semiconductors that combine characteristics of both inorganic and organic components, decomposing before melting and exhibiting resistance against solvents.^6,7^ The challenge of dissolving and recrystallizing such hybrids significantly limits the ability to obtain single crystals, which are crucial for structural determination, intrinsic property investigation, and high-performance device development.

An example of such insoluble hybrid semiconductors is metal organochalcogenides (MOCs),^8–12^ an emerging class of low-dimensional semiconductors with remarkable properties, including strong exciton binding (∼400 meV),^13,14^ narrow blue and polarized luminescence,^10,13,16^ in-plane optical anisotropy,^13,17^ and stability against air and moisture.^18–20^ MOCs are also free of heavy metals and have demonstrated ultrastrong and record-high exciton-polariton coupling^21^ as well as p-type charge transport with hole mobilities comparable to metal oxides.^22^ Despite this promise, their electronic and structural characterization remains limited due to poor solubility and challenges in growing high-quality single crystals.

Since 1975,^23^ research groups worldwide have reported that MOCs, especially one-dimensional (1D) and two-dimensional (2D) ones, are largely insoluble due to their polymeric structures and defy conventional crystallization techniques (Table S1).^6,9,19,23–34^ Consequently, single crystals of MOCs are rarely obtained and structural studies of such compounds usually relied on lower-resolution methods like powder X-ray diffraction (PXRD)^35,36^ or advanced, less accessible techniques such as microcrystal electron diffraction (MicroED)^17,37,38^ or X-ray-free-electron-based small-molecule serial femtosecond X-ray crystallography (smSFX).^25,39^

In this work, we report a general strategy for dissolving and recrystallizing insoluble MOCs. Using silver phenylselenide (AgSePh), which resists dissolution in 18 commonly used solvents with various degrees of polarity, functional groups, and acidity, as a model system, we found that polydentate amine-functionalized solvents can dissolve AgSePh, enabling the growth of up to 1 cm single crystals and purer luminescent emission. The finding can be applied to a diverse set of MOC compounds, enabling the structural determination of 15 MOC analogues—including eight previously unreported and three noncentrosymmetric structures. Mechanistic studies indicate that dissolution occurs via particle size reduction into smaller polymer units rather than fully dissociating into ions or soluble molecular species. Overall, this approach not only paves the way for broader structural and functional studies of MOCs and establishes them as a new class of solution-processable semiconductors, but also inspires a transferable strategy for crystallizing other hybrid materials.

## 2 Result and Discussion

### 2.1 MOC’s Crystallization Challenges

Silver phenylselenide (AgSePh), also known as mithrene,^10^ is a prototypical MOC, featuring a polymeric hybrid organic–inorganic structure where covalent metal–chalcogen (Ag–Se) bonds form inorganic layers separated by organic phenyl rings (Figure 1a). This unique structure grants AgSePh exceptional chemical stability^18,20^ but also poses significant challenges for recrystallization. Unlike traditional inorganic semiconductors, the organic content in AgSePh prevents single-crystal formation via melt growth. Heating yellow AgSePh to 300 °C led to its complete decomposition into metallic silver, as confirmed by PXRD (Figure 1b), suggesting that phenyl ring degradation drives this transformation. Differential scanning calorimetry (DSC) and thermogravimetric analysis (Figure S1) further reveal no melting or phase transitions prior to decomposition at 261.9 °C.

**Figure 1:**
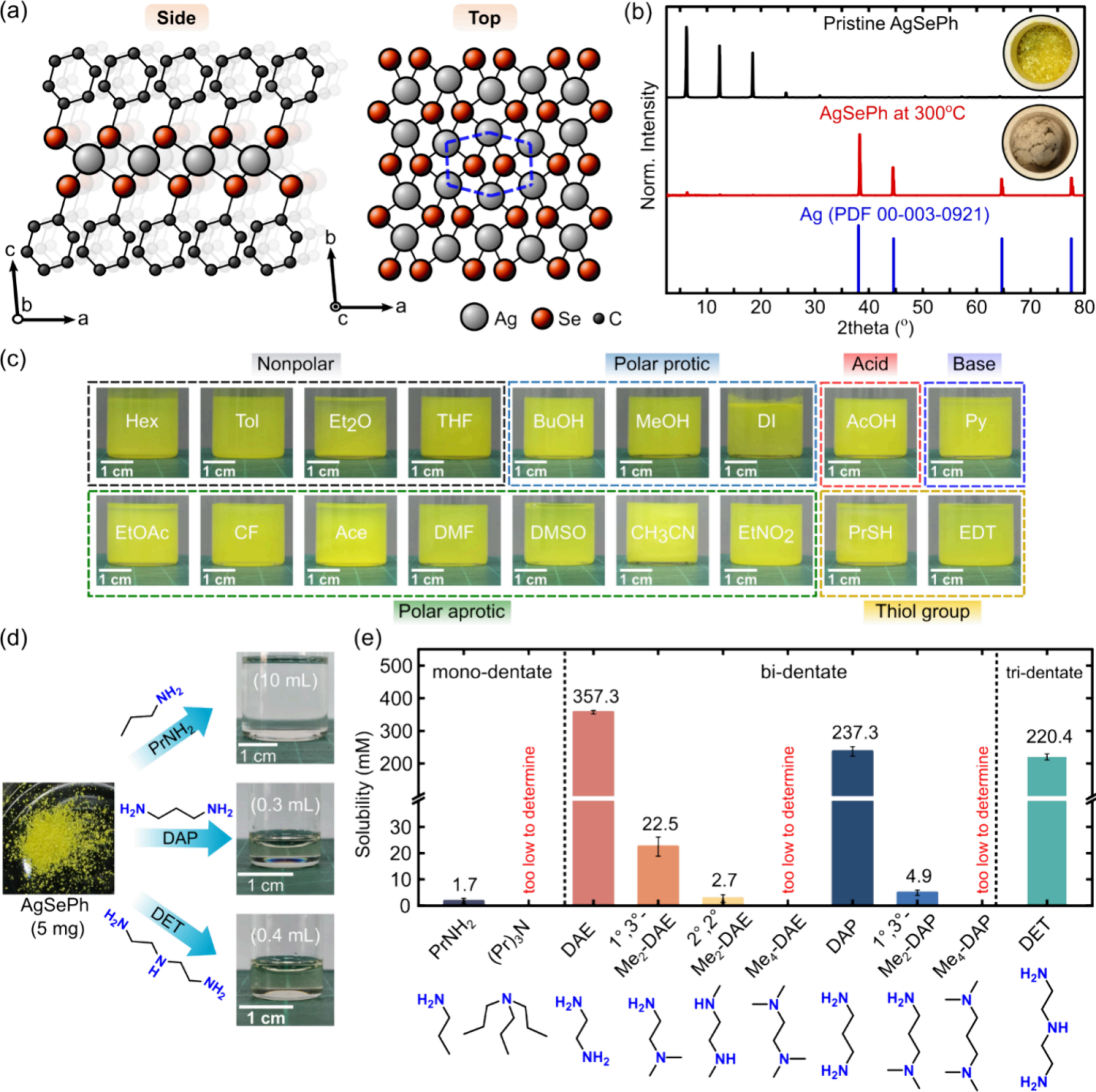
Solvent tolerance of MOCs and solvation strategy. (a) Structure of AgSePh viewed along the side and top (phenyl rings are omitted). (b) X-ray diffraction patterns of AgSePh before and after decomposition at 300 °C, along with the reference pattern of Ag metal. The inset shows optical images of AgSePh before and after decomposition. (c) Insolubility tests of AgSePh in various solvents, including nonpolar, polar protic, polar aprotic, acidic, basic, and thiol-containing solvents. Hex = hexane, Tol = toluene, Et_2_O = diethyl ether, THF = tetrahydrofuran, EtOAc = ethyl acetate, CHCl_3_ = chloroform, Ace = acetone, DMF = dimethylformamide, DMSO = dimethyl sulfoxide, CH_3_CN = acetonitrile, EtNO_2_ = nitroethane, MeOH = methanol, IPA = isopropyl alcohol, DI = deionized water, AcOH = acetic acid, Py = pyridine, PrSH = propanethiol, and EDT = ethanedithiol. (d) Solubility of AgSePh in monodentate propylamine (PrNH_2_), bidentate 1,3-diaminopropane (DAP), and tridentate diethylenetriamine (DET). (e) Bar chart showing the solubility of AgSePh in amines with varying degrees of polydentation and *N*-alkylation. PrNH_2_ = propylamine; (Pr)_3_N = tripropylamine; DET = diethylenetriamine; 1°,3°-Me_2_-DAE = *N*,*N*-dimethyl-1,2-diaminoethane; 2°,2°-Me_2_-DAE = *N*,*N’*-dimethyl-1,2-diaminoethane; Me_4_-DAE = *N*,*N,N’,N’*-tetramethyl-1,2-diaminoethane; DAP = 1,3-diaminopropane; 1°,3°-Me_2_-DAP = *N*,*N*-dimethyl-1,2-diaminopropane; and Me_4_-DAP = *N*,*N,N’,N’*-tetramethyl-1,3-diaminopropane.

Moreover, polymeric AgSePh demonstrates remarkable solvent tolerance, remaining insoluble in over 16 commonly used solvents across nonpolar, polar protic and polar aprotic categories, as well as in organic acids and bases (Figure 1c). These include hexane (Hex), toluene (Tol), diethyl ether (Et_2_O), tetrahydrofuran (THF), ethyl acetate (EtOAc), chloroform (CHCl_3_), acetone (Ace), dimethylformamide (DMF), dimethyl sulfoxide (DMSO), acetonitrile (CH_3_CN), nitroethane (EtNO_2_), methanol (MeOH), isopropyl alcohol (IPA), deionized (DI) water, acetic acid (AcOH), and pyridine (Py). Additionally, it exhibits strong resistance to thiol solvents like propanethiol (PrSH) and ethanedithiol (EDT), which have been applied to dissolve some chalcogenide semiconductors.^41,42^ Notably, AgSePh does not undergo ligand exchange even in excess PrSH, in contrast to AgSPh-*p*F and AgSPh-*p*Cl, which have been reported to undergo ligand exchange through a dissolution-recrystallization mechanism.^35^ While some alkyl-bearing Ag–S MOCs have been noted to be sparingly soluble in hot toluene,^17,30^ we found that this approach does not work for AgSePh or other phenyl-based MOCs (Figure S2).

This extraordinary solvent resistance, combined with the absence of melting behavior, makes conventional single-crystal growth methods – such as melt growth and solution-based recrystallization^2^ – ineffective for AgSePh, as they are for purely inorganic or organic compounds.

### 2.2 Dissolution of MOCs by Polydentate Amines

It has been shown that some insoluble inorganic compounds can dissolve in hydrazine or alkahest solutions, yielding molecular inks for solution-processed thin-film fabrication.^42,43^ Inspired by these discoveries, we challenged the conventional belief that AgSePh and the broader family of MOCs are insoluble (Table S1). Leveraging silver’s affinity for amine groups, we discovered (initially by P. Prayongkul and P. Pattanasattayavong) that AgSePh can be completely dissolved in amine-functionalized solvents, with solubility significantly enhanced by polydentate solvent molecules. For instance, dissolving 5 mg of AgSePh requires ∼10 mL of a monodentate propylamine (PrNH_2_) to achieve a clear solution (Figure 1d). In contrast, employing a bidentate 1,3-diaminopropane (DAP) dramatically improves solubility, allowing dissolution of 5 mg of AgSePh in just 0.3 mL of DAP. Remarkably, this high solubility persists with tridentate solvents; for example, diethylenetriamine (DET) can completely dissolve the same amount of AgSePh in only 0.4 mL.

Further investigation into the solubility of AgSePh reveals a decline as the degree of *N*-alkyl substitution increases, transitioning from primary (1°) to secondary (2°) and tertiary (3°) amines (Figures 1e and S3). For example, while the solubility limit of AgSePh in 1° PrNH_2_ is 1.7 mM, it becomes too low to determine in 3° tripropylamine, (Pr)_3_N. A similar trend is observed upon methylation of bidentate amines. AgSePh displays a high solubility of 357.3 mM in 1,2-diaminoethane (DAE), which decreases sharply to 22.5 mM, 2.7 mM, and too low to determine in *N*,*N*-dimethyl-1,2-diaminoethane (1°,3°-Me_2_-DAE), *N*,*N’*-dimethyl-1,2-diaminoethane (2°,2°-Me_2_-DAE), and *N*,*N,N’,N’*-tetramethyl-1,2-diaminoethane (Me_4_-DAE), respectively. Similarly, the solubility of AgSePh in DAP reduces from 237.3 mM to 4.9 mM and becomes negligible when *N*,*N*-dimethyl-1,2-diaminopropane (1°,3°-Me_2_-DAP) and *N*,*N,N’,N’*-tetramethyl-1,3-diaminopropane (Me_4_-DAP) are used. This observed trend suggests that the steric hindrance around the amine’s N atom is a critical factor influencing AgSePh dissolution.

Furthermore, we found that the solubility of AgSePh was lower in DAP (C3) than in DAE (C2). Although the underlying reason remains unclear, we hypothesize that it arises from an interplay of chelation ability, steric hindrance, and polarity as the carbon chain length of the amine increases.

### 2.3 Recrystallization and Single-Crystal Growth of AgSePh

The discovery of AgSePh’s dissolution in amine solvents opens new opportunities for solution-based single-crystal growth. To explore this potential, we tested various solution-based recrystallization techniques, including recrystallization by slow evaporation, antisolvent vapor diffusion, cooling, and slow antisolvent injection, to AgSePh dissolved in DAP. DAP was chosen over DAE despite a lower solubility due to its higher air stability. All methods returned yellow AgSePh crystals or powders as shown in Figure 2a.

**Figure 2:**
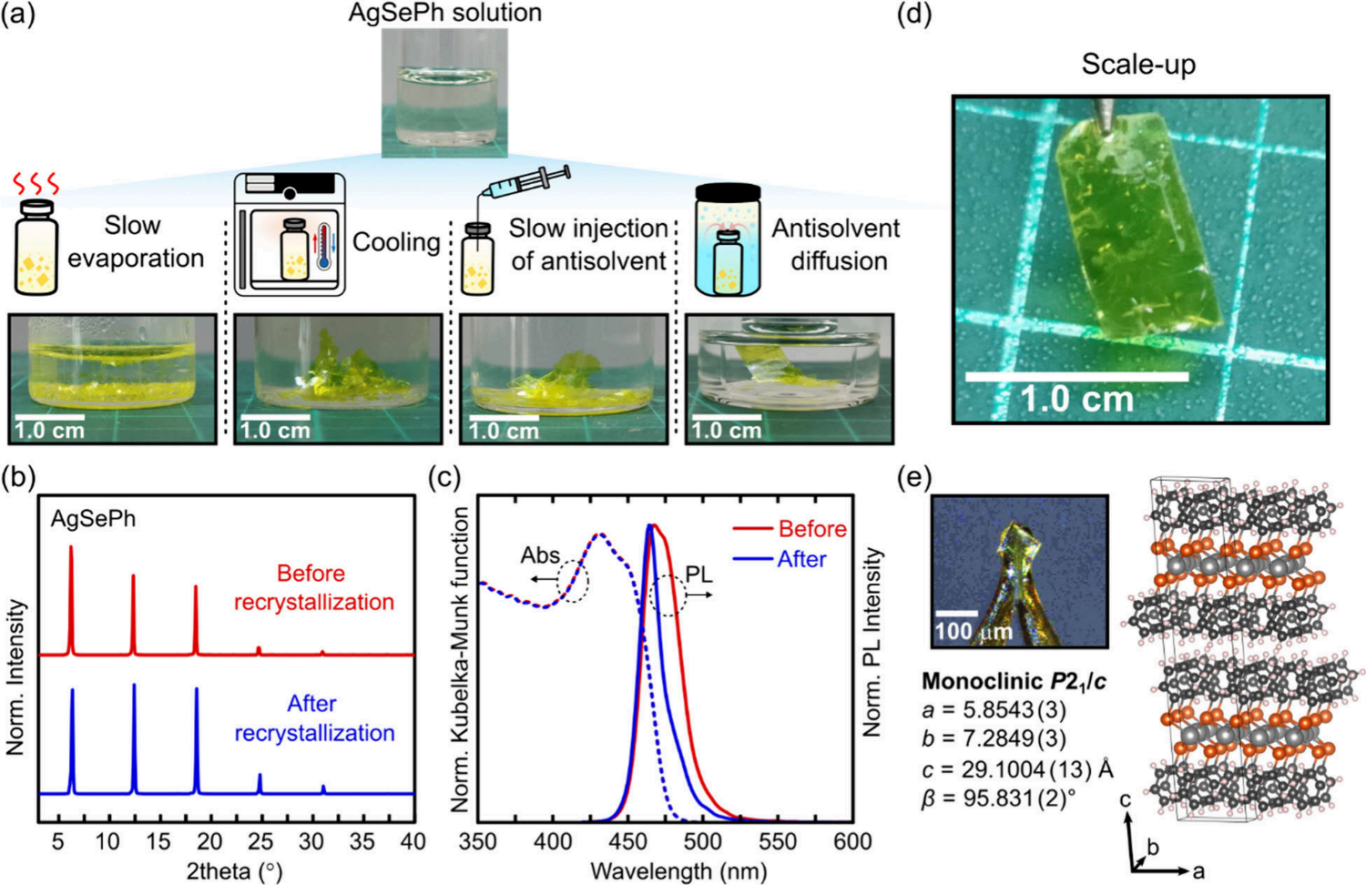
Solution-based recrystallization of AgSePh. (a) Schematic illustration of solution-based recrystallization techniques and photographic images of resulting AgSePh crystals and powders. (b) X-ray diffraction patterns of AgSePh before and after recrystallization by antisolvent diffusion. (c) Optical absorption (Abs; dashed line) and photoluminescence (PL; solid line) of AgSePh before and after recrystallization. (d) Photographic image of centimeter-sized AgSePh crystal obtained by 10x scale-up condition of antisolvent addition recrystallization. (e) An obtained AgSePh single crystal from recrystallization by antisolvent vapor diffusion using *n*-butanol along with its crystal structure and crystallographic information.

PXRD analysis confirmed that recrystallized AgSePh retained the same crystal structure as its original form (Figure 2b), demonstrating that the dissolution in DAP preserves the integrity of the components and does not lead to complete digestion. Additionally, the optical absorption remained unchanged after recrystallization (Figure 2c), further verifying that the chemical identity are preserved. Notably, the photoluminescence (PL) spectrum, which initially exhibited a shoulder peak, became narrower after recrystallization (PL full width at half-maximum decreases from 28 to 16 nm), suggesting a reduction in emissive trap-related states and highlighting the improved optical quality of AgSePh upon recrystallization. Additionally, thermal stability of AgSePh also improved from 261.9 to 278.6 °C after recrystallization (Figure S4).

To obtain AgSePh single crystals, we employed antisolvent vapor diffusion recrystallization (see Section 4 of Supporting Information for rationale) and selected six antisolvents from 18 commonly used solvents screened in Section 2.1, based on boiling points, acid–base compatibility, and chemical inertness (see Section 5 of Supporting Information for further discussion). Among these six representatives—THF (b.p. 66 °C), EtOH (b.p. 78 °C), CH_3_CN (b.p. 82 °C), DI water (b.p. 100 °C), toluene (b.p. 110 °C) and *n*-butanol (b.p. 118 °C)—we observed fewer nucleation events and the formation of larger crystals in high-boiling antisolvents like toluene and *n*-butanol, independent of solvent polarity (Figure S5). We attribute this behavior to the slower diffusion rate of high-boiling antisolvents, which leads to a more gradual decrease in solubility and thus promote controlled crystal growth. After optimization, we successfully obtained AgSePh single crystals using *n*-butanol as the antisolvent. Moreover, the recrystallization process can be scaled up to achieve centimeter-sized AgSePh crystals with dimensions up to 1.02 cm × 0.54 cm (Figure 2d). SCXRD analysis confirmed a layered crystal structure (Figure 2e) free of DAP cocrystallization, and the structure is best described by the monoclinic *P*2_1_/*c* space group, consistent with previous reports by amine-assisted crystallization.^44,45^

### 2.4 Generalization to MOC Derivatives

To evaluate the generality of our dissolution technique, we tested the dissolution and recrystallization in DAP of 18 additional Ag-based MOC derivatives (19 in total including AgSePh), varying in chalcogen composition (S, Se and Te), organic moieties (alkyl, aromatic, heterocyclic aromatic), and substituent groups (alkyl, halide, electron-donating and electron-withdrawing groups) (Figure 3). All derivatives, except AgSPh-*p*OH which degraded in DAP after an overnight storage (Figure S6), demonstrated excellent solubilities, enabling successful recrystallization of MOC via antisolvent vapor diffusion, cooling-induced, or antisolvent injection methods (Figure 3).

**Figure 3:**
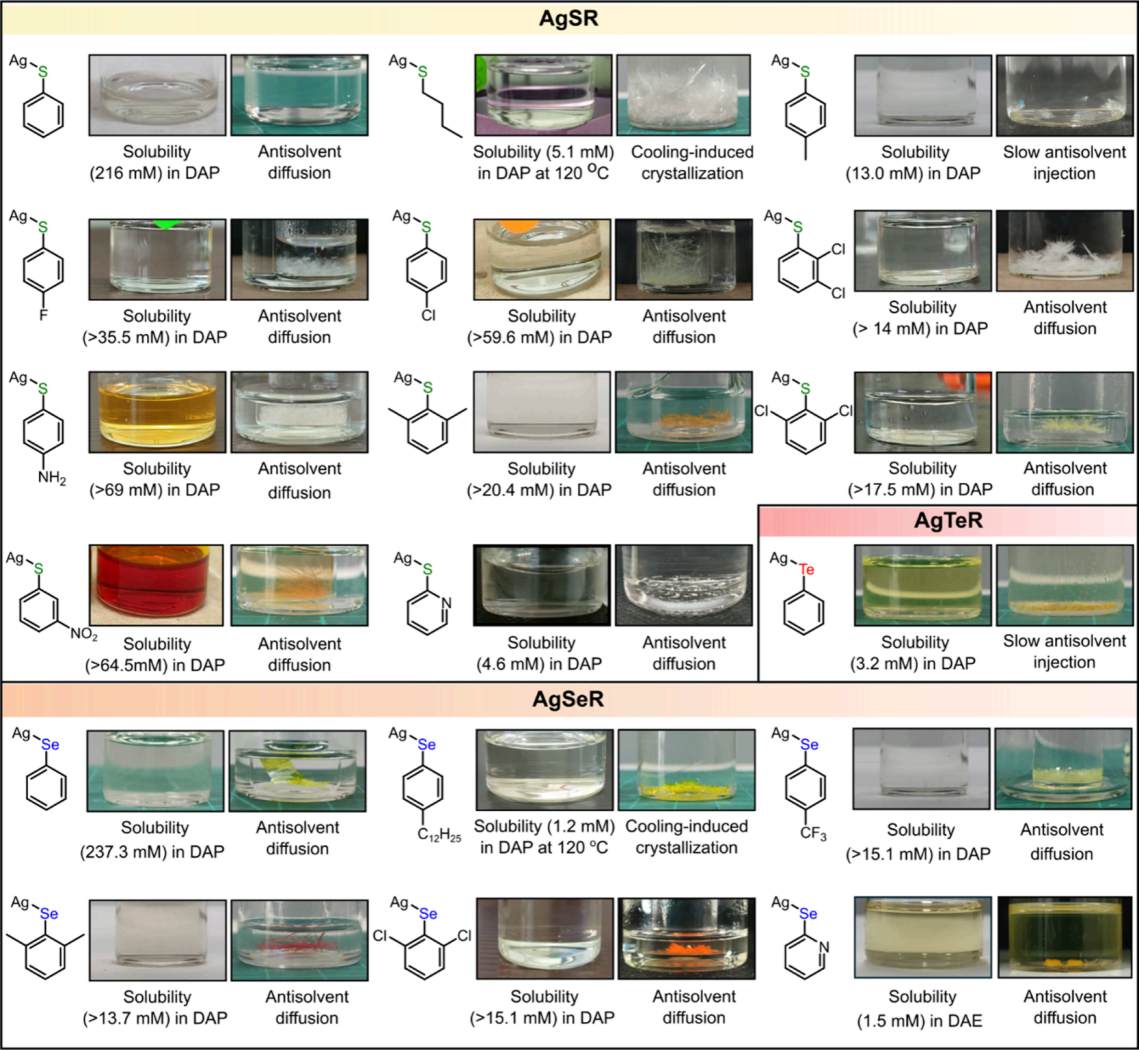
Generalization of amine dissolution to MOC family. Simplified chemical structures and photographic images of tested MOCs with varying chalcogen and organic components before and after recrystallization, along with their solubilities in 1,3-diaminopropane (DAP) and the corresponding recrystallization techniques.

PXRD analysis revealed that 15 of the 18 recrystallized MOCs retained their original structural patterns (Figure S7), indicating no structural or phase changes upon recrystallization. However, three derivatives—AgTePh, AgSPh-*p*NH_2_ and AgSPh-*m*NO_2_—exhibited altered PXRD after recrystallization (Figure S8). For AgTePh, the change in the diffraction pattern (Figure S8a) was caused by the transformation of as-synthesized 1D fibrous phase to a more stable 2D phase (Figure S9a).^45^ In contrast, the as-synthesized AgSPh-*p*NH_2_ appeared as orange-brown powders, which recrystallized into colorless plate-like crystals (Figure S9b). PXRD analysis suggests that the as-synthesized powders likely consisted of a mixture of two 2D MOC phases, with the dominant phase turning into a minor one upon recrystallization (Figure S8b). Similarly, the as-synthesized AgSPh-*m*NO_2_ formed pale-yellow needles that recrystallized into orange 1D needle crystals (Figure S9c), accompanied by a shift in the PXRD pattern toward higher 2θ values (Figure S8c). We attribute the structural changes in AgSPh-*p*NH_2_ and AgSPh-*m*NO_2_ to cocrystallization of solvent/antisolvent, as will be further discussed below and in Section 7 of Supporting Information.

Of the 18 recrystallizable MOCs, 15 yielded single crystals of sufficient size and quality for SCXRD analysis (Figure 4 and Table S2). Notably, AgSPh crystals as large as one centimeter (1.03 cm × 1.08 cm) were obtained using this generalizable approach (Figure S10). SCXRD revealed that all 15 MOCs crystallize in primitive lattice space groups and exhibit diverse structural motifs: eight form two-dimensional (2D) structures, six adopt one-dimensional (1D) structures, and one displays a zero-dimensional (0D) structure. Remarkably, eight are previously unreported, including 2D AgSPh-*p*CH_3_ (*Pbca*), 1D AgSPh-Cl_2_(2,3) (*P*2_1_), 1D AgSPh-Cl_2_(2,6) (*P*2_1_2_1_2), 1D AgSPh-Me_2_(2,6) (*P*2/*c*), 1D AgSePh-Me_2_(2,6) (*P*-1), 1D AgSePh-Cl_2_(2,6) (*P*2_1_/*c*), 2D [AgSPh-*p*NH_2_]_2_·H_2_O (*P*2_1_/*c*), and 1D [AgSPh-*m*NO_2_]_4_·1DAP (*P*2_1_2_1_2_1_) (Table S3).

**Figure 4:**
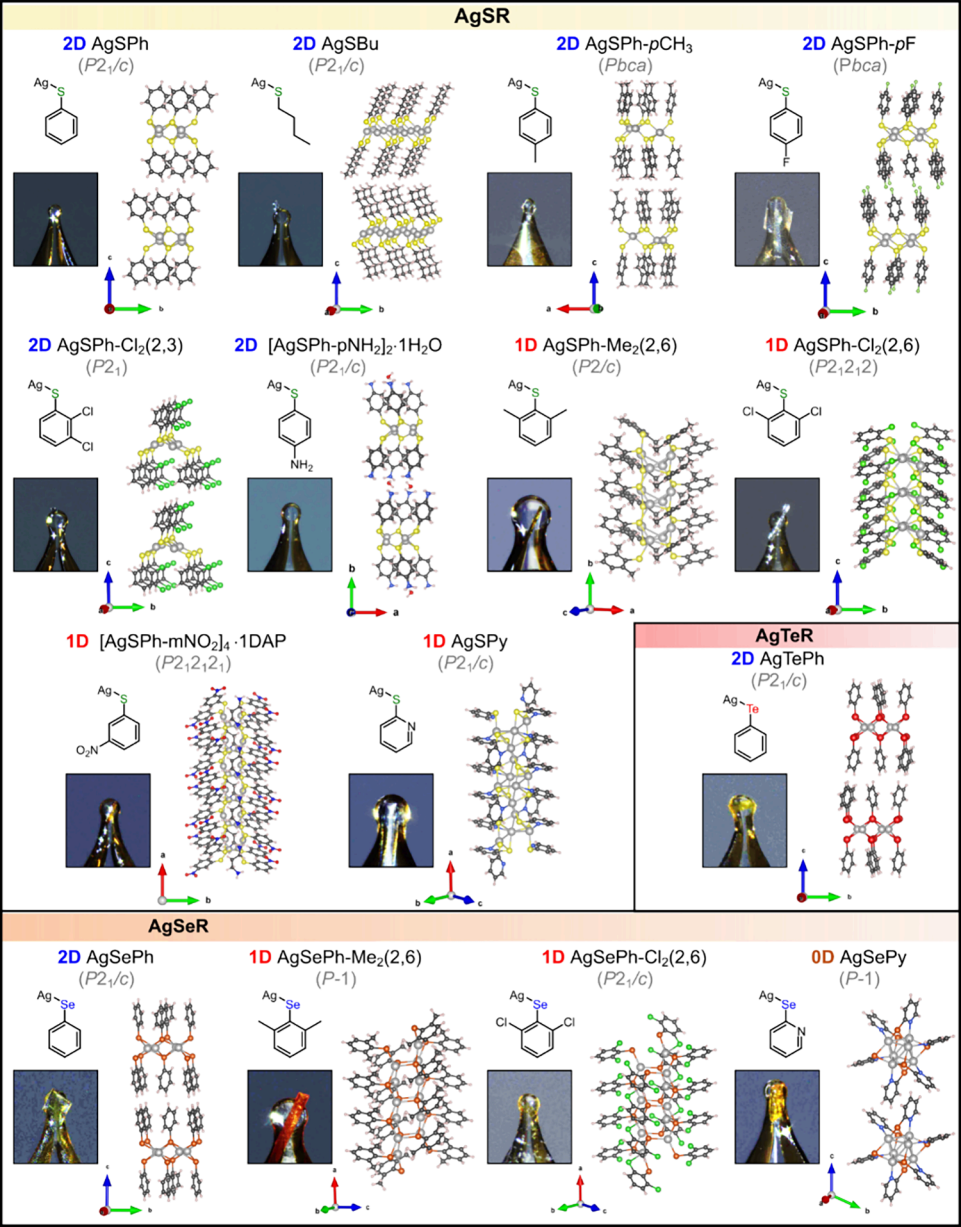
Crystal structures of MOCs enabled by polydentate amine dissolution technique. Crystal structures of recrystallized MOCs, along with their space groups, simplified chemical structures, and photographic images of crystals used for single-crystal X-ray diffraction (SCXRD) characterization.

Of particular interest, AgSPh-Cl_2_(2,6) and [AgSPh-*m*NO_2_]_4_·1DAP adopt the noncentrosymmetric *P*2_1_2_1_2 and *P*2_1_2_1_2_1_ space groups, respectively, while AgSPh-Cl_2_(2,3) crystallizes in the polar, pyroelectric *P*2_1_ space group—both symmetry classes that are prerequisites for emergent properties such as piezoelectricity, pyroelectricity, and ferroelectricity.

Additionally, we confirmed the structures of seven previously reported MOCs. AgEPh (E = Se, Te) and AgSPy crystallize in the *P*2_1_/*c* space group while AgSePy adopts the *P*1̅ space group, consistent with prior SCXRD refinements^20,44–46^ (Table S3). However, our analysis revealed differences for AgSPh, AgSBu and AgSPh-*p*F, which are best described by the *P*2_1_/*c*, *P*2_1_/*c* and *Pbca* space groups, respectively, deviating from prior SCXRD,^45^ small-molecule serial femtosecond X-ray crystallography (smSFX),^25^ and Le Bail refinement assignments^47^ (Table S3). Careful systematic absence analysis confirmed these space group assignments, and our refinements show improved metrics, including higher mean *I/σ*, lower final *R* index, and reduced peak residuals, indicating that our recrystallization technique yielded higher-quality diffraction data (see Section 13 of Supporting Information for further discussion).

Analysis of the refined crystal structures revealed three key structural trends. First, the dimensionality of MOCs is influenced by steric effects associated with ligand substitution. MOCs without substitution (e.g., AgSPh, AgSBu, AgSePh, AgTePh) or with para-substitution (e.g., AgSePh-*p*CH_3_, AgSPh-*p*F, [AgSPh-*p*NH_2_]_2_·H_2_O) typically adopt 2D structures. By contrast, ligands with ortho- or meta-substitution tend to yield 1D structures (e.g., AgSPh-Cl_2_(2,6), AgSPh-Me_2_(2,6), [AgSPh-*m*NO_2_]_4_·1DAP, AgSePh-Cl_2_(2,6), and AgSePh-Me_2_(2,6)). These observations are consistent with our previous report on the 1D structure of AgSePh-F_2_(2,6),^19^ as well as recent studies showing that AgSPh-*p*X (X = OMe, COOMe) adopts 2D structures, whereas AgSPh-*o*X and AgSPh-*m*X favor 1D structures.^36,48^ Between the ortho and meta positions, steric effects are expected to be stronger at the ortho position due to its closer proximity to the inorganic core, thereby promoting formation of lower-dimensional structures. While direct comparisons are not available in this work, Demessence and co-workers reported that AgSPhCOOH forms 2D structures when substituted at the para or meta positions, but only 1D structures when substituted at the ortho position.^36^

Second, intramolecular interactions such as Ag–N bonding can also reduce dimensionality (Figure S11). For instance, AgSPy forms a 1D structure in contrast to the 2D structure of AgSPh, and AgSePy adopts a 0D structure compared to the 2D structure of AgSePh.

Third, cocrystallization with solvents frequently occurs in MOCs bearing strong electron-withdrawing or electron-donating groups, as seen in [AgSPh-*p*NH_2_]_2_·H_2_O and [AgSPh-*m*NO_2_]_4_·1DAP. The incorporation of H_2_O and DAP molecules in these structures is facilitated by H-bonding and Ag–N interactions, respectively (Figure S12).

To further assess the applicability of our dissolution approach, we tested the dissolution and recrystallization in DAP on a Cu-based MOC. We observed that CuSPh dissolves in DAP and can be recrystallized (Figure S13a) while maintaining its PXRD pattern (Figure S13b). Although the recrystallization conditions have not yet been optimized to yield single crystals suitable for SCXRD analysis, this result indicates that our approach could be extended to MOCs incorporating other metals.

In addition to structural elucidation, we examined the optical properties of all synthesized MOCs. These compounds exhibited diverse optical absorption and emission features, tunable from the ultraviolet to near-infrared range (Table S4 and Figures S14–S15). Several compositional and structural trends emerged. First, the optical absorption and bandgaps systematically shifted to lower energy as the chalcogen composition changed from S to Se to Te, in agreement with previous reports on AgSPh, AgSePh, and AgTePh.^13,22,39,45^ We attribute this trend to the larger and more diffuse *p* orbitals of heavier chalcogens, which reduce the energy separation from Ag *d* orbitals and elevate the valence band maximum. This effect is well established in other chalcogenide semiconductors, such as silver chalcogenides, and reflects the progressive increase in chalcogen atomic size and *p* orbital energy.^49,50^

Second, dimensionality strongly influences optical response. In general, 2D MOCs show larger bandgaps and first absorption peaks at shorter wavelengths compared to 1D and 0D analogues. For instance, all 2D S-based MOCs showed first absorption peaks below ∼380 nm, whereas 1D analogues appeared at >400 nm. A similar trend was observed for Se-based compounds, with first absorption peaks of 2D members situated below ∼470 nm and 1D members above ∼ 480 nm. Notably, exceptions exist, such as 1D AgSPy (448–449 nm) and 0D AgSePy (378–381 nm), likely due to additional Ag–N bonding effects.

Third, functional group substitution modulates bandgaps through electronic effects. Electron-withdrawing groups generally increased bandgaps and shifted absorption to shorter wavelengths, while electron-donating groups had the opposite effect. For example, the first absorption peak of 2D AgSePh at 448–449 nm shifted to 418–421 nm in AgSePh-*p*CF_3_ and to 463–466 nm in AgSePh-*p*C_12_H_25_ upon introducing electron-withdrawing CF_3_ and electron-donating C_12_H_25_ groups, respectively. A similar trend was observed for 1D AgEPh-X_2_(2,6) derivatives [E = S, Se; X = Cl, Me], where replacing Cl with Me shifted the first absorption peak from 409–411 nm to 468–473 nm in AgS derivatives, and from 487–493 nm to 550–551 nm in AgSe derivatives. These trends align with prior studies.^47,51^ However, we note that the bandgaps of 2D S-based MOCs in this study cannot be fully rationalized by electronic effects alone, suggesting that additional factors, such as steric interactions, may also play a role.

Photoluminescence (PL) measurements revealed further correlations. On average, 1D and 0D MOCs exhibited stronger PL under ultraviolet excitation compared to their 2D counterparts. Notably, four compounds displayed PL quantum yields (PLQYs) exceeding 10% after recrystallization: 1D AgSPh-Cl_2_(2,6) (34%), 1D AgSePh-Me_2_(2,6) (16%), 1D AgSePh–Cl_2_(2,6) (11%), and 0D AgSePy (54%) (Figure S16). In contrast, 2D compounds generally displayed weak PL, particularly S-based derivatives, which required higher excitation power and slit widths to detect measurable emission.

Furthermore, 1D and 0D MOCs exhibit broader PL line widths and longer decay lifetimes compared to their 2D counterparts (Table S4 and Figures S14, S17). Both S- and Se-based 1D and 0D members showed broad PL full-width-at-half-maximum (fwhm) of at least 96 nm, whereas 2D S-based MOCs exhibited narrower line widths below 93 nm. The reduction in fwhm is more dramatic in the case of 2D Se-based MOCs whose PL fwhm’s were 16–25 nm. Additionally, all 2D Se-based MOCs exhibited fast PL decays with lifetimes as short as sub-ns to below ∼7 ns. On the other hand, the PL decays of 1D and 0D MOCs consist of multiple components where the decay lifetimes of the main components (higher relative percentage) ranged from ∼50 ns up to ∼5 μs.

Finally, we compared the optical properties before and after recrystallization. With the exception of AgTePh, AgSPh-*p*NH_2_, and AgSPh-*m*NO_2_ (which underwent phase transformations), all MOCs retained nearly identical absorption and emission profiles. However, recrystallized samples generally exhibited higher PLQYs and longer lifetimes (Table S4, Figures S16–S17). These improvements are likely attributable to a reduction in defect density and the suppression of fast nonradiative recombination pathways achieved through the recrystallization process.

### 2.5 Mechanism of MOC Dissolution

To elucidate the dissolution mechanism of MOCs, we considered several possible scenarios (Figure 5a). First, MOCs might dissolve like ionic salts (e.g., sodium chloride), with their constituents fully dissociating into ions to form electrolyte solutions. Second, they could behave like molecular systems (e.g., small-molecule organic semiconductors) or some covalent inorganic semiconductors, dissolving through the disruption of inter- and intramolecular interactions into smaller particles. Third, amines may function as reagents, interacting with MOCs to form soluble intermediate species.

**Figure 5:**
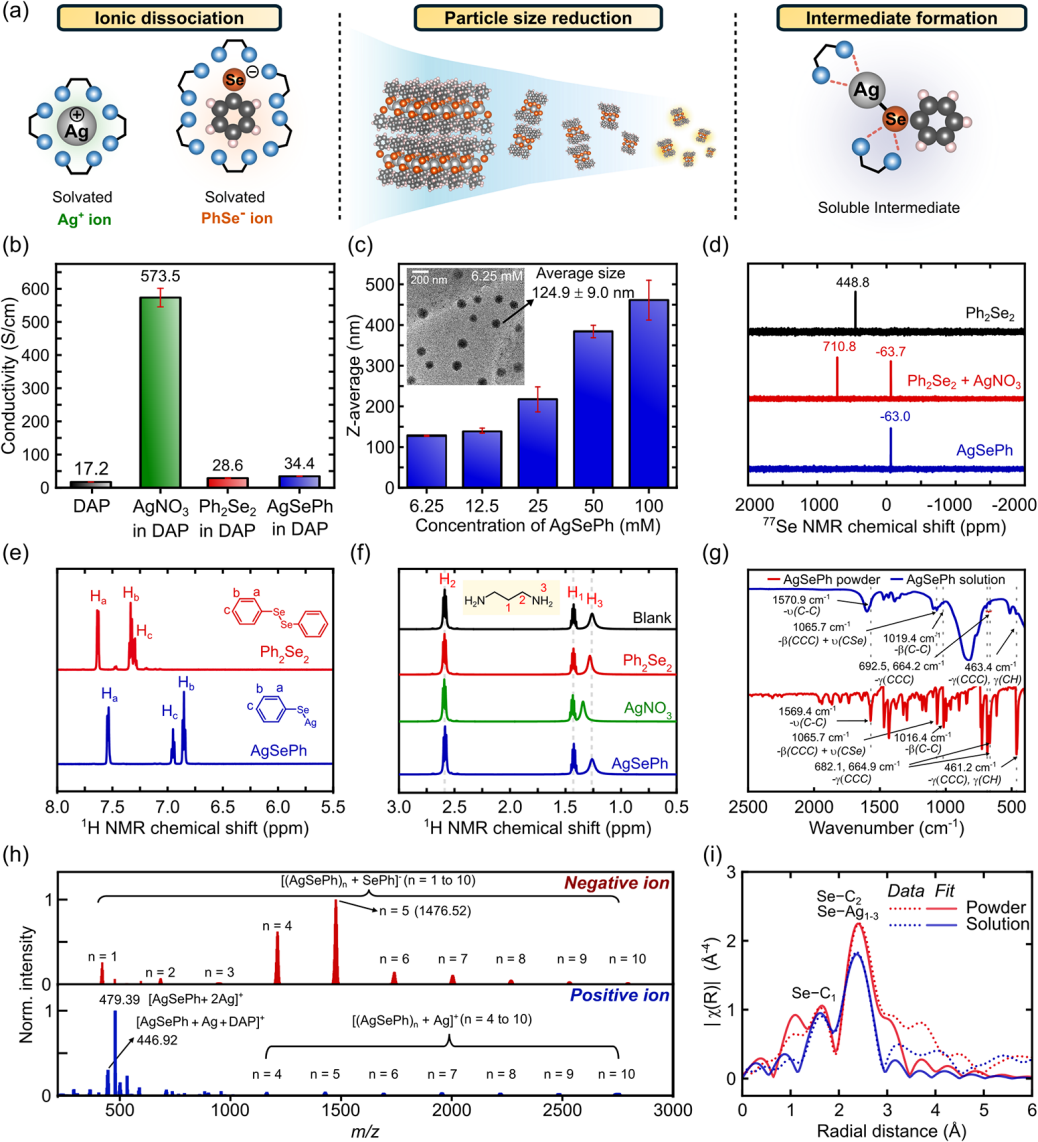
Mechanistic study of AgSePh’s dissolution. (a) Plausible mechanisms of dissolution process via ionic dissociation, particle size reduction, and intermediate formation. (b) Ionic conductivities of pure DAP solvent, and silver nitrate (AgNO_3_), diphenyldiselenide (Ph_2_Se_2_), and AgSePh in DAP. (c) DLS particle size analysis of AgSePh/DAP solutions at various concentrations. Inset shows a TEM image of small particles in the 6.25 mM solution. (d) ^77^Se-nuclear magnetic resonance (NMR) spectra of Ph_2_Se_2_, mixture of AgNO_3_ and Ph_2_Se_2_, and AgSePh in 50% DAP/DMSO-*d*_6_. (e) ^1^H NMR spectra in the aromatic region of Ph_2_Se_2_ and AgSePh in 50% DAP/DMSO-*d*_6_. (f) ^1^H NMR spectra in the aliphatic region of pure DAP, Ph_2_Se_2_ in 50% DAP/DMSO-*d*_6_, AgNO_3_ in 50% DAP/DMSO-*d*_6_, and AgSePh in 50% DAP/DMSO-*d*_6_. (g) FTIR spectra of AgSePh powder and AgSePh dissolved in DAP (200 mM). (h) Positive (blue) and negative (red) ion electrospray ionization mass spectra of AgSePh dissolved in DAP. (i) Extended X-ray absorption fine structure (EXAFS) at the Se–K edge of AgSePh powder and solution along with fittings based on AgSePh crystal structure.

To evaluate these mechanisms, we first conducted ionic conductivity measurements (Figure 5b). The AgSePh solution in DAP exhibited a conductivity of 34.4 μS/cm, only slightly higher than that of Ph_2_Se_2_ in DAP (28.6 μS/cm) and pure DAP (17.2 μS/cm), but significantly lower than AgNO_3_ in DAP at 573.5 μS/cm. This suggests minimal ion formation, ruling out complete dissociation as the primary dissolution mechanism.

Upon laser beam projection, we observed the Tyndall effect in the AgSePh solution (Figure S18), indicating the presence of small, suspended particles. Dynamic light scattering (DLS) analysis (Figure 5c) confirmed the presence of particles with an average size of 461.1 nm in a 100 mM solution of AgSePh in DAP. Further dilution of the solution resulted in successively smaller particles to 127.5 nm at 6.25 mM concentration, consistent with progressive fragmentation. The presence of such suspended particles was further verified by transmission electron microscopy (TEM) of a rapidly dried AgSePh solution under vacuum. The particles in the 6.25 mM AgSePh solution exhibit a spherical shape with an average size of 124.9 nm (Figure 5c inset), consistent with the DLS measurement. Energy dispersive X-ray (EDX) spectroscopy confirmed that these particles contain Ag and Se (Figure S19).

To determine the chemical nature of these species, we employed nuclear magnetic resonance (NMR) spectroscopy. The ^77^Se-NMR spectrum of the AgSePh solution in DAP (Figure 5d) showed a single peak at −63.0 ppm, distinct from both Ph_2_Se_2_ precursor (448.8 ppm) and the selenenamide byproduct (710.8 ppm) reported in amine-assisted MOC synthesis^44^ (Figure S20). This suggests that dissolution does not occur via a reverse reaction of AgSePh to the selenenamide intermediate. Notably, the observed peak falls within the range of NaSeH (−496 ppm)^52^ and cadmium phenylselenolate complexes (−9.9 ppm),^53^ implying a Se-metal coordination environment. The single peak closely matches the one observed in a mixture of AgNO_3_ and Ph_2_Se_2_, confirming the characteristic ^77^Se NMR of soluble AgSePh in solution and ruling out the formation of alternative Se intermediates upon dissolution. The ^1^H NMR spectrum (Figure 5e) revealed upfield-shifted aromatic peaks, including a doublet and two triplets, indicative of shielding by a neighboring metal center. Additionally, the absence of -NH_2_ peak shifts (Figure 5f) contrasts with the Ag^+^–amine complexation observed in AgNO_3_ solution, further supporting that AgSePh does not dissociate completely into Ag^+^ and SePh^–^ ions.

Fourier-transform infrared (FTIR) spectroscopy showed characteristic AgSePh peaks even after dissolution. Figure 5g illustrates the FTIR spectrum of 200 mM AgSePh solution in DAP which exhibits phenyl characteristic of AgSePh at 463.4 cm^–1^ (*(γCCC)*, *γ­(CH))*, 1019.4 cm^–1^ *(β­(CH)),* 1065.7 cm^–1^ *(β­(CCC) + υ­(CSe))*, 1469.4 and 1432.8 cm^–1^ (υ (C–C)), and 1570.9 cm^–1^ (υ (CCC)) over the background solvent signal. After removing the contribution from DAP, these peaks shift to 466.4 cm^–1^ *(γCCC)*, 1020.1 cm^–1^ *(β­(CH)),* 1067.9 cm^–1^ *(β­(CCC) + υ­(CSe))*, 1469.4 and 1433.6 cm^–1^ (υ (C–C)), and 1572.4 cm^–1^ (υ (CCC))_,_ closely matching those observed in AgSePh powder (Figure S21). This suggests that the structure of AgSePh remains mostly intact despite particle size reduction.

Electrospray ionization mass spectrometry (ESI-MS) analysis reveals a series of small AgSePh fragments after dissolution (Figure 5h). A negative ion ESI-mass spectrum of AgSePh solution in DAP displays equally spaced *m*/*z* peaks corresponding to a series of AgSePh polymer units bound to an SePh^–^ ion ([(AgSePh)_n_ + SePh]^−^ where n = 1, 2, 3, 4,···), while the positive ion spectrum exhibits a similar pattern with [(AgSePh)_n_ + Ag]^+^ ions and additional major peaks at *m*/*z* = 446.9 and 479.3 corresponding to [AgSePh + Ag + DAP]^+^ and [AgSePh + 2Ag]^+^. Given that ESI-MS is a soft ionization technique that typically induces minimal fragmentation,^54,55^ these results suggest that polymeric AgSePh structure remains largely intact in solution. However, since ion bombardment during measurement could generate charged species, the predominant dissolved form is more likely neutral (AgSePh)_n,_ consistent with ionic conductivity and NMR findings.

To further investigate the structural integrity of dissolved AgSePh, we then employed synchrotron-based extended X-ray absorption fine structure (EXAFS) spectroscopy (Figure 5i). Local coordination environments of Se atoms in AgSePh before and after amine dissolution were examined using *k* ^3^-weighted Fourier-transform Se K-edge EXAFS analysis. The spectrum of AgSePh in DAP closely resembled that of the powder, indicating that the local coordination environment remains largely unchanged upon dissolution. Both powder and solution spectra were fitted with simulated curves based on the crystal structure of AgSePh (Figure S22). Quantitative structural comparison from EXAFS fitting results (Table S5 **)** shows that the coordination number of Se remains the same between the powder and solution forms, further supporting that the local structure around Se is preserved upon dissolution. Notably, the Debye–Waller factors (σ^2^) increased in the solution form, suggesting a higher degree of structural disorder,^56^ likely due to the absence of rigid crystal packing in solution. While Se–C bond distances (C1, C2) exhibit only minor changes, all Se–Ag bond distances (Ag1, Ag2, Ag3) are significantly shorten in solution compared to the powder, potentially reflecting disruption of long-range Ag–Se order in the dissolved state.

Considering all experimental evidence, we therefore propose that AgSePh dissolves in DAP by fragmentation into smaller, neutral polymer units of [AgSePh]_n_, rather than fully dissociating into ions or soluble molecular species.

## 3 Conclusion and Outlook

In conclusion, this work presents a significant advancement in the understanding of MOC solubility, overcoming the longstanding challenges in crystallization and structural determination associated with their hybrid organic–inorganic nature. We demonstrate that AgSePh, a representative MOC, exhibits remarkable solubility in amine-based solvents, particularly in polydentate amines, where solubility is enhanced by chelation and limited by steric effects. The observed solubility in amines is general across 19 tested MOC derivatives with diverse chalcogens, organic backbones, and substituents. Notably, 15 derivatives were successfully grown into single crystals using solution-based recrystallization methods, enabling the structural elucidation of eight novel members and confirmation of seven previously reported species. Among them, AgSePh and AgSPh crystals were readily grown to sizes as large as 1.02 cm × 0.54 cm and 1.03 cm × 1.08 cm, respectively, highlighting the scalability of this approach. Furthermore, AgSPh-Cl_2_(2,6) and [AgSPh-*m*NO_2_]_4_·1DAP crystallize in a noncentrosymmetric space group while AgSPh-Cl_2_(2,3) adopts a polar, pyroelectric space group—both symmetry classes that are essential for emergent properties such as piezoelectricity, pyroelectricity, and ferroelectricity.

Mechanistic investigations combining conductivity measurements, DLS, NMR, FTIR, MS, and EXAFS, reveal that dissolution proceed via fragmentation into smaller, charge-neutral polymeric [AgSePh]_n_ units, rather than ionic dissociation. This insight not only deepens the fundamental understanding of MOC solubility but also establishes a versatile platform for single-crystal growth and the rational design of advanced MOC-based materials.

Importantly, this discovery also introduces MOCs as a new family of solution-processable semiconductors, enabling low-temperature, low-cost fabrication techniques. Together with recent reports on optical anisotropy,^13,17^ ultrastrong exciton-polariton coupling,^21^ and high hole mobilities^22^ —as well as our finding that several MOCs adopt 2D layered, noncentrosymmetric architectures—these results position MOCs as promising candidates for next-generation optoelectronic and quantum materials.

Overall, we anticipate that these results will catalyze further research into chemistry, physics, and device applications of hybrid organic–inorganic materials – especially MOCs – as well as inspire broader effort to render other previously insoluble materials accessible through solution-based crystallization strategies.

## 4 Experimental Section

### 4.1 Chemicals

All chemicals and solvents were used as received without further purification. Silver nitrate (AgNO_3_), diphenyl diselenide (Ph_2_Se_2_), propylamine (PrNH_2_) and ethylene diamine (EDA) were purchased from Thermo Fisher Scientific. 1,3-diaminopropane (DAP), diethylenetriamine (DET) dipropylenetriamine (DPT) and all derivatives of disulfide (RS-SR), diselenide (RSe-SeR) and ditelluride (RTe-TeR) organic precursors were purchased from Tokyo Chemical Industry (TCI), Thermo Fisher Scientific and Sigma-Aldrich. Dipyridinyl diselenide (Py_2_Se_2_), 2,6-dimethylphenyl diselenide and 2,6-dichlorophenyl diselenide were synthesized from 2-bromopyridine, 1-bromo-2,6-dimethylbenzene and 1-bromo-2,6-dichlorobenzene by methods reported in previous work.^19,20^ General A.R. grade organic solvents were purchased from TCI, Sigma-Aldrich, Merck, Qrec, DAEJUNG, and Fisher Scientific.

### 4.2 Synthesis and Recrystallization Methods

#### 4.2.1 Synthesis of AgSPh, AgSePh and AgTePh Microcrystalline Powders

AgSPh, AgSePh, and AgTePh microcrystalline powders were synthesized via an amine-assisted single-phase reaction.^44^ Briefly, a 100 mM solution of diphenyl dichalcogenide (Ph_2_E_2_, E = S, Se, Te) in toluene was mixed in equal volume with a 100 mM solution of AgNO_3_ in an amine solvent – propylamine for AgSPh and AgSePh, and butylamine for AgTePh. The mixture was stirred at ambient conditions for 2 h, yielding white (AgSPh), yellow (AgSePh), and red (AgTePh) microcrystalline powders. The products were isolated by centrifugation, washed with toluene and isopropanol, and dried under vacuum at 50 °C overnight.

#### 4.2.2 General Synthesis Method for MOC Derivatives (AgER)

MOC derivatives (AgER) were synthesized following the same procedure as unfunctionalized AgEPh with slight modifications. The organic dichalcogenide precursor was dissolved in an appropriate solvent (toluene, methanol, or tetrahydrofuran) to form a 10 mM solution, before mixing in equal volume with a 10 mM solution of AgNO_3_ in propylamine. The reaction mixture was stirred at ambient conditions for 2–24 h, yielding microcrystalline MOC powers. The products were isolated by centrifugation, washed with toluene and isopropanol, and dried under vacuum at 50 °C overnight.

#### 4.2.3 Recrystallization by Antisolvent Vapor Diffusion and Slow Antisolvent Injection

MOC microcrystalline powders were dissolved in DAP and filtered through a 0.22 μm syringe filter to obtain a clear solution. For the antisolvent vapor diffusion method, a 1 mL aliquot of the filtered solution was transferred into an uncapped 4 mL vial, which was then placed inside a capped 20 mL vial containing 3 mL of an antisolvent. Crystals formed over 3 – 14 days. For structural determination by SCXRD, the crystals were directly collected from the solution without further purification. For other characterization techniques, the crystals were isolated, washed with IPA, and dried at 50 °C under vacuum before use.

Alternatively, some MOC derivatives were recrystallized using a slow antisolvent injection method. In this approach, the antisolvent was introduced at a controlled infusion rate of 0.1 mL/h using a programmable syringe pump.

#### 4.2.4 Recrystallization by Slow Cooling

MOC microcrystalline powders were dispersed in *n*-butanol and heated to 120 °C. Then, DAP was gradually added until a clear solution was obtained. The solution was maintained at 130 °C for 30 min before being cooled to room temperature at a rate of 10 °C/h. For MOC derivatives with low solubility, such as AgSBu and AgSePh-*p*C_12_H_25_, pure DAP was used instead of mixed DAP/*n*-butanol.

#### 4.2.5 Recrystallization by Slow Evaporation

MOC microcrystalline powders were dissolved in DAP and filtered through a 0.22 μm syringe filter to obtain a clear solution. The filtered solution was allowed to slowly evaporate in a fume hood under an airflow rate of approximately 125–140 fpm.

### 4.3 Characterization

#### 4.3.1 Solubility Measurement of MOCs in Different Amine Solvents

The solubility of each MOC compound was determined by measuring the amount of dissolved MOC in the saturated supernatant. Briefly, excess MOC (∼100 mg/mL) was added to an amine solvent to create a saturated solution. The mixture was sonicated for 30 min and then allowed to settle for 1 h to reach equilibrium between the solid and liquid phases. After equilibration, 1 mL of the saturated solution was centrifuged at 5,000 rpm for 10 min. The supernatant was carefully transferred to a preweighed container, followed by the addition of 6 mL of a 1:1 isopropanol/hexane mixture to fully precipitate the dissolved MOC. The precipitate was collected by centrifugation, dried by solvent evaporation at 90 °C for 12 h, and weighed to determine solubility. Each solubility measurement was performed in triplicate to obtain mean and standard deviation values.

#### 4.3.2 Differential Scanning Calorimetry (DSC)

DSC analysis was conducted using a Mettler Toledo DSC823e instrument. The samples underwent three thermal cycles within a temperature range of 10–210 °C, with heating and cooling rates of 10 °C/min under a nitrogen flow rate of 20 mL/min.

#### 4.3.3 Thermogravimetric Analysis (TGA)

TGA was performed using a Rigaku Thermo plus EVO2 instrument. Samples were heated from room temperature to 500 °C at a rate of 10 °C/min under a nitrogen flow of 200 mL/min.

#### 4.3.4 Powder X-ray Diffraction (PXRD)

PXRD patterns were acquired using a Bruker D8 ADVANCE diffractometer equipped with a Cu *K*α radiation (λ = 1.5418 Å) operated at 40 kV and 40 mA. Measurements were performed with a step size of 0.05° and a scanning rate of 0.6°/min.

#### 4.3.5 Single-Crystal X-ray Diffraction (SCXRD)

X-ray diffraction data were collected using a Bruker-AXS D8 Venture diffractometer equipped with a IμS microsource and a Photon 3 CPAD detector, using Mo *K*α radiation (λ = 0.71073 Å). Data acquisition was performed using φ and ω scans. The crystal structures were processed in APEX5 and solved in OLEX2 program^57^ using dual-space methods with SHELXT^58^ and refined against *F* ^2^ using full-matrix least-squares with SHELXL-2017.^59^ All non-hydrogen atoms were refined anisotropically, while hydrogen atoms were placed at geometrically calculated positions and refined using a riding model. VESTA was used to prepare simulated PXRD patterns and material graphics for visualization.

Details on data quality and refinement residuals for all structures are summarized in Table S4. Additional crystallographic information, including atomic coordinates, isotropic and anisotropic displacement parameters, bond lengths, bond angles, torsion angles and hydrogen atom coordinates for all structures can be found in the summary of crystallographic information below in the Supporting Information (Tables S13–S100).

#### 4.3.6 UV–vis Absorption Spectroscopy

Optical absorption spectra were acquired using a PerkinElmer Lambda 1050 spectrophotometer equipped with an integrating sphere in diffuse reflectance mode. Solid samples were prepared by grinding with dry potassium bromide (KBr) to achieve an ∼1 wt % dilution, and diffuse reflectance spectra were measured and calibrated against a 100% KBr baseline. The obtained spectra were then converted into absorption spectra using the Kubelka–Munk transform:^60^

$$F\left(R\right)=\frac{{\left(1-R\right)}^{2}}{2R}$$

where F­(R) is the Kubelka–Munk function with a value proportional to the sample’s absorption coefficient, and R is the relative reflectance of the sample with the KBr baseline.

#### 4.3.7 Photoluminescence (PL) Measurements

Steady-state PL spectra of all MOC samples were obtained using an Edinburgh FLS980 spectrometer equipped with a Xe1 xenon lamp as the excitation source and a single photon counting PMT detector. Excitation wavelengths of 375 and 400 nm were used for Ag–S and Ag–Se/Ag-Te-based MOCs, respectively.

#### 4.3.8 Time-Resolved PL Spectroscopy

Time-resolved PL measurements were performed using the same Edinburgh FLS980 spectrometer as for steady-state PL. Excitation was provided by a variable repetition-rate 404.4 nm picosecond pulsed laser diode (EPL405, Edinburgh Instruments) with an average power of 5 mW.

#### 4.3.9 Photoluminescent Quantum Yield (PLQY)

PLQY measurements were conducted at room temperature using the absolute quantum yield method with an integrating sphere integrated into the Edinburgh FLS980 spectrometer. MOC samples were placed at the center of the integrating sphere and excited at 380 nm using a Xe1 xenon lamp.

#### 4.3.10 Conductivity Measurement

Solution conductivity was measured using a Consort C1020 multiparameter analyzer. The conductivity probe was calibrated with a standard NaCl solution before measurements. All samples were prepared by dissolving in DAP at a fixed concentration of 100 mM.

#### 4.3.11 Dynamic Light Scattering (DLS)

The particle size of AgSePh dissolved in DAP was measured using Malvern Zetasizer Lab dynamic light scattering analyzer at 25 °C with a 633 nm He–Ne laser. Samples were prepared by dissolving AgSePh in DAP at concentrations of 100, 50, 25, 12.5, and 6.25 mM.

#### 4.3.12 Transmission Electron Microscopy (TEM)

The size, morphology and elemental composition of the AgSePh particles were analyzed using a JEOL JEM-ARM200F transmission electron microscope equipped with energy-dispersive X-ray (EDX) spectroscopy and operated at 200 kV. TEM samples were prepared by depositing 3 μL of a 6.5 mM AgSePh solution in DAP onto copper grids, followed by immediate vacuum drying using JEOL JEC-4000DS dry pumping station for 5 min.

#### 4.3.13 Nuclear Magnetic Resonance (NMR) Spectroscopy

^1^H, and ^77^Se NMR spectra were recorded using a Bruker 600 MHz AVANCE III HD spectrometer. Chemical shifts (δ) are reported in parts per million (ppm) relative to the referenced tetramethylsilane (TMS).

#### 4.3.14 Fourier Transform Infrared (FTIR) Spectroscopy

FTIR spectra of AgSePh powder, DAP, and a 200 mM AgSePh solution in DAP were recorded using a PerkinElmer Frontier FTIR spectrometer in attenuated total reflection (ATR) mode with a Universal-ATR accessory.

#### 4.3.15 Electrospray Ionization Mass Spectrometry (ESI-MS)

Mass spectra of AgSePh solution in DAP (3.8 mM) were measured in both positive and negative ion analysis using electrospray ionization in Bruker Compact QTOF LC-Quadrupole-Time-of Flight Tandem mass spectrometer (LC-QTOF-MS).

#### 4.3.16 Synchrotron-Based X-ray Absorption Spectroscopy (XAS)

X-ray absorption spectra covering both the near edge structure (XANES) and the extended fine structure (EXAFS), were conducted using fluorescent mode in the energy region for the Se K-edge (12,659.5 eV) on the beamline BL5.2 at 1.2 GeV-Synchrotron Light Research Institute (SLRI), Thailand. Each powder sample was ground-mixed with boron nitride and packed into Kapton sleeves for measurements. The solution sample was sealed in a plastic film bag for measurements. The spectra analysis processes included background subtraction, normalization, and χ­(k) isolation. The EXAFS signals were extracted by *k* ^3^-weighted Fourier transform using the Hanning function. All spectra were averaged from three scans and analyzed by the standard procedure using Athena and Artemis software.

## Supplementary Material



## Notes

List of previous reports on insolubility of MOCs, additional characterizations on MOC’s solubility and crystallization challenges, additional characterizations of AgSePh before and after recrystallization, selection of crystallization methods, antisolvent selection for antisolvent vapor diffusion recrystallization, additional observation and structural characterizations of functionalized Ag-based MOC derivatives, additional discussion on the structural and optical changes, crystallographic information, additional structural figures, generalization of the dissolution approach to Cu-based MOCs, optical characterizations of functionalized Ag-based MOC derivatives, confirmation of space group assignment, additional crystallographic information (PDF)
