# Supplementary material for: Chelation-Driven Dissolution and Single-Crystal Growth of Hybrid Metal Organochalcogenide Semiconductors by Polydentate Amines
Source: J Am Chem Soc. 2025 Oct 4;147(41):37242–54. doi: 10.1021/jacs.5c10260 (PMC12532297; doi:10.1021/jacs.5c10260)
Supplement: Supplementary file 1 [file ja5c10260_si_001.pdf]

Supporting Information for:

Chelation-Driven Dissolution and Single-Crystal Growth of Hybrid Metal Organochalcogenide Semiconductors by Polydentate Amines

Rattapon Khamlue[†,1], Petcharaphorn Chatsiri[†,1,2], Tomoaki Sakurada[3,4], Jesadaporn Chotimook[1] Pimpan Leangtanom[1], Pongkamon Prayongkul[1], Thassanant Atithep[5], Jintara Padchasri[6], Pinit Kidkhunthod[6,7], Martin Vacha[3], Pichaya Pattanasattayavong[1], Watcharaphol Paritmongkol[1]*

[1]Department of Materials Science and Engineering, School of Molecular Science and Engineering, Vidyasirimedhi Institute of Science and Technology (VISTEC), Rayong 21210, Thailand

[2]Department of Chemical and Biomolecular Engineering, School of Energy Science and Engineering, Vidyasirimedhi Institute of Science and Engineering (VISTEC), Rayong 21210, Thailand

[3]Department of Materials Science and Engineering, Institute of Science Tokyo, Ookayama 2-12-1, Meguro-ku, Tokyo 152-8552, Japan

[4]Yokohama Technical Center, AGC Inc., Yokohama, Kanagawa 230-0045, Japan

[5]Frontier Research Center (FRC), Vidyasirimedhi Institute of Science and Technology (VISTEC), Rayong, 21210 Thailand

[6]Synchrotron Light Research Institute (Public Organization), 111 University Avenue, Muang, Nakhon Ratchasima 30000, Thailand

[7]Department of Chemical Engineering, Faculty of Engineering, Chulalongkorn University, Bangkok 10330, Thailand

*Correspondence to: watcharaphol.p@vistec.ac.th

† These authors contributed equally and are cofirst authors.

Table of Content

1. Reported Insolubility of MOCs 3

2. Additional Characterizations on MOC's Solubility and Crystallization Challenges 6

3. Additional Characterizations of AgSePh Before and After Recrystallization. 9

4. Selection of Recrystallization Methods 10

5. Antisolvent Selection for Antisolvent Vapor Diffusion Recrystallization 11

6. Additional Observation and Structural Characterizations of Functionalized Ag-Based MOC Derivatives 12

7. Additional Discussion on the Structural and Optical Changes in AgTePh, AgSPh-*p*NH_2 and AgSPh-*m*NO_2 16

8. Crystallographic Information 17

9. Additional Structural Figures. 23

10. Generalization of the Dissolution Approach to Cu-Based MOCs 24

11. Optical Characterizations of Functionalized Ag-Based MOC Derivatives 25

13. Confirmation of Space Group Assignment 36

14. Additional Crystallographic Information 45

15. References 118

1. Reported Insolubility of MOCs

Table S1. Summary of reports on the insolubility and difficulty in crystallization of 1D and 2D MOCs

Group	Year	Statement	Reference
G. N. Schrauzer	1975	"A common feature of all the complexes[a] is their insolubility in common organic and inorganic solvents."	Ref. 1
Ian G. Dance	1983	"Compounds AgSR[b] have been known and used for a long time, but with little definitive information about their structures."	Ref. 2
Dennis G. Tuck	1986	"The copper(I) thiolates are generally insoluble in organic solvents"	Ref. 3
Jon Zubieta	1987	"Neutral homoleptic silver thiolate complexes are frequently insoluble, forming nonmolecular aggregates $[AgSR]_{\infty}$[b]"	Ref. 4
Dennis G. Tuck	1988	"The resultant thiolato complexes are insoluble in the common organic solvents"	Ref. 5
Susumu Kitagawa	1990	"The insolubility of this material[c] made purification difficult"	Ref. 6
Ian G. Dance	1991	"The insoluble silver thiolate compounds $AgS(CH_2)_2CH_3$ (1), $AgS(CH_2)_3CH_3$ (2), $AgS(CH_2)_3CH_3$ (3), $AgS(CH_2)_7CH_3$ (4), $AgSC_6H_3$ (5), $AgSC_6H_4$-4-F (6), $AgSC_6H_4$-4-Cl (7), $AgSC_6H_4$-4-Br (8), $AgSC_6H_4$-4-CH_3 (9) and $AgSC_6H_4$-4-OCH_3 (10) have not yielded crystals suitable for full diffraction analysis, but do reveal the essential features of their two-dimensionally nonmolecular structure in their powder diffraction patterns."	Ref. 7
Markus Baumgartner	1993	"Insolubility makes it difficult to obtain crystals[d] with a quality sufficient for diffraction analysis"	Ref. 8
Michael J. Natan	1997	"Unfortunately, the extreme insolubility of neutral AgSR[b] compounds with primary alkyl groups precludes measurement of solution state optical spectra for T, G/T, and G once isolated."	Ref. 9
Pablo Espinet	1999	"Copper thiolates CuSR are still incompletely characterized structurally, largely due to their insolubility and poor crystal habit, which are presumed to be the result of polymeric structures"	Ref. 10
A. N. Parikh	1999	"The compounds[b] appeared highly insoluble in all typical organic solvents including hexane, ethanol, tetrahydro- furan, ether, acetone, toluene, trichlorobenzene, and carbon tetrachloride up to the boiling point, with the exception of hot toluene which	Ref. 11

Group	Year	Statement	Reference
		appeared to dissolve all compounds, though sparingly, upon vigorous stirring for several hours.”	
Albert S.C. Chan	2002	“As a result of the tendency of thiolates to bridge metal centers to yield insoluble or sparingly soluble polymers, it is difficult to control the reactions of thiolates with metal ions and obtain single crystals of polymeric metal thiolates suitable for X-ray diffraction analysis”	Ref. 12
John F. Corrigan	2002	“Crystals of 2[e] are insoluble in common solvents.”	Ref. 13
Chi-Ming Che	2008	“Polymeric homoleptic copper(I) arylthiolates $[Cu(p\text{-}SC_6H_4\text{-}X)]_\infty$ (X=CH_3 (1), H (2), CH_3O (3), *t*Bu (4), CF_3 (5), NO_2 (6), and COOH (7)) have been prepared as insoluble crystalline solids in good yields (75–95 %)”	Ref. 14
Chi-Ming Che	2010	“This insoluble solid compound[f] exhibits a platy morphology and has a bulk electrical conductivity of up to 120 S cm^{-1}”	Ref. 15
Jianping Xie	2012	“The first step was the reduction of Au(III) to Au(I) by GSH followed immediately by the coordination of Au(I) to the thiol group to form insoluble aggregates of Au(I)−thiolate complexes.”	Ref. 16
Aude Demessence	2015	“As a consequence, as opposed to gold thiomalate, $[Au(SPh)]_n$ compound is insoluble in water and any organic solvent”	Ref. 17
Vimal K. Jain	2015	“The homoleptic silver chalcogenolate complexes are polymeric and are insoluble in common organic solvents, thus limiting their utility as precursors for the preparation of silver-chalcogenides”	Ref. 18
Aude Demessence	2016	“One reason for this lack of studies is the difficulty in structurally characterizing these compounds because of the high reactivity of thiol molecules with gold precursors resulting in the formation of insoluble and poorly crystalline precipitates”	Ref. 19
Aude Demessence	2018	“Nevertheless, the neutral MOC polymeric species are mostly insoluble due to their non-molecular structure, and the formation of large enough single crystals has been challenging”	Ref. 20
J. Nathan Hohman	2020	“The crystalline products[g] were insoluble in common solvents such as water, isopropyl alcohol, and ethanol”	Ref. 21

Group	Year	Statement	Reference
Aude Demessence	2020	"Therefore, our refinements indicate that for the amorphous sample 1a[h], the connectivity of Au–S corresponds to double helices with a coherence length of ~9 ˚A and not to tetrameric species. This hypothesis is supported by the insolubility of 1a, since small oligomeric molecules are likely to be soluble"	Ref. 22
J. Nathan Hohman	2023	"However, a lack of a single-crystal structure because of small crystal sizes is a common refrain in reports of these and related compounds.[b]"	Ref. 23
William A. Tisdale	2023	"Crystals of AgSePh-F_2(2,6) are insoluble and retain their luminescence properties even when submerged in polar organic solvents (N,N′-dimethylacetamide, DMF, acetonitrile, or methanol), a polar aromatic solvent (1,2- dichlorobenzene), or an acidic (pH = 1) or basic solution (pH = 14)."	Ref. 24
Watcharaphol Paritmongkol	2024	"Figure 2c reveals the insolubility and resistance of AgSePy in various common organic solvents"	Ref. 25
J. Nathan Hohman	2024	"MOChas[g] were reported to exhibit both thermal and chemical stability. Therefore, this experiment demonstrates that the initial MOCha 2MMB[i] is not dissolved (in NMP) during the transformation and the 2MMB crystal habits are unchanged from the starting material. This implies that dissolution of the initial MOCha is not a critical step to achieve a complete transformation."	Ref. 26

[a]Transition metal mercaptides of 1,4-dimercaptobenzene, [b]silver alkylthiolates (AgSR), [c]hexanuclear copper(I) complexes of μ_3-pyridine-2-thionate. [d]Homoleptic Copper(I) Thiolates: $(Cu(CH_3S^-))_\infty$, $[(C_6H_5)_4P^+]_2[Cu_5(CH_3S^-)_7] \cdot C_2H_6O_2$, and $[(C_3H_7)_4N^+]_2[Cu_4(CH_3S^-)_6 \cdot CH_4O$, [e]silver phenylselenolate $(Ag(SePh))_\infty$, [f]copper(i) 4-hydroxythiophenolate. [g]metal–organic chalcogenolates (MOChas). [h]gold phenylthiolate $[Au(SPh)]_n$. [i]silver(I) methyl 2-mercaptobenzoate (2MMB).

2. Additional Characterizations on MOC's Solubility and Crystallization Challenges

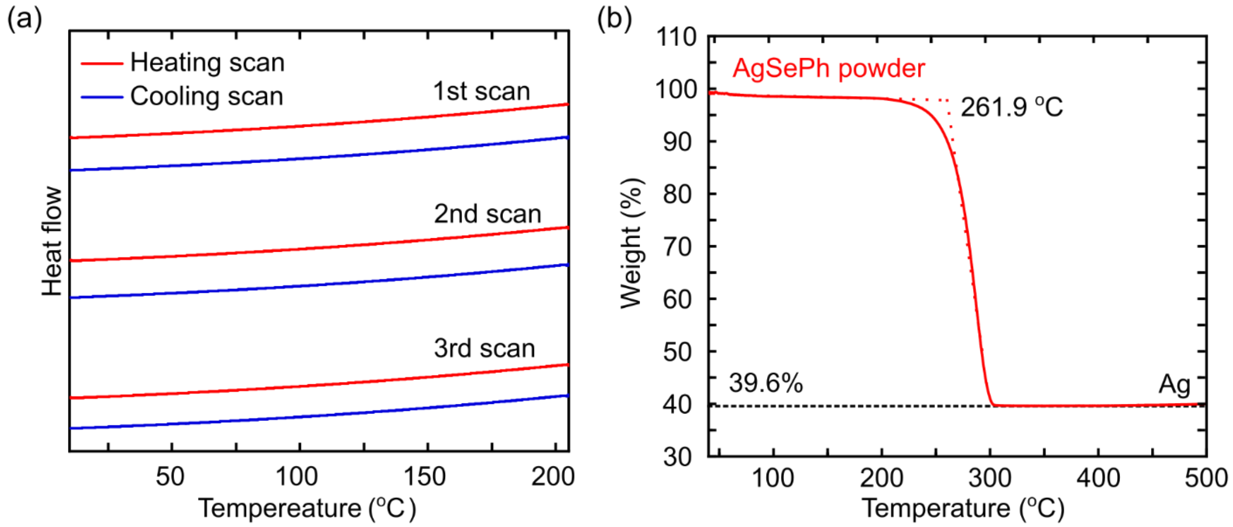

Figure S1. Thermal Properties of AgSePh. a) Differential scanning calorimetry (DSC) and (b) thermogravimetric analysis (TGA) thermograms of AgSePh.

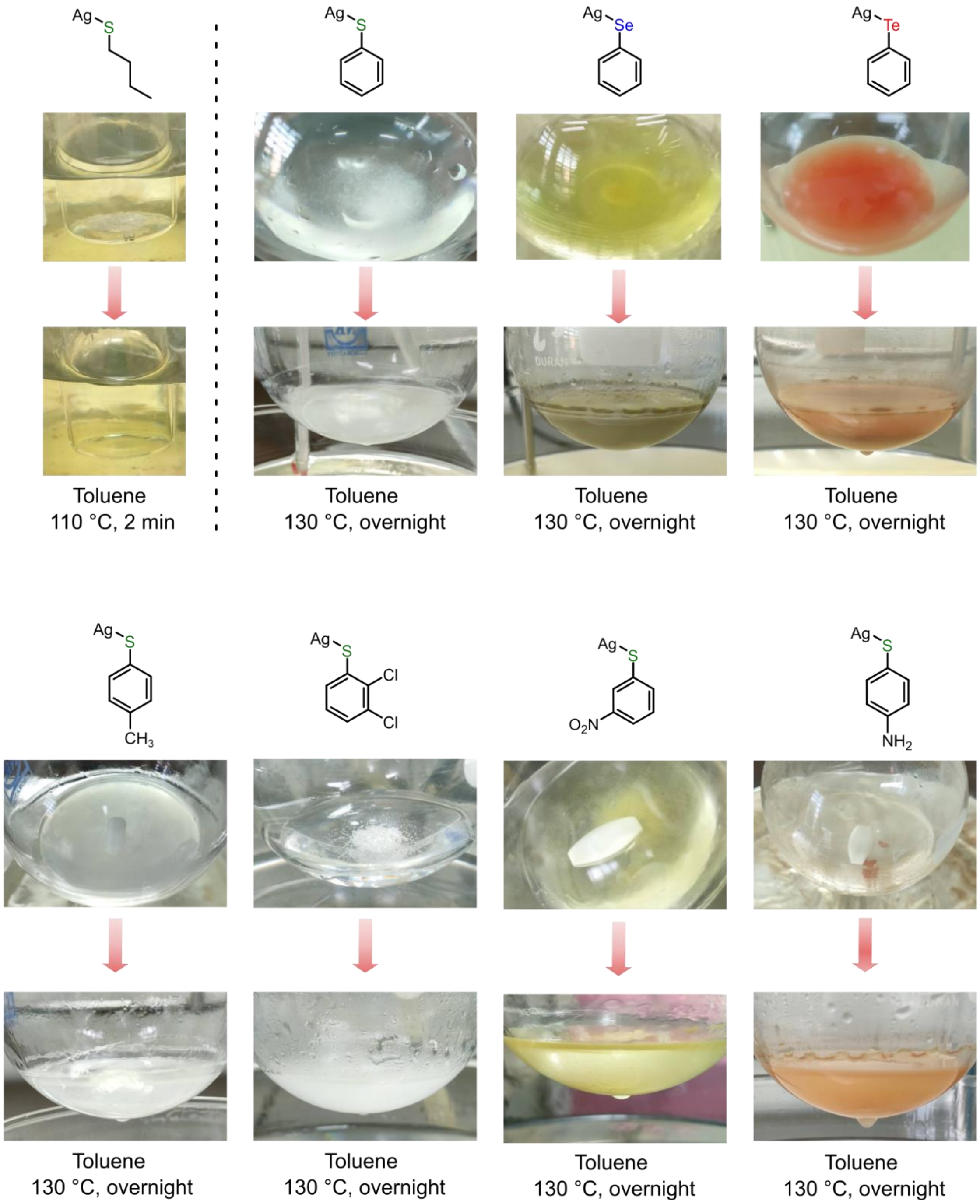

Figure S2. Solubility Test of MOCs in Hot Toluene. Alkyl-bearing AgSBu dissolved in hot toluene, consistent with previous reports.[11, 27] However, phenyl-bearing MOCs, including unfunctionalized-phenyl MOC (AgSPh, AgSePh and AgTePh) and functionalized-phenyl MOC (AgSPh-*p*CH_3, AgSPh-Cl_2(2,3), AgSPh-*m*NO_2 and AgSPh-*p*NH_2), did not dissolve even after refluxing at 130 °C overnight.

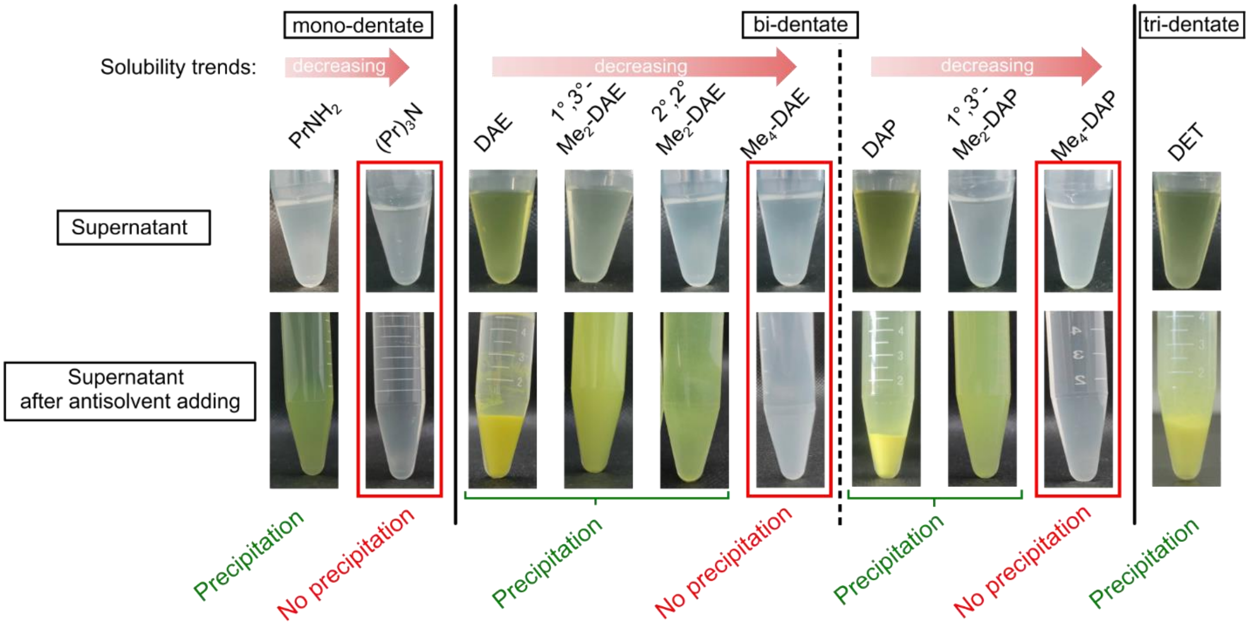

Figure S3. Solubility Test of AgSePh in Amine Solvents. Photographic images of supernatants from saturated AgSePh solutions in various amine solvents and AgSePh precipitates obtained after the addition of excess antisolvents (hexane/IPA). Solubility values were determined by measuring the masses of the precipitates.

3. Additional Characterizations of AgSePh Before and After Recrystallization.

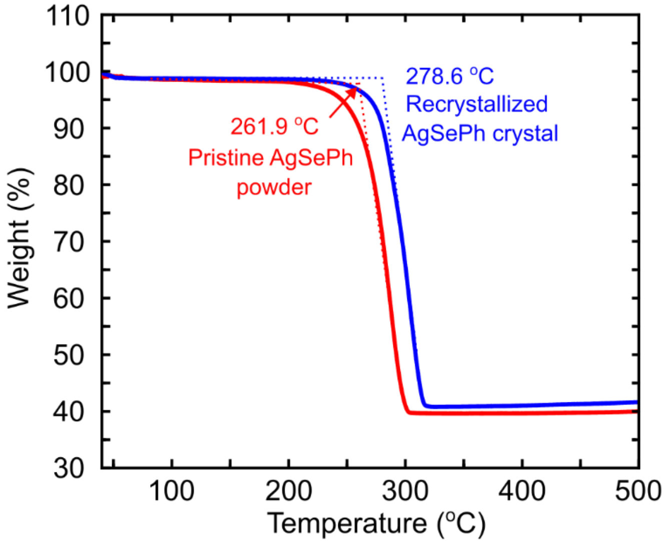

Figure S4. Thermogravimetric Analysis (TGA) of Recrystallized AgSePh. TGA thermograms of recrystallized AgSePh crystals compared with pristine AgSePh powders.

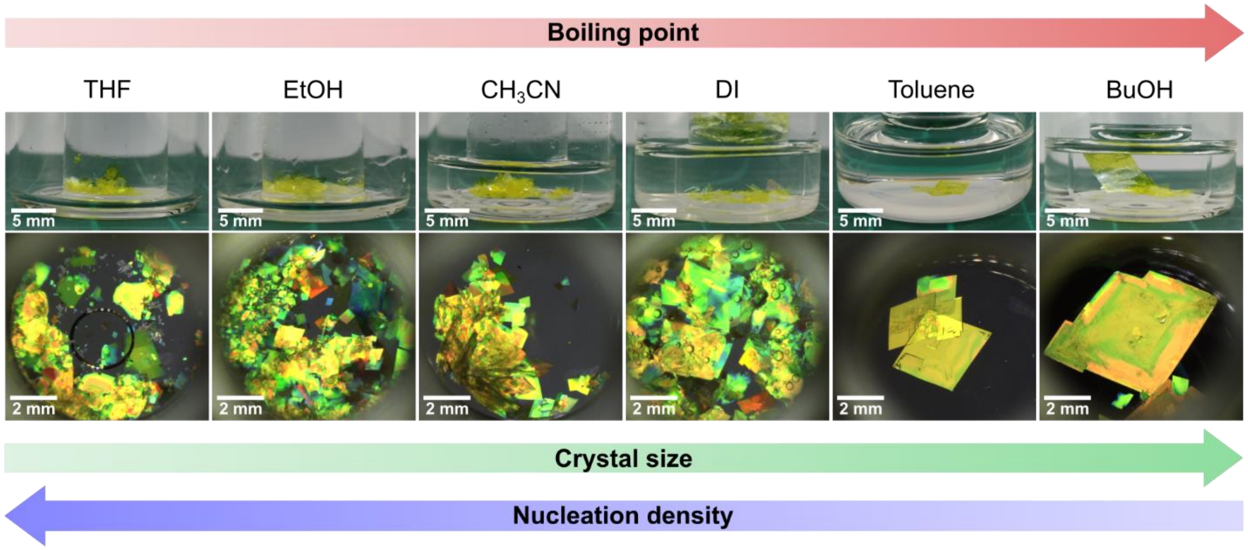

Figure S5. Antisolvent Selection for Recrystallization by Antisolvent Vapor Diffusion. Photographs and optical microscopy images at 10× magnification of recrystallized AgSePh crystals by different antisolvents, including tetrahydrofuran (THF), ethanol (EtOH), acetonitrile (CH_3CN), deionized water (DI), toluene, *n*-butanol (BuOH).

4. Selection of Recrystallization Methods

In our study, we ultimately focused on the antisolvent vapor diffusion method due to safety considerations and practical limitations associated with evaporation and cooling.

- Evaporation: While slow evaporation is a straightforward approach to promote crystallization, it requires handling amine vapors for extended periods. Since amines are volatile, toxic, and can cause skin irritation, this method must be performed in a fume hood, which poses logistical challenges given the limited space in our laboratory.
- Cooling: Cooling-induced crystallization can, in principle, yield large crystals by gradually lowering solubility. However, in our system, amines also act as reducing agents, and elevated temperatures accelerate the formation of metallic Ag as a byproduct. This side reaction complicates the crystallization process and compromises crystal purity.
- Antisolvent diffusion: In contrast, antisolvent vapor diffusion avoids these issues by operating at room temperature and in sealed containers, minimizing exposure to amine vapors and reducing the risk of Ag precipitation. Moreover, by tuning the choice of antisolvent, this method enables better control over nucleation and crystal growth, which allowed us to reproducibly obtain single crystals of AgSePh.

For these reasons, we found the antisolvent method to be the most practical and reliable route for obtaining high-quality single crystals in this work.

5. Antisolvent Selection for Antisolvent Vapor Diffusion Recrystallization

In our experiments, we selected common solvents, such as those shown in Figure 1c, to serve as antisolvents in the recrystallization process. These solvents function as poor solvents for AgSePh, reducing its solubility in amine solutions and thereby promoting nucleation and crystal growth during the antisolvent vapor diffusion process. However, we excluded some certain antisolvents from testing based on three criteria:

(1) Boiling Point: We focused primarily on antisolvents with boiling points lower than that of DAP (140 °C). Solvents with higher boiling points, such as DMF (146 °C) and DMSO (189 °C) caused DAP to evaporate out before the antisolvent diffused in, leading to premature precipitation and poor crystal growth.

(2) Acid–Base Compatibility: Due to the basicity of amines, we avoided antisolvents containing acidic functional groups (e.g., carboxylic acid such as acetic acid), which could protonate the amines and either trigger side reactions or degrade the MOC structures.

(3) Chemical Inertness: To prevent undesired reactions, we excluded nucleophilic and reactive solvents. For example, thiol-containing antisolvents (e.g., propanethiol) could react with the metal center or ligands to yield alternative MOC motifs.

Based on these considerations, we tested six antisolvents with a range of boiling points and polarities (**Figure S5**): THF (66 °C), EtOH (78 °C), CH_3CN (82 °C), DI water (100 °C), toluene (110 °C) and butanol (118 °C). Our findings suggest that polarity alone does not significantly impact crystal size, likely because AgSePh exhibits low solubility in all of tested antisolvents. Instead, the boiling point of the antisolvent appears to play a more critical role: high-boiling antisolvents (e.g., toluene, butanol) yielded larger crystals, whereas lower-boiling counterparts (e.g., THF, ethanol) produced smaller crystals. We attribute this trend to slower vapor diffusion rates of high-boiling solvents, which in turn enable a more gradual decrease in solubility to promote controlled crystal growth.

6. Additional Observation and Structural Characterizations of Functionalized Ag-Based MOC Derivatives

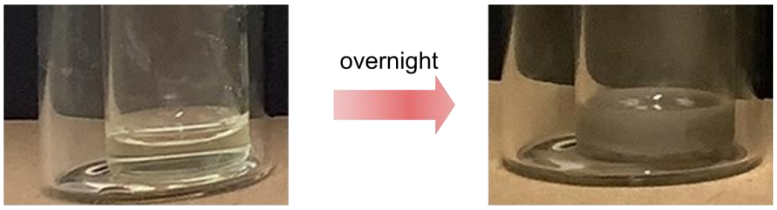

Figure S6. Decomposition of AgSPh-*p*OH. Transformation of AgSPh-*p*OH into black powders in DAP solvent after storing for one night.

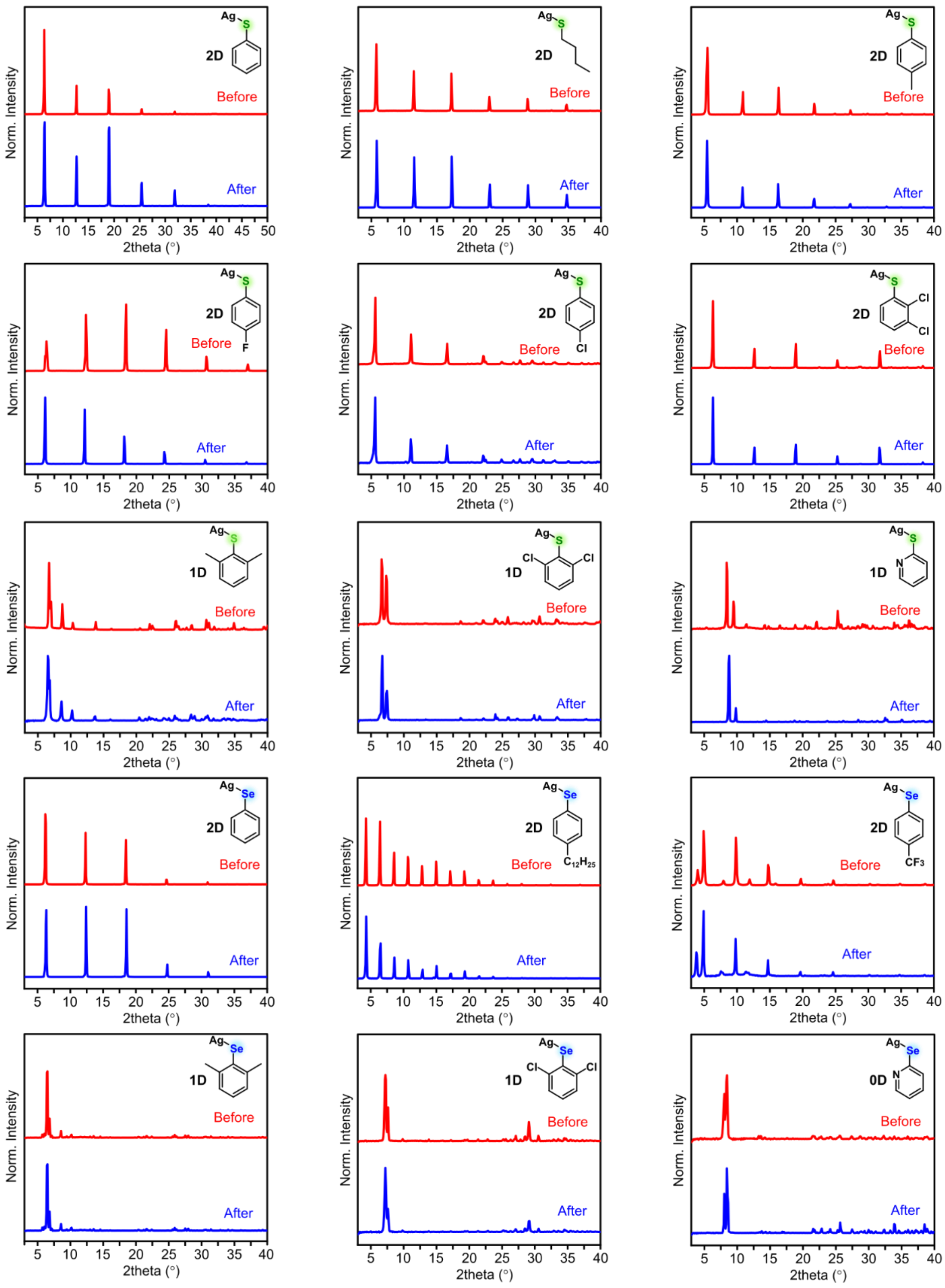

Figure S7. X-ray Diffraction Patterns of MOCs with Unchanged Structures after Recrystallization. PXRD patterns of 15 MOC derivatives before (red) and after (blue) recrystallization, all of which retained their structural integrity.

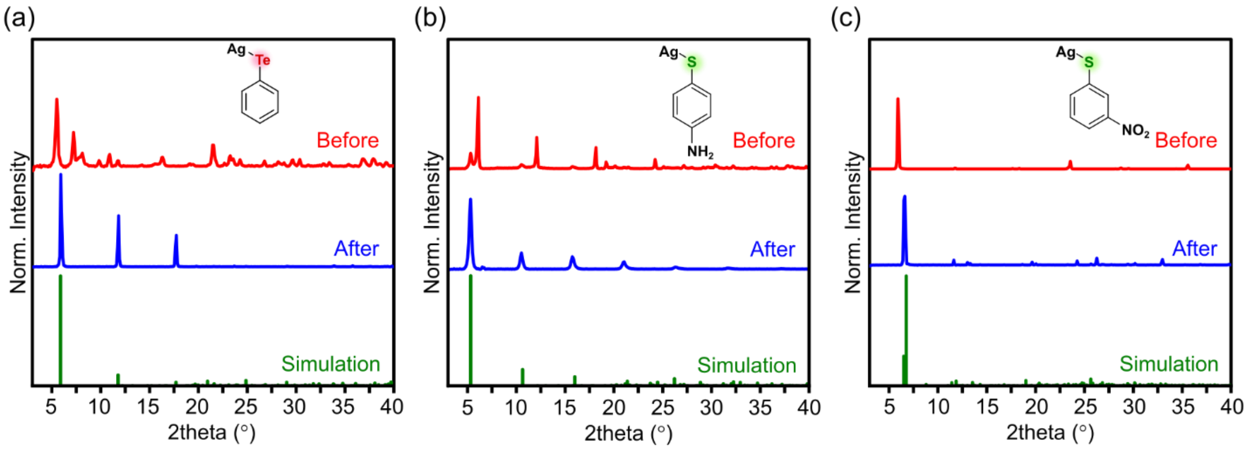

Figure S8. X-ray Diffraction Patterns of MOCs with Structural Transformation after Recrystallization. PXRD patterns of (a) AgTePh, (b) AgSPh-*m*NO_2 and (c) AgSPh-*p*NH_2 before (red) and after (blue) recrystallization from DAP solutions, along with corresponding simulated patterns from structural information obtained in this work.

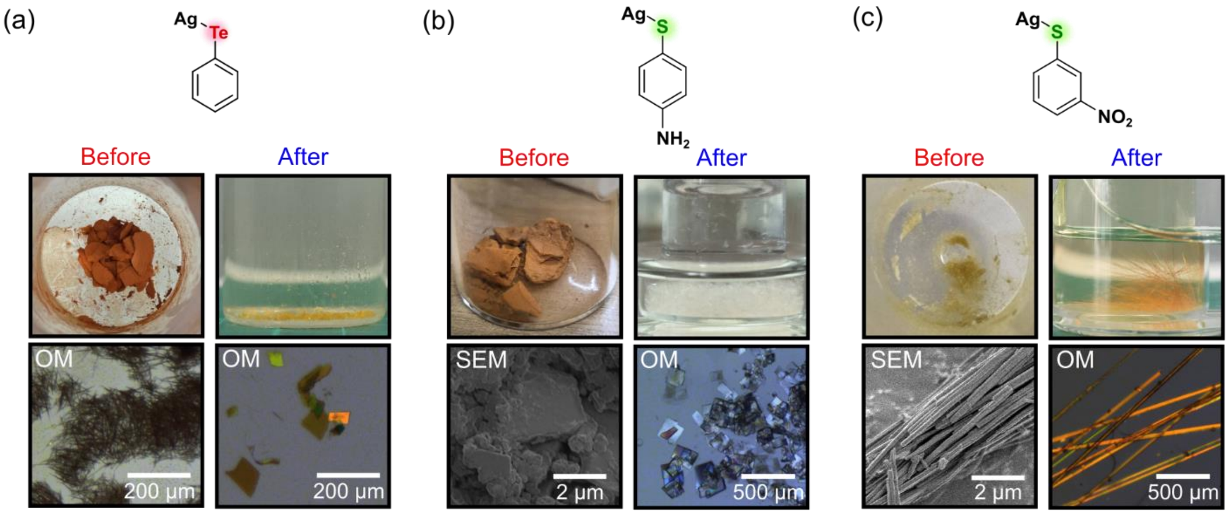

Figure S9. Structural Transformation of AgTePh, AgSPh-*m*NO_2 and AgSPh-*p*NH_2 upon Recrystallization. Photographs as well as optical microscope (OM) and SEM images of (a) AgTePh, (b) AgSPh-*m*NO_2 and (c) AgSPh-*p*NH_2 before and after recrystallization from DAP solutions.

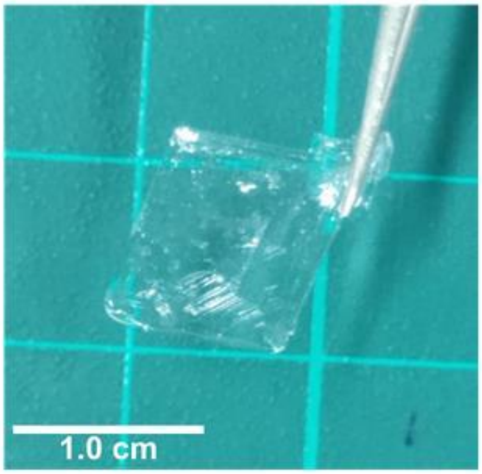

Figure S10. Photographic Image of a Centimeter-Sized AgSPh Crystal. The dimensions of the shown crystal are 1.03 cm x 1.08 cm.

7. Additional Discussion on the Structural and Optical Changes in AgTePh, AgSPh-*p*NH_2 and AgSPh-*m*NO_2

In this study, we observed structural transformations in three representative MOC systems—AgTePh, AgSPh-*p*NH_2, and AgSPh-*m*NO_2—that exhibited altered PXRD patterns upon recrystallization (**Figure S8**), consistent with significant phase changes.

For **AgTePh**, recrystallization led to a clear phase evolution from red, 1D fibrous intermediates to brown, plate-like 2D crystals (**Figure S9a**). This transformation is likely driven by the release or rearrangement of trapped amines, followed by reorganization into a more thermodynamically stable 2D layered structure, consistent with previous reports of amine-assisted AgTePh synthesis.[28] The associated shift in photoluminescence (PL) – from emission above 750 nm in the 1D fibers to a broad ~600 nm emission in the 2D plates (**Figure S15a**) –further supports a change in electronic structure arising from differences in dimensionality and packing.

For **AgSPh-*p*NH$_2$**, recrystallization converted a brown-orange powder with sub-micron plate-like morphology and dual-phase PXRD pattern into white, plate-like crystals exhibiting a single-phase PXRD pattern (**Figure S8b and S9b**), matching the minor phase in the original sample. SCXRD confirmed that the recrystallized product adopts a 2D layered structure with intercalated H_2O molecules stabilized by hydrogen bonding **(Figure 3, Table 2 and Figure S12a)**. Due to the similarity of the major and minor phase PXRD patterns, we therefore propose that the pre-recrystallized sample also possessed a 2D layered structure but as a mixture of hydrated and non-hydrated phases. The brown-orange coloration (**Figure S9b**) and shifted absorption spectrum (**Figure S15b**) are likely attributable to impurities or byproducts formed during synthesis. These results highlight the dissolution–recrystallization approach as an effective method for producing phase-pure, well-defined MOCs that are otherwise difficult to isolate through direct synthesis.

For **AgSPh-*m*NO$_2$**, recrystallization transformed a pale-yellow powder with 1D-like morphology into orange, 1D needle-like crystals (**Figure S9c**), indicating a structural rearrangement. SCXRD revealed that recrystallization led to co-crystallization with DAP through Ag–N coordination along the polymeric chain **(Figure 3, Table 2 and Figure S12b)**. Although the PXRD patterns before and after recrystallization indicated broadly similar 1D frameworks, subtle peak shifts (**Figure S8c**) suggest changes arising from the presence or absence of coordinated diamine. These structural differences were accompanied by pronounced shifts in both UV–vis absorption and PL spectra (**Figure S15c**), confirming that the altered coordination environment from co-crystallization strongly modifies the optical properties.

8. Crystallographic Information

Table S2. Crystal data and structure refinement for all MOCs

	**MOC derivatives**		
	**AgSPh**	**AgSePh**	**AgTePh**
CCDC	2413037	2407479	2412938
Empirical formula	$C_{12}H_{10}S_2Ag_2$	$C_{12}H_{10}Ag_2Se_2$	$C_{12}H_{10}Ag_2Te_2$
Formula weight	434.06	527.86	625.14
Temperature/K	150	150	100
Crystal system	monoclinic	monoclinic	monoclinic
Space group	$P2_1/c$	$P2_1/c$	$\mathrm{P2_1/c}$
a/Å	7.3400(2)	5.8543(3)	5.8397(5)
b/Å	5.8091(2)	7.2849(3)	7.4622(6)
c/Å	28.0817(10)	29.1004(13)	30.105(3)
α/°	90	90	90
β/°	94.0700(10)	95.831(2)	92.99
γ/°	90	90	90
Volume/$Å^3$	1194.35(7)	1234.65(10)	1310.10(19)
Z	4	4	4
$\rho_{calc}g/cm^3$	2.414	2.840	3.169
μ/mm^{-1}	3.593	9.034	7.323
F(000)	832	976.0	1120.0
Crystal size/mm^3	0.07 × 0.041 × 0.009	0.124 × 0.101 × 0.01	0.249 × 0.057 × 0.008
Radiation	MoKα (λ = 0.71073)	MoKα (λ = 0.71073)	MoKα (λ = 0.71073)
2Θ range for data collection/°	5.564 to 52.43	5.628 to 55.824	4.064 to 52.044
Index ranges	-9 ≤ h ≤ 9, -7 ≤ k ≤ 7, -34 ≤ l ≤ 34	-7 ≤ h ≤ 7, -9 ≤ k ≤ 8, -38 ≤ l ≤ 38	-7 ≤ h ≤ 7, -8 ≤ k ≤ 9, -37 ≤ l ≤ 37
Reflections collected	29905	40606	14137
Independent reflections	2410 [R_{int} = 0.0496, R_{sigma} = 0.0195]	2941 [R_{int} = 0.0344, R_{sigma} = 0.0156]	2583 [R_{int} = 0.0536, R_{sigma} = 0.0400]
Data/restraints/parameters	2410/0/145	2941/0/145	2583/0/146
Goodness-of-fit on F^2	1.114	1.116	1.154
Final R indexes [I>=2σ (I)]	R_1 = 0.0453, wR_2 = 0.0854	R_1 = 0.0288, wR_2 = 0.0572	R_1 = 0.0424, wR_2 = 0.0984
Final R indexes [all data]	R_1 = 0.0703, wR_2 = 0.1067	R_1 = 0.0393, wR_2 = 0.0665	R_1 = 0.1029, wR_2 = 0.1434
Largest diff. peak/hole / e $Å^{-3}$	1.71/-1.96	0.97/-1.47	1.97/-1.79

Table S2. Crystal data and structure refinement for all MOCs (cont.)

	MOC derivatives		
	**AgSBu**	**AgSPh-*p*CH3**	**AgSPh-*p*F**
CCDC	2413041	2412937	2412916
Empirical formula	C_4H_9AgS	C_7H_7AgS	C_6H_4AgFS
Formula weight	197.04	231.06	235.02
Temperature/K	100	100	150
Crystal system	monoclinic	orthorhombic	orthorhombic
Space group	$P2_1/c$	*Pbca*	*Pbca*
a/Å	4.47440(10)	7.4038(3)	5.8834(3)
b/Å	4.25740(10)	5.7623(2)	7.2298(4)
c/Å	30.9640(10)	32.3538(13)	29.2863(15)
α/°	90	90	90
β/°	92.4100(10)	90	90
γ/°	90	90	90
Volume/$Å^3$	589.32(3)	1380.31(9)	1245.72(11)
Z	4	8	8
$\rho_{calc}g/cm^3$	2.221	2.224	2.506
μ/mm^{-1}	3.627	3.116	3.478
F(000)	384.0	896.0	896.0
Crystal size/mm^3	0.179 × 0.063 × 0.01	0.073 × 0.062 × 0.008	0.18 × 0.166 × 0.01
Radiation	MoKα (λ = 0.71073)	MoKα (λ = 0.71073)	MoKα (λ = 0.71073)
2Θ range for data collection/°	5.268 to 52.752	5.036 to 50.05	5.564 to 54.93
Index ranges	$-5 \le h \le 5$, $-5 \le k \le 5$, $-38 \le l \le 38$	$-8 \le h \le 8$, $-6 \le k \le 6$, $-38 \le l \le 38$	$-7 \le h \le 7$, $-9 \le k \le 9$, $-37 \le l \le 37$
Reflections collected	15294	22365	24128
Independent reflections	1205 [R_{int} = 0.0191, R_{sigma} = 0.0090]	1218 [R_{int} = 0.0576, R_{sigma} = 0.0174]	1423 [R_{int} = 0.0443, R_{sigma} = 0.0159]
Data/restraints/parameters	1205/0/59	1218/0/83	1423/0/82
Goodness-of-fit on F^2	1.211	1.122	1.089
Final R indexes [I>=2σ (I)]	R_1 = 0.0148, wR_2 = 0.0316	R_1 = 0.0535, wR_2 = 0.0963	R_1 = 0.0499, wR_2 = 0.1047
Final R indexes [all data]	R_1 = 0.0175, wR_2 = 0.0338	R_1 = 0.0782, wR_2 = 0.1158	R_1 = 0.0669, wR_2 = 0.1206
Largest diff. peak/hole / e $Å^{-3}$	0.43/-0.44	1.89/-1.48	2.00/-1.61

Table S2. Crystal data and structure refinement for all MOCs (cont.)

	MOC derivatives		
	**AgSPh-Cl2(2,3)**	**AgSPh-Cl2(2,6)**	**AgSPh-Me2(2,6)**
CCDC	2412923	2412941	2412942
Empirical formula	$C_6H_3AgCl_2S$	$C_{12}H_6S_2Cl_4Ag_2$	$C_{16}H_{18}Ag_2S_2$
Formula weight	285.91	571.83	490.16
Temperature/K	100	100	100
Crystal system	monoclinic	orthorhombic	monoclinic
Space group	$P2_1$	$P2_12_12$	$P2/c$
a/Å	4.0578(14)	13.5008(11)	13.1733(4)
b/Å	6.481(2)	26.567(2)	4.36580(10)
c/Å	14.058(4)	4.1906(4)	27.3233(8)
α/°	90	90	90
β/°	91.665(15)	90	99.4380(10)
γ/°	90	90	90
Volume/Å^3	369.6(2)	1503.0(2)	1550.15(7)
Z	2	4	4
ρ_{calc}g/cm^3	2.569	2.527	2.100
μ/mm^{-1}	3.636	3.576	2.781
F(000)	272.0	1088.0	960.0
Crystal size/mm^3	0.283 × 0.047 × 0.01	0.263 × 0.029 × 0.01	0.49 × 0.025 × 0.008
Radiation	MoKα (λ = 0.71073)	MoKα (λ = 0.71073)	MoKα (λ = 0.71073)
2Θ range for data collection/°	5.798 to 54.324	4.302 to 54.958	4.698 to 55.04
Index ranges	$-5 \le h \le 5, -8 \le k \le 8, -18 \le l \le 18$	$-17 \le h \le 17, -34 \le k \le 34, -4 \le l \le 5$	$-17 \le h \le 17, -5 \le k \le 5, -35 \le l \le 35$
Reflections collected	7980	16999	36589
Independent reflections	1579 [R_{int} = 0.0342, R_{sigma} = 0.0268]	3452 [R_{int} = 0.0440, R_{sigma} = 0.0336]	3574 [R_{int} = 0.0377, R_{sigma} = 0.0166]
Data/restraints/parameters	1579/1/91	3452/0/182	3574/0/185
Goodness-of-fit on F^2	1.120	1.153	1.193
Final R indexes [I>=2σ (I)]	R_1 = 0.0360, wR_2 = 0.0730	R_1 = 0.0388, wR_2 = 0.0823	R_1 = 0.0229, wR_2 = 0.0499
Final R indexes [all data]	R_1 = 0.0397, wR_2 = 0.0770	R_1 = 0.0516, wR_2 = 0.0946	R_1 = 0.0317, wR_2 = 0.0581
Largest diff. peak/hole / eÅ^{-3}	1.06/-1.27	0.95/-1.22	1.11/-0.74
Flack parameter	0.02(2)	0.004(19)	-

Table S2. Crystal data and structure refinement for all MOCs (cont.)

	MOC derivatives		
	**AgSPy**	**[AgSPh-*p*NH$_2$]$_2$ ·1H$_2$O**	**[AgSPh-*m*NO$_2$]$_4$·1DAP**
CCDC	2412929	2412913	2412943
Empirical formula	$C_{15}H_{12}Ag_3N_3S_3$	$C_{12}H_{14}Ag_2N_2OS_2$	$C_{27}H_{26}Ag_4N_6O_8S_4$
Formula weight	654.07	482.11	1122.26
Temperature/K	100	100	100
Crystal system	monoclinic	monoclinic	orthorhombic
Space group	$P2_1/c$	$P2_1/c$	$P2_12_12_1$
a/Å	7.7484(4)	5.6102(4)	8.3938(4)
b/Å	10.4578(6)	33.255(2)	14.9176(8)
c/Å	20.9713(11)	7.4570(5)	27.1971(15)
α/°	90	90	90
β/°	90.339(2)	91.485(3)	90
γ/°	90	90	90
Volume/Å^3	1699.30(16)	1390.75(17)	3405.5(3)
Z	4	4	4
ρ_{calc}g/cm^3	2.557	2.303	2.189
μ/mm^{-1}	3.793	3.107	2.569
F(000)	1248.0	936.0	2184.0
Crystal size/mm^3	0.642 × 0.108 × 0.011	0.11 × 0.032 × 0.008	0.14 × 0.025 × 0.014
Radiation	MoKα (λ = 0.71073)	MoKα (λ = 0.71073)	MoKα (λ = 0.71073)
2Θ range for data collection/°	4.352 to 62.344	4.9 to 50.054	4.052 to 54.998
Index ranges	-11 ≤ h ≤ 9, -15 ≤ k ≤ 15, -30 ≤ l ≤ 30	-6 ≤ h ≤ 6, -39 ≤ k ≤ 39, -8 ≤ l ≤ 8	-9 ≤ h ≤ 10, -19 ≤ k ≤ 19, -35 ≤ l ≤ 35
Reflections collected	76265	14422	89526
Independent reflections	5307 [R_{int} = 0.0283, R_{sigma} = 0.0150]	2419 [R_{int} = 0.0445, R_{sigma} = 0.0304]	7805 [R_{int} = 0.0599, R_{sigma} = 0.0258]
Data/restraints/parameters	5307/0/217	2419/0/181	7805/0/442
Goodness-of-fit on F^2	1.102	1.103	1.240
Final R indexes [I>=2σ (I)]	R_1 = 0.0174, wR_2 = 0.0360	R_1 = 0.0558, wR_2 = 0.1021	R_1 = 0.0227, wR_2 = 0.0482
Final R indexes [all data]	R_1 = 0.0254, wR_2 = 0.0401	R_1 = 0.0809, wR_2 = 0.1208	R_1 = 0.0311, wR_2 = 0.0553
Largest diff. peak/hole / e Å^{-3}	1.60/-1.70	1.88/-1.98	0.71/-0.69
Flack parameter	-	-	0.024(11)

Table S2. Crystal data and structure refinement for all MOCs (cont.)

	MOC derivatives		
	**AgSePh-Me2(2,6)**	**AgSePh-Cl2(2,6)**	**AgSePy**
CCDC	2412918	2412930	2412932
Empirical formula	$C_{16}H_{18}Se_2Ag_2$	$C_{12}H_6Cl_4Se_2Ag_2$	$C_{30}H_{24}Ag_6N_6Se_6$
Formula weight	583.96	665.63	1589.53
Temperature/K	100	100	100
Crystal system	triclinic	monoclinic	triclinic
Space group	*P*-1	$P2_1/c$	*P*-1
a/Å	4.6660(3)	4.6495(9)	7.0587(2)
b/Å	12.2421(9)	13.232(3)	12.0014(4)
c/Å	13.5623(9)	24.381(5)	12.6430(4)
α/°	87.676(2)	90	114.4540(10)
β/°	88.551(2)	90.557(7)	102.0070(10)
γ/°	89.884(3)	90	99.7030(10)
Volume/Å^3	773.82(9)	1500.0(5)	913.80(5)
Z	2	4	1
ρ_{calc}g/cm^3	2.506	2.948	2.888
μ/mm^{-1}	7.220	8.159	9.159
F(000)	552.0	1232.0	732.0
Crystal size/mm^3	0.147 × 0.03 × 0.01	0.115 × 0.04 × 0.01	0.167 × 0.063 × 0.04
Radiation	MoKα (λ = 0.71073)	MoKα (λ = 0.71073)	MoKα (λ = 0.71073)
2Θ range for data collection/°	4.396 to 55.06	4.544 to 51.364	3.938 to 60.07
Index ranges	-5 ≤ h ≤ 6, -15 ≤ k ≤ 15, -17 ≤ l ≤ 17	-5 ≤ h ≤ 5, -16 ≤ k ≤ 16, -29 ≤ l ≤ 29	-9 ≤ h ≤ 9, -16 ≤ k ≤ 16, -17 ≤ l ≤ 17
Reflections collected	25111	29269	29423
Independent reflections	3499 [R_{int} = 0.0516, R_{sigma} = 0.0290]	2837 [R_{int} = 0.0597, R_{sigma} = 0.0273]	5165 [R_{int} = 0.0224, R_{sigma} = 0.0164]
Data/restraints/parameters	3499/0/185	2837/0/176	5165/0/218
Goodness-of-fit on F^2	1.167	1.149	1.167
Final R indexes [I>=2σ (I)]	R_1 = 0.0315, wR_2 = 0.0646	R_1 = 0.0696, wR_2 = 0.1318	R_1 = 0.0172, wR_2 = 0.0404
Final R indexes [all data]	R_1 = 0.0469, wR_2 = 0.0767	R_1 = 0.0924, wR_2 = 0.1512	R_1 = 0.0204, wR_2 = 0.0432
Largest diff. peak/hole / e Å^{-3}	1.44/-1.41	1.99/-1.54	0.74/-0.84

Table S3. Summary of crystal structures and comparison to previous reports

MOCs	Structure reported in this work			Structure reported in literature			
	Crystal system	Space group	Technique	Crystal system	Space group	Technique	Reference
AgSPh	monoclinic	$P2_1/c$	SCXRD	monoclinic	Cc	smSFX	Ref. [29]
				monoclinic	$P2_1$	SCXRD	Ref. [28]
AgSePh	monoclinic	$P2_1/c$	SCXRD	monoclinic	$P2_1/c$	SCXRD	Ref. [30]
				monoclinic	$C2/c$	SCXRD	Ref. 13
				monoclinic	$C2/c$	smSFX	Ref. [29]
AgTePh	monoclinic	$P2_1/c$	SCXRD	monoclinic	$P2_1/c$	SCXRD	Ref. [28]
				monoclinic	$C2/c$	smSFX	Ref. [29]
AgSBu	monoclinic	$P2_1/c$	SCXRD	monoclinic	$P2_1/n$	smSFX	Ref. 23
AgSPh-*p*F	orthorhombic	*Pbca*	SCXRD	orthorhombic	$P2_12_12_1$	Le Bail refinement of PXRD pattern	Ref. [31]
				orthorhombic	*Cmce*	smSFX	Ref. [32]
AgSPy	monoclinic	$P2_1/c$	SCXRD	monoclinic	$P2_1/c$	SCXRD	Ref. [33]
AgSePy	triclinic	*P*-1	SCXRD	triclinic	*P*-1	SCXRD	Ref. 25
AgSPh-*p*CH$_3$	orthorhombic	*Pbca*	SCXRD	New report in this work			
AgSPh-Cl$_2$(2,3)	monoclinic	$P2_1$	SCXRD	New report in this work			
AgSPh-Cl$_2$(2,6)	orthorhombic	$P2_12_12$	SCXRD	New report in this work			
AgSPh-Me$_2$(2,6)	monoclinic	$P2/c$	SCXRD	New report in this work			
[AgSPh-*m*NO$_2$]$_4$ ·1DAP	orthorhombic	$P2_12_12_1$	SCXRD	New report in this work			
[AgSPh-*p*NH$_2$]$_2$ ·1H$_2$O	monoclinic	$P2_1/c$	SCXRD	New report in this work			
AgSePh-Me$_2$(2,6)	triclinic	*P*-1	SCXRD	New report in this work			
AgSePh-Cl$_2$(2,6)	monoclinic	$P2_1/c$	SCXRD	New report in this work			

9. Additional Structural Figures.

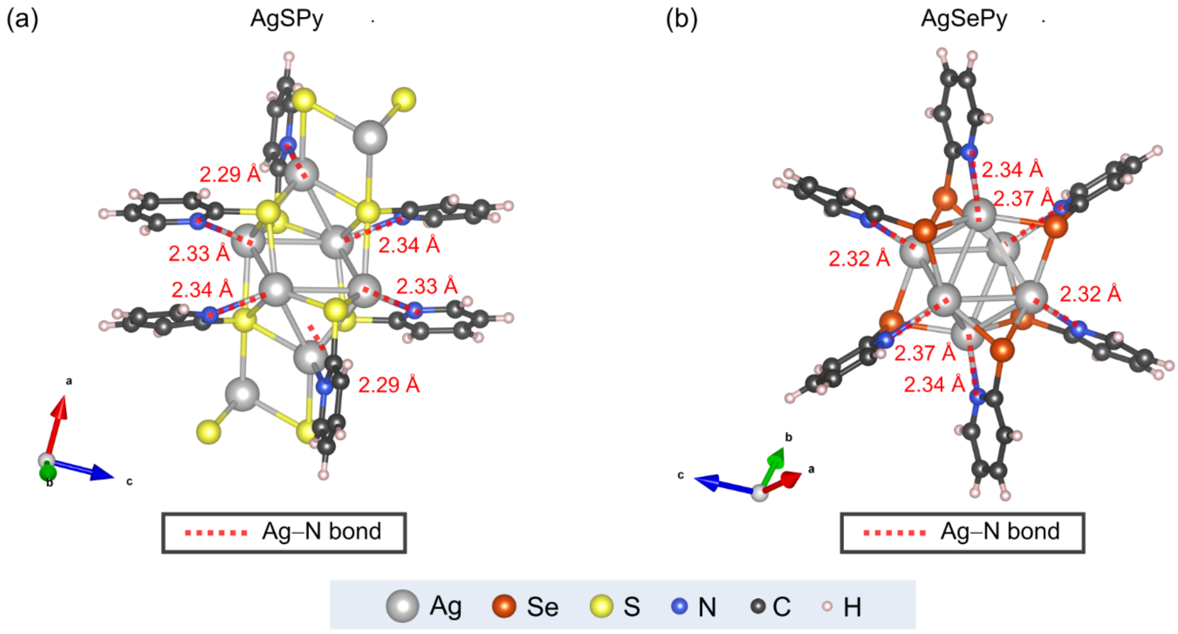

Figure S11. Intramolecular Ag-N Bonds in AgSPy and AgSePy. Structures of (a) 1D AgSPy and (b) 0D AgSePy, showing Ag-N separations of 2.29 Å to 2.37 Å, indicating bonding interactions.

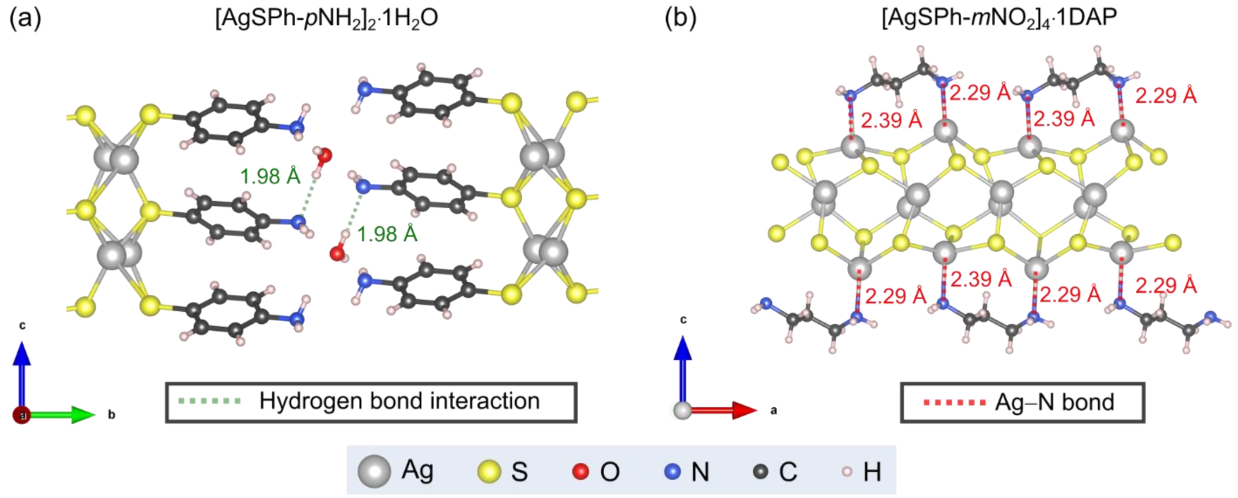

Figure S12. Bonding Interactions Between Co-Crystallized Solvents and MOCs. (a) H-bonding interactions between H_2O and 2D AgSPh-*p*NH_2 in co-crystallized [AgSPh-*p*NH_2]$_2$·H_2O (b) Ag-N bonding interactions between DAP and 1D AgSPh-*m*NO_2 in co-crystallized [AgSPh-*m*NO_2]$_4$·1DAP (nitrophenyl rings omitted for clarity.)

10. Generalization of the Dissolution Approach to Cu-Based MOCs

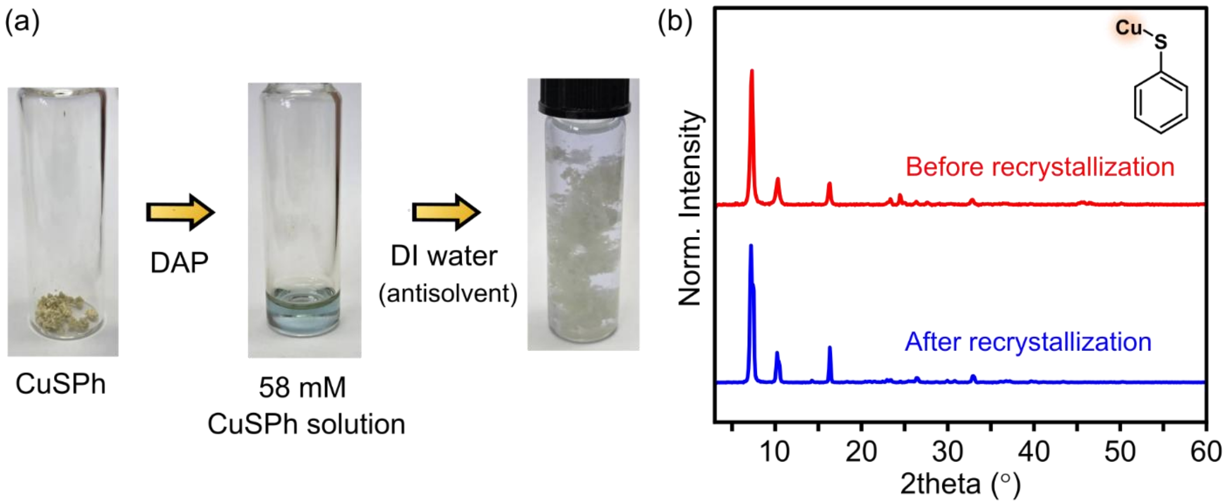

Figure S13. **Dissolution and Recrystallization of Cu-based MOC.** a) Optical images showing the dissolution of CuSPh in DAP and its recrystallization after using DI water as antisolvent. b) PXRD patterns of CuSPh before and after recrystallization.

11. Optical Characterizations of Functionalized Ag-Based MOC Derivatives

Table S4. Summary of optical properties of MOC derivatives before and after recrystallization.

MOC	condition	λ_{abs} (nm)	λ_{em} (nm)	FWHM (nm)	PLQY (%)	Lifetime	
						τ_1 (ns) / Rel%	τ_2 (ns) / Rel%
MOC without structural change after recrystallization							
2D Ag**S**Ph	before	362	404	55	N.D.	N.D.	N.D.
	After	359	406	65	N.D.	N.D.	N.D.
2D Ag**S**Bu	before	298	435	93	N.D.	N.D.	N.D.
	After	306	437	71	N.D.	N.D.	N.D.
2D Ag**S**Ph-*p*CH_3	before	341	415	80	N.D.	N.D.	N.D.
	After	347	414	64	N.D.	N.D.	N.D.
2D Ag**S**Ph-*p*F	before	375	417	61	N.D.	N.D.	N.D.
	After	381	413	65	N.D.	N.D.	N.D.
2D Ag**S**Ph-*p*Cl	before	340	431	90	N.D.	N.D.	N.D.
	After	344	434	82	N.D.	N.D.	N.D.
2D Ag**S**Ph-Cl_2(2,3)	before	271	470	166	N.D.	N.D.	N.D.
	After	287	470	178	N.D.	N.D.	N.D.
1D Ag**S**Ph-Me_2(2,6)	before	473	778	171	8.13	128.6 (9.8%)	413.8 (90.2%)
	After	468	775	173	7.14	105.3 (9.4%)	407.6 (90.6%)
1D Ag**S**Ph-Cl_2(2,6)	before	409	635	184	33.78	174.9 (8.2%)	1236.0 (91.8%)
	After	411	639	173	34.03	268.4 (9.9%)	1372.6 (90.1%)
1D Ag**S**Py	before	351	514	121	1.91	2.9 (9.0%)	4868.3 (91.0%)
	After	348	505	112	2.74	5.1 (18.1 %)	4552.4 (81.9%)
2D Ag**Se**Ph	before	431,449	468	28	0.12	~0.18 (100%)	-
	After	433,448	464	16	0.16	~0.21 (100%)	-
2D Ag**Se**Ph-*p*$C_{12}H_{25}$	before	443,463	480	25	0.11	~0.2/	~2.1/

**MOC**	condition	λ_{abs} (nm)	λ_{em} (nm)	FWHM (nm)	PLQY (%)	Lifetime	
						τ1 (ns) / Rel%	τ2 (ns) / Rel%
						(77.3%)	(21.7%)
	After	445,466	482	20	0.24	~0.2/ (77.8%)	~2.1/ (22.2%)
2D Ag**Se**Ph-*p*CF_3	before	418	466	23	0.09	~0.5 (88.2%)	~4.97 (11.8%)
	After	421	460	25	0.15	~2.2 (74.9%)	~7.12 (25.1%)
1D Ag**Se**Ph-Me_2(2,6)	before	461,550	654	108	12.63	105.6 (100%)	-
	After	449,551	654	96	16.26	83.3 (100%)	-
1D Ag**Se**Ph-Cl_2(2,6)	before	408, 493	606	130	8.77	12.3 (46.5%)	53.4 (53.5%)
	After	397,487	599	108	10.86	19.6 (40.1%)	79.3 (59.9%)
0D Ag**Se**Py	before	378	629	120	49.87	665.3 (10.8%)	3274.9 (89.16%)
	After	381	627	120	54.01	806.1 (5.4%)	3286.34 (94.6%)
MOC with structural change after recrystallization							
AgSPh-*p*NH_2	before	326	437	93	N.D.	N.D.	N.D.
	After	320	438	89	N.D.	N.D.	N.D.
AgSPh-*m*NO_2	before	291	430	76	N.D.	N.D.	N.D.
	After	322	455	123	N.D.	N.D.	N.D.
AgTePh	before	474,545	>800	N.D.	0.31	N.D.	N.D.
	After	414,479	597	106	0.52	N.D.	N.D.

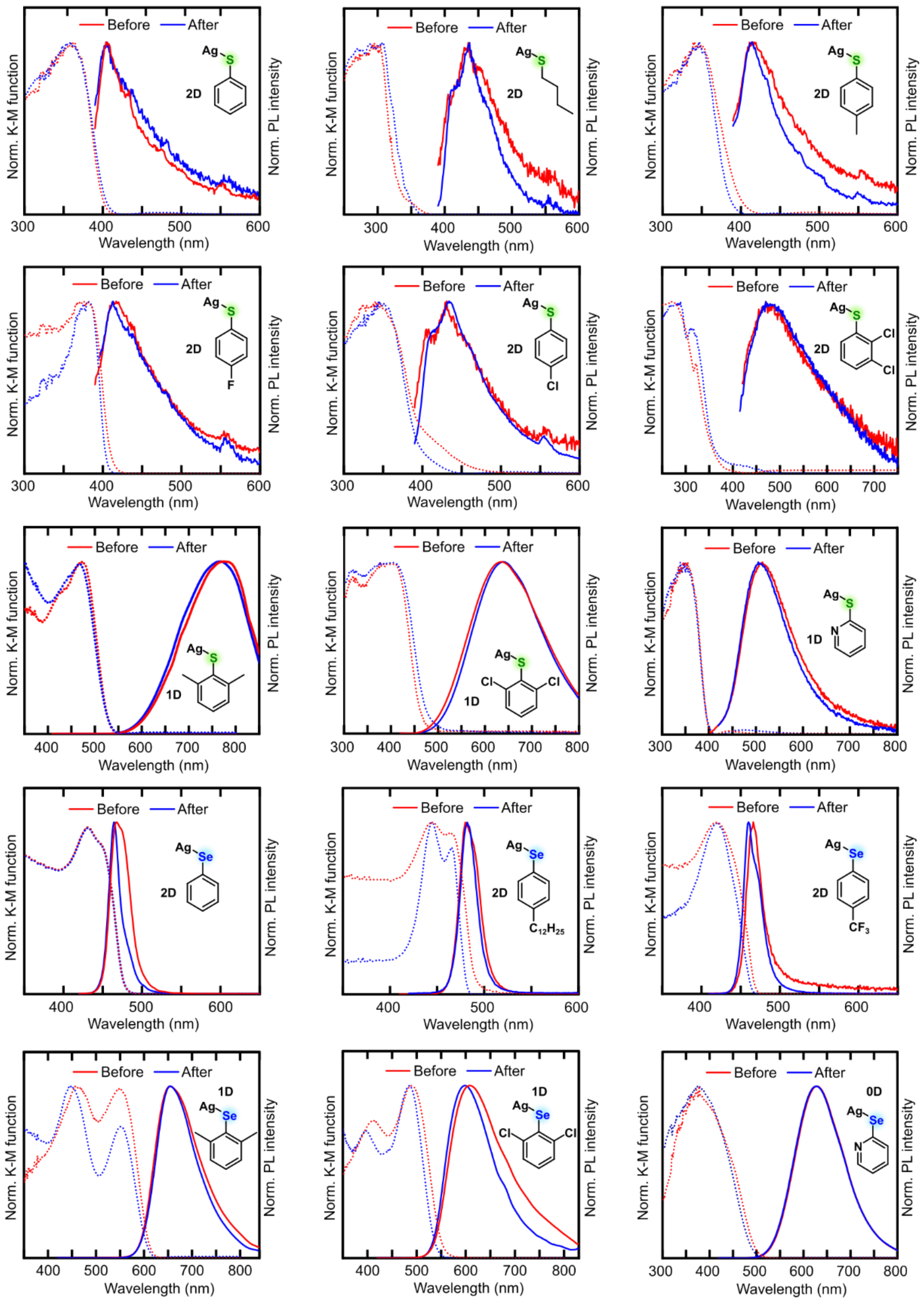

Figure S14. Optical Properties of MOCs with Unchanged Structures after Recrystallization. UV-Vis absorption (dotted) and photoluminescence (solid) spectra of MOC derivatives before (red) and after (blue) recrystallization.

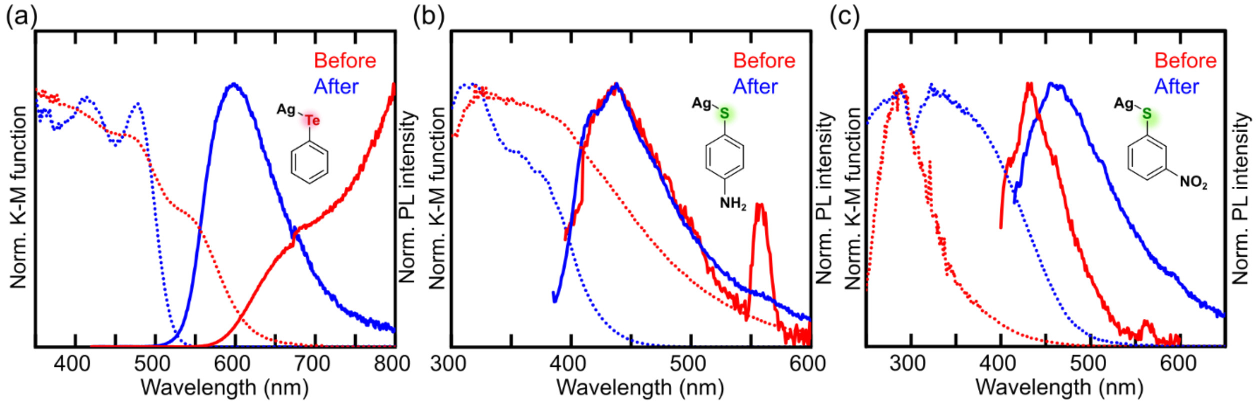

Figure S15. Optical Properties of MOCs with Structural Transformation after Recrystallization. UV-Vis absorption and photoluminescence (PL) spectra of (a) AgTePh, (b) AgSPh-*m*NO_2 and (c) AgSPh-*p*NH_2 before and after recrystallization from DAP solutions.

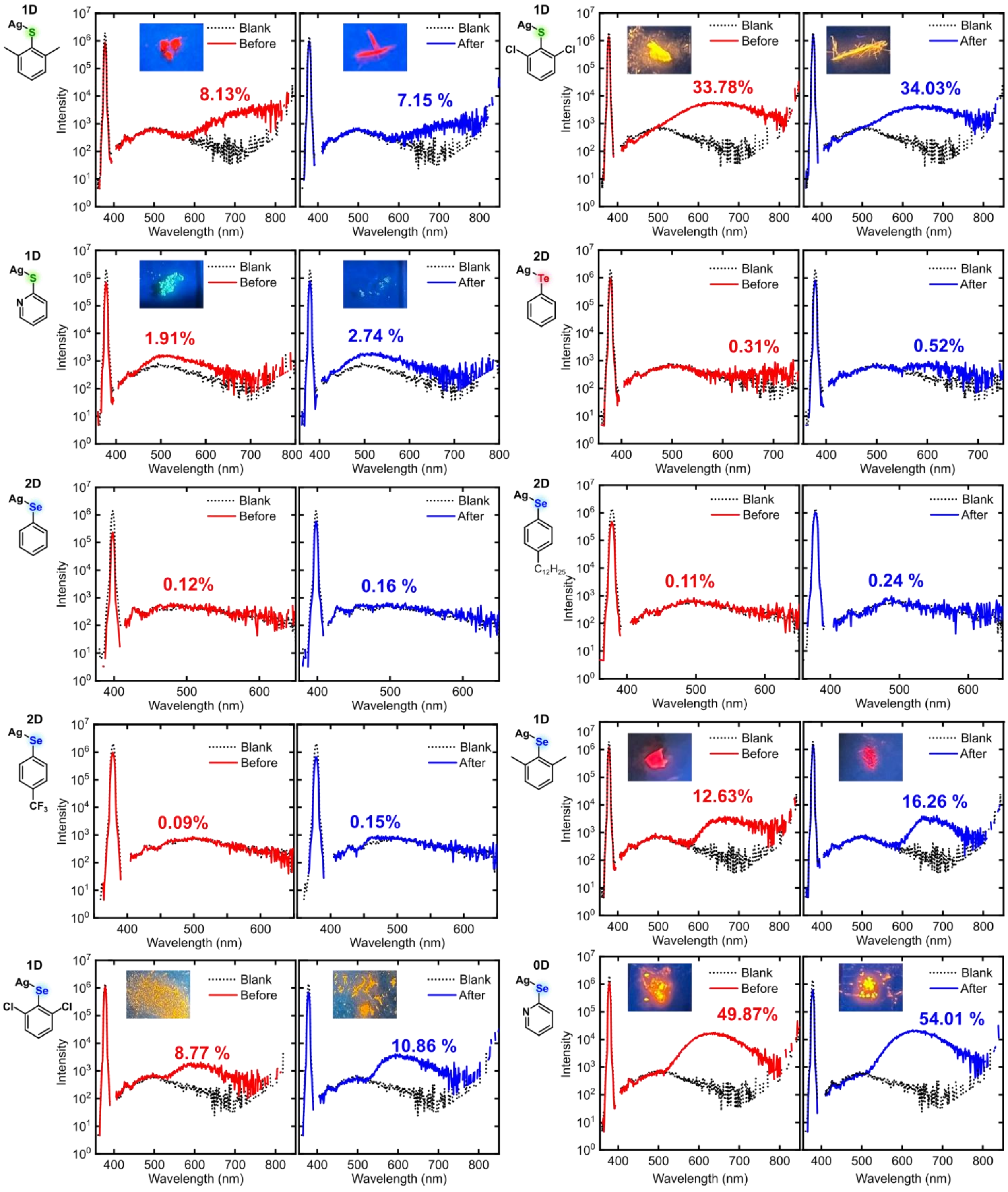

Figure S16. Photoluminescence Quantum Yields of Selected MOCs. Raw PL spectra of emissive MOCs and corresponding blanks used for photoluminescence quantum yield (PLQY) calculations, along with PLQY values of the MOCs before (red) and after (blue) recrystallization. Insets show optical images of the MOCs under UV light exposure.

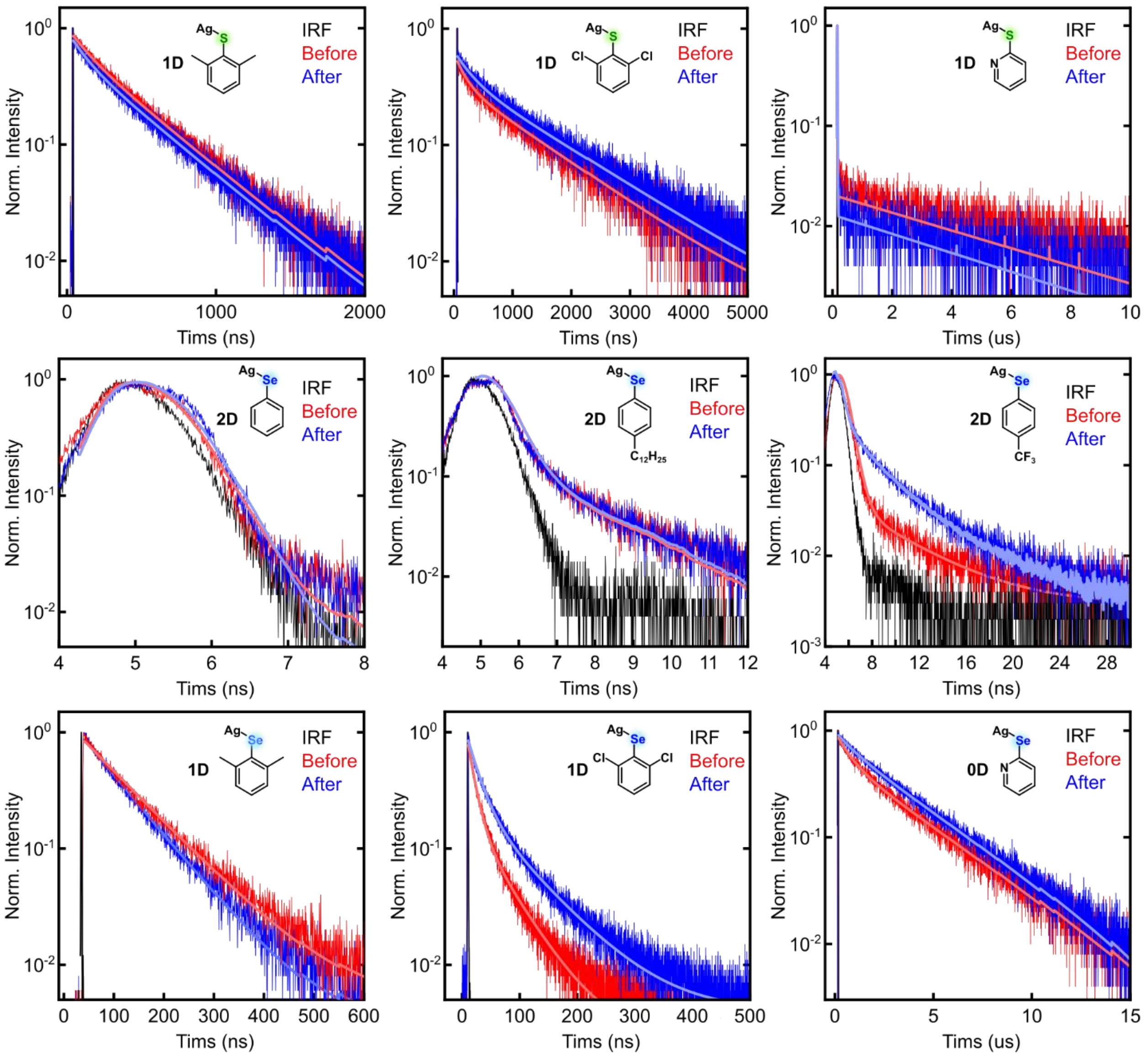

Figure S17. Time-Resolved Photoluminescence Decay of Selected MOCs. Decay profiles of emissive MOC before (red) and after (blue) recrystallization, together with instrument response function (IRF) and fitted curves.

12. Additional Data for Dissolution Mechanistic Study

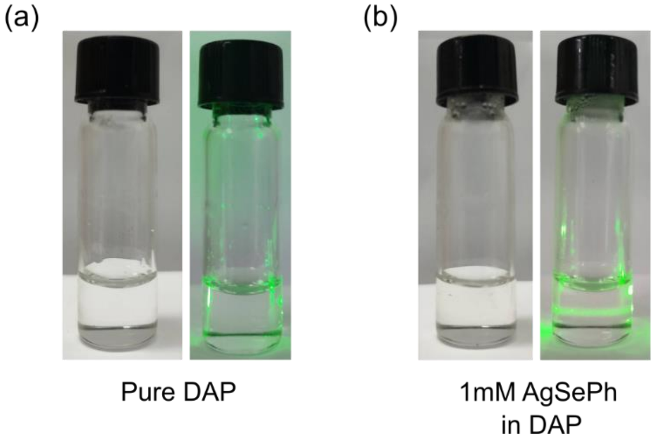

Figure S18. Tyndall Effect in AgSePh Solution. Optical images of (a) pure DAP and (b) 1 mM solution of AgSePh in DAP upon exposure to a green laser, indicating the presence of small suspended particles in solution.

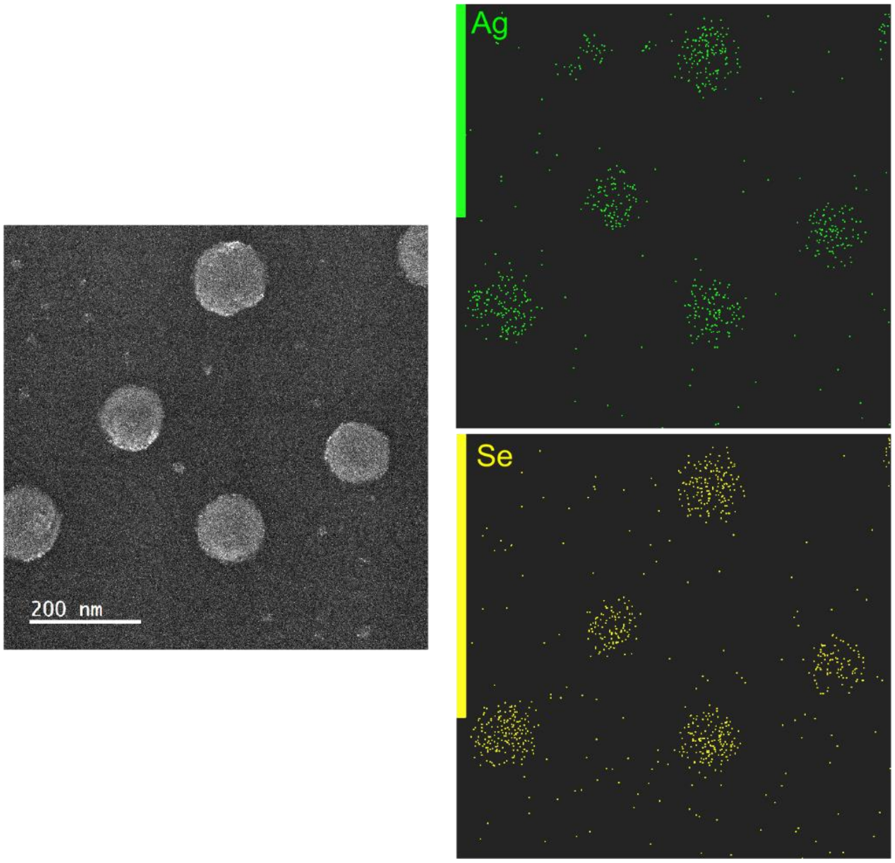

Figure S19. Transmission Electron Microscopy (TEM) Image of Quickly Dried AgSePh Solution Under Vacuum. Spherical particles observed in quickly-dried AgSePh solution along with energy dispersive X-ray (EDX) mappings.

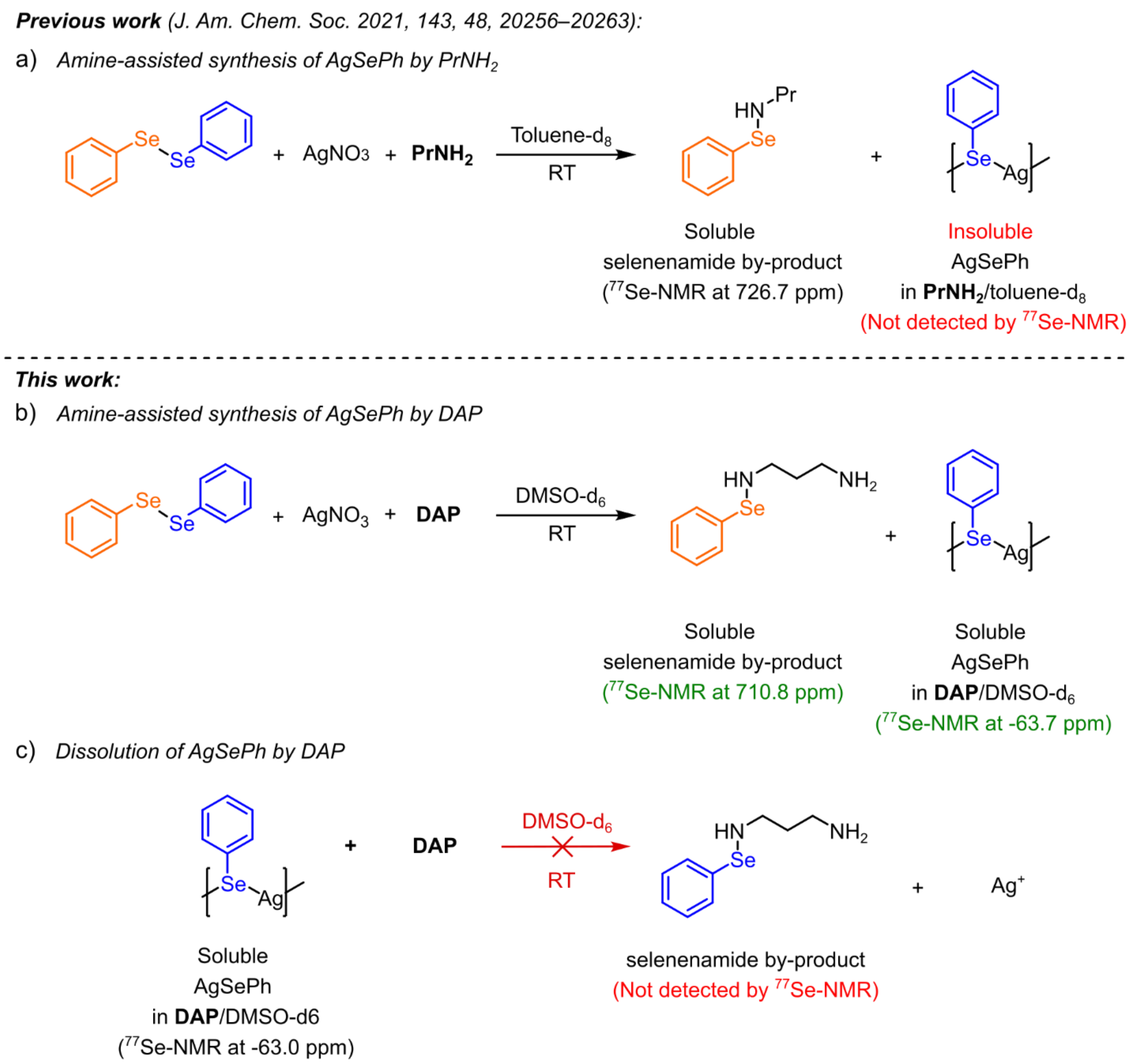

Figure S20. Difference in Reaction Process between Synthesis and Dissolution of AgSePh by Amine. (a) Reaction pathway of amine-assisted AgSePh synthesis producing a selenenamide by-product as reported in ref. [30]. (b) Reaction scheme of AgSePh's amine-assisted synthesis by DAP. (c) Reaction scheme of AgSePh's amine-assisted dissolution by DAP.

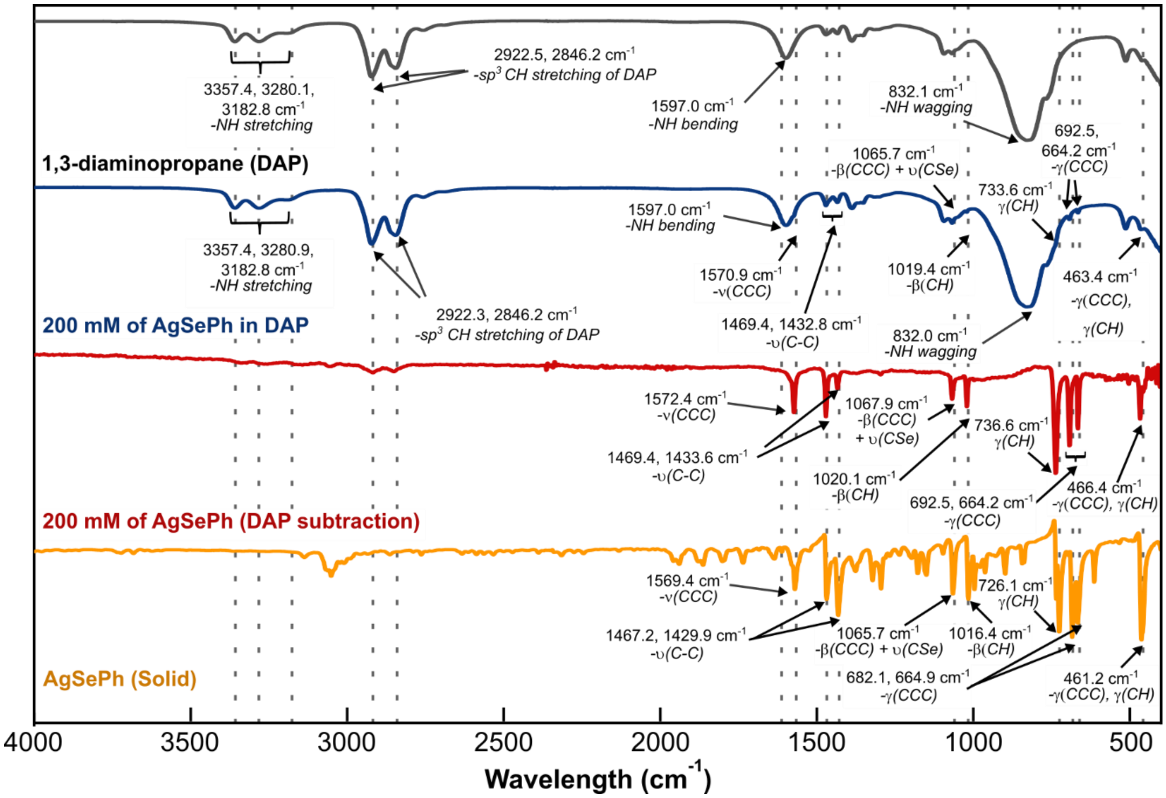

Figure S21. Chemical Nature Investigation by FTIR Spectroscopy of Dissolved MOCs. FTIR spectra of DAP, 200 mM AgSePh in DAP, 200 mM of AgSePh with background DAP subtraction, and AgSePh powder. The FTIR peaks of AgSePh powder, DAP, and 200 mM AgSePh in DAP were assigned based on the previous work by Maserati *et al.* [34] and Mendes *et al.* [35]

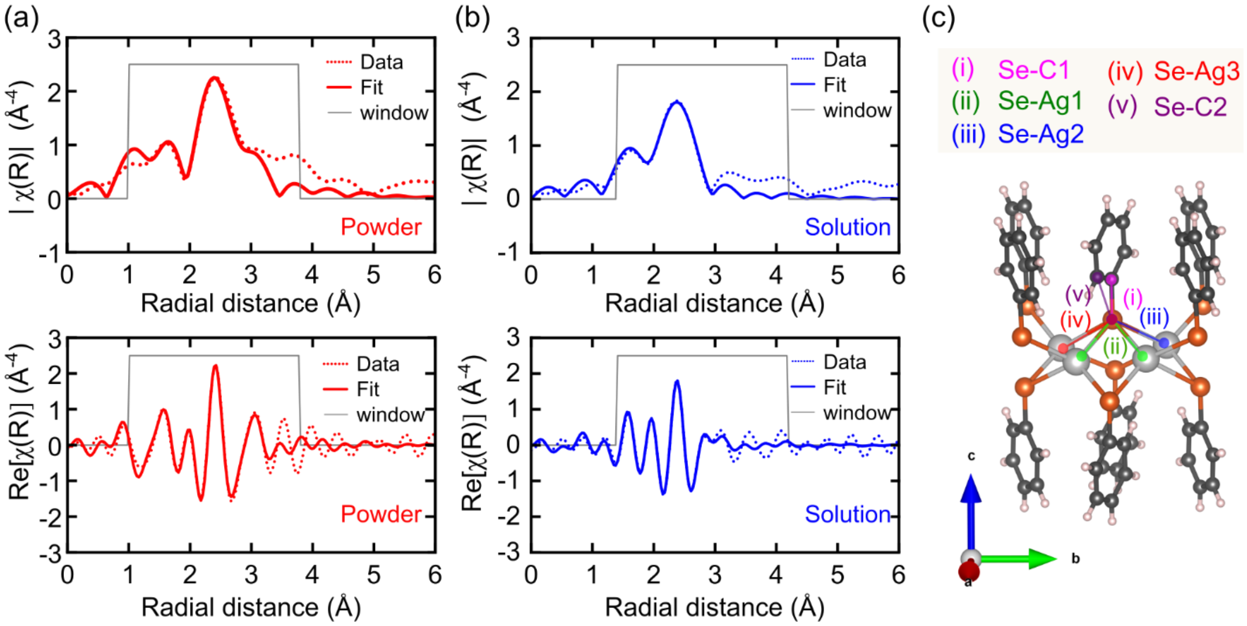

Figure S22. Se K-edge EXAFS Spectra of AgSePh and Corresponding Fits. (a) Fourier transform magnitude (top) and real component (bottom) of the Se K-edge EXAFS data and fit for AgSePh powder. (b) Fourier transform magnitude (top) and real component (bottom) of the Se K-edge EXAFS data and fit for AgSePh solution. (c) Model for EXAFS fitting derived from the crystal structure of AgSePh (CCDC2407479).

Table S5. Quantitative Se K-edge EXAFS data fitting results of AgSePh powder and solution. Fits were performed based on crystal structure of AgSePh (CCDC2407479) with S_0^2 = 0.80 applied to both datasets. The resulting R-factors are 0.0732 for AgSePh powder and 0.0613 for AgSePh solution.

Sample	Path	N	σ^2 (Å^2)	E_0 (eV)	R (Å)
AgSePh powder	Se–C1	1.000	0.00500	4.472	1.87662
	Se–Ag1	2.000	0.00142	4.472	2.59834
	Se–Ag2	1.000	0.00142	4.472	2.76404
	Se–Ag3	1.000	0.00100	4.472	2.82374
	Se–C2	2.000	0.00800	4.472	2.87247
AgSePh solution	Se–C1	1.000	0.02600	-4.772	1.90333
	Se–Ag1	2.000	0.006	-4.772	2.48661
	Se–Ag2	1.000	0.008	-4.772	2.63480
	Se–Ag3	1.000	0.070	-4.772	2.75670
	Se–C2	2.000	0.001	-4.772	2.84443

13. Confirmation of Space Group Assignment

Table S5 compares the crystal systems and space groups of our refined MOC structures obtained via SCXRD with previously reported structures determined by various techniques, including SCXRD, smSFX, and Le Bail refinement. While most of our refined structures are consistent with literature reports, some differences in space group assignments were observed when compared to data from different techniques. Notably, these discrepancies include:

1) AgEPh (E = S, Se, Te): primitive vs C-centered space groups.
2) AgSBu: $P2_1/c$ vs $P2_1/n$.
3) AgSPh-*p*F: *Pbca* vs $P2_12_12_1$.

These differences in crystallographic assignments may arise from variations in synthetic conditions, sample form, phase transformations at different measurement temperatures (100 K for SC-XRD vs. room temperature for smSFX and Le Bail refinement), and differences in diffraction data collection methods. However, previous studies indicate no evidence of phase transitions due to temperature changes or polymorph formation from different synthetic conditions in MOCs.[28] Instead, crystal quality and data collection methods have been proposed as the primary causes of space group discrepancies.

To investigate this hypothesis, we analyzed the systematic absence statistics for Bravais-lattice and space group selection in our SCXRD data to determine the most appropriate space group assignment. Additionally, we compared refinement indicators, including mean I/σ, final R index and peak residual, between our refinement and previously reported structures.

13.1 Confirmation of space group assignment for AgSPh, AgSePh, and AgTePh to $P2_1/c$

In this study, the crystal structures of AgSPh, AgSePh, and AgTePh obtained via recrystallization from DAP solutions were best assigned to the monoclinic $P2_1/c$ space group. This assignment aligns with previous reports for AgSePh and AgTePh based on SCXRD, suggesting that recrystallization does not alter the crystal structures of these MOCs. However, for AgSPh, our results differ from a prior refinement that assigned a $P2_1$ space group.[28] When comparing refinement indicators (mean intensity/σ, final R index, and peak/hole residuals), our refinement yielded superior results, supporting $P2_1/c$ as the most accurate space group assignment for AgSPh.

In contrast, previous reports assigned AgSPh, AgSePh, and AgTePh to C-centered Bravais lattices based on smSFX.[29] To verify whether a primitive Bravais lattice is a more appropriate assignment, we analyzed systematic absence statistics for Bravais-lattice selection (**Tables S6–S8**). The data showed that all three structures adhered strictly to primitive Bravais-lattice conditions, with no additional violating reflections. In contrast, nearly half of the observed reflections would need to be omitted if a C-centered Bravais lattice were enforced.

Furthermore, a direct comparison of our SC-XRD refinement with the smSFX-reported structures revealed that our data consistently provided better refinement indicators, particularly in mean I/σ, final R index, and peak residuals (**Figure S23)**. This discrepancy likely arises from the nature of the smSFX samples, which consisted of sub-micrometer colloidal particles with limited

lateral size and thickness. These microcrystals may not have been large or high-quality enough to generate all systematically weak X-ray reflections necessary for an accurate structural model. In contrast, SCXRD requires high-quality single crystals with a minimum size of ~20 μm, ensuring better diffraction data quality and enabling the most accurate crystal structure determination. This underscores the effectiveness of our recrystallization method in producing high-quality MOC single crystals suitable for precise SCXRD analysis.

Table S6. Systematic absence statistics for Bravais-lattice selection of AgSPh (CCDC-2413037).

Lattice exceptions	*P*	*A*	*B*	*C*	*I*	*F*	All
N(total)	0	15908	15898	15938	16001	23872	31824
N(Int > 3σ)	0	7546	8245	7175	9028	11483	16600
Mean intensity	0	13.3	22.6	21.0	23.9	19.0	28.4
Mean int/σ	0	5.2	6.8	6.1	7.4	6.1	7.1

N(total) indicates the number of violating diffraction peaks. *N (Int > 3σ)* indicates the number of intense violating diffraction peaks with intensities higher than three times the standard deviation (σ). Mean intensity represents the average intensity of violating reflections. *Mean int/σ* indicates the average of intensity/σ of violating reflections.

Table S7. Systematic absence statistics for Bravais-lattice selection of AgSePh (CCDC-2407479).

Lattice exceptions	*P*	*A*	*B*	*C*	*I*	*F*	All
N(total)	0	21088	21085	21751	21110	31962	42511
N(Int > 3σ)	0	13689	14385	12434	15319	20254	28661
Mean intensity	0	165.8	166.6	20.1	145.2	116.5	164.7
Mean int/σ	0	9.0	9.3	5.5	10.0	7.9	9.2

N(total) indicates the number of violating diffraction peaks. *N (Int > 3σ)* indicates the number of intense violating diffraction peaks with intensities higher than three times the standard deviation (σ). Mean intensity represents the average intensity of violating reflections. *Mean int/σ* indicates the average of intensity/σ of violating reflections.

Table S8. Systematic absence statistics for Bravais-lattice selection of AgTePh (CCDC-2412938).

Lattice exceptions	*P*	*A*	*B*	*C*	*I*	*F*	All
N(total)[a]	0	7226	7215	7179	7257	10810	14486
N (Int > 3σ)[b]	0	2490	2616	538	2601	2822	5216
Mean intensity[c]	0	20.6	20.7	0.8	18.7	14.1	21.3
Mean int/σ[d]	0	4.6	4.7	1.0	4.7	3.5	4.8

N(total) indicates the number of violating diffraction peaks. *N (Int > 3σ)* indicates the number of intense violating diffraction peaks with intensities higher than three times the standard deviation (σ). Mean intensity represents the average intensity of violating reflections. *Mean int/σ* indicates the average of intensity/σ of violating reflections.

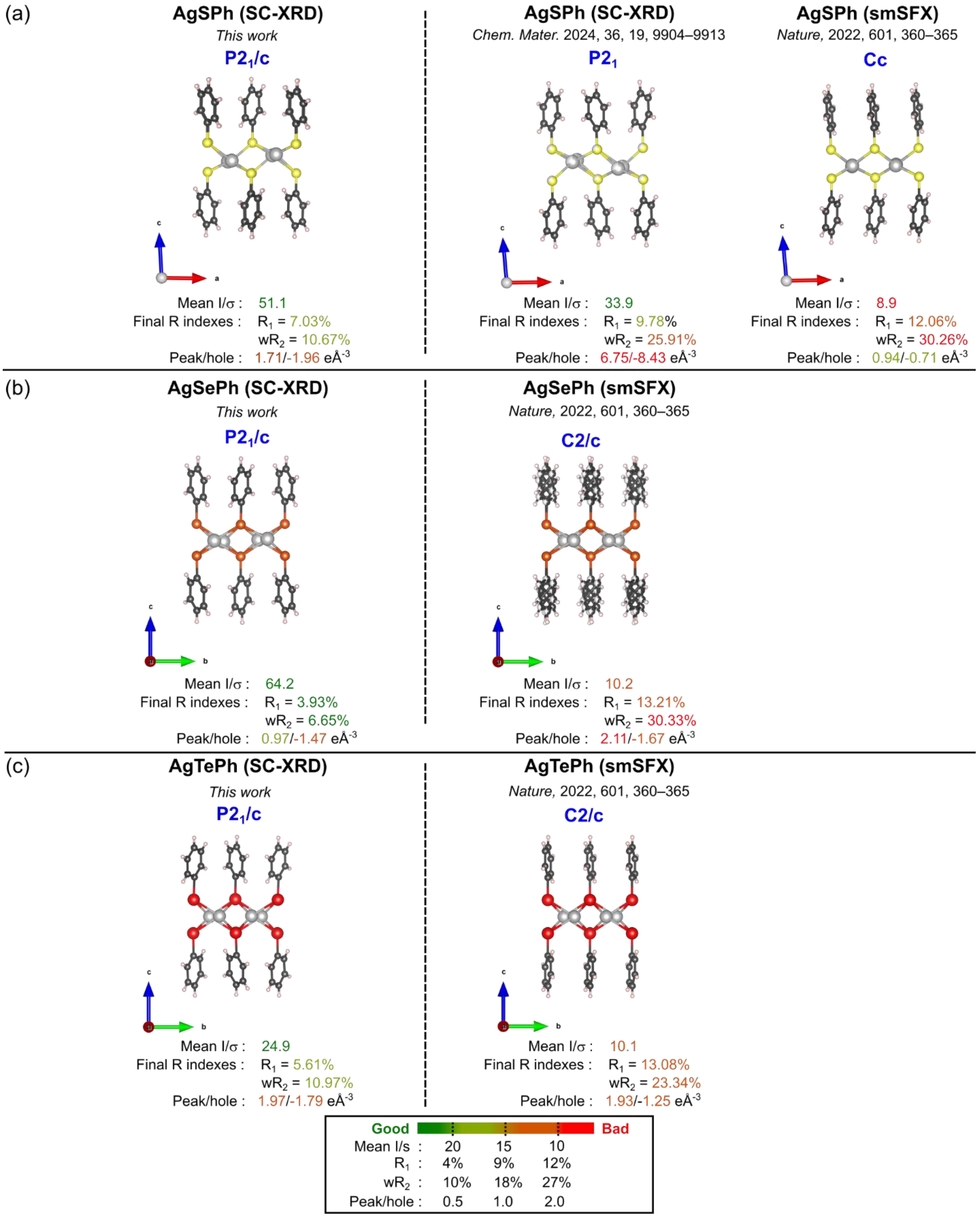

Figure S23. Comparison Between the Structural Refinement Results in This Work and Previous Reports of AgEPh (E = S, Se, Te).

13.2 Confirmation of space group assignment for AgSBu to *P*2_1*/c*

For AgSBu, our SCXRD analysis determined that the crystal structure is best assigned to the monoclinic *P*2_1*/c* space group. This contrasts with a previous report based on smSFX, which assigned AgSBu to *P*2_1*/n*.[23]

Table S9 presents the systematic absence statistics for Bravais-lattice selection, which strongly support a primitive lattice – consistent with both our refined structure and the literature. However, further analysis of systematic absence statistics for space group selection (**Table S10**) suggests that a C-centered space group is more appropriate than one incorporating n-glide planes. This conclusion is based on the significantly lower number of strong violating reflections (N $Int > 3\sigma$), which is nearly five times lower when assigning the structure to *P*2_1*/c* rather than *P*2_1*/n*.

Additionally, we attempted to refine the structure by forcing a *P*2_1*/n* assignment using our SC-XRD data. However, this approach was unsuccessful, as the structure could not be reliably determined under this constraint. A direct comparison of our refinement parameters with those reported by smSFX further supports our assignment, as our SC-XRD data yield superior refinement indicators, including higher Mean I/σ, a lower Final R-index, and better peak residuals (**Figure S24**).

Thus, we conclude that *P*2_1*/c* is the more accurate space group assignment for AgSBu.

Table S9. Systematic absence statistics for Bravais-lattice selection of AgSBu (CCDC-2413041).

Lattice exceptions	*P*	*A*	*B*	*C*	*I*	*F*	All
N(total)	0	13448	13411	13545	13365	20202	26961
N (Int > 3σ)	0	7389	7978	6823	8091	11095	15597
Mean intensity	0	254.5	258.5	169.3	257.8	227.3	252.3
Mean int/σ	0	7.3	7.7	6.8	7.8	7.3	7.7

N(total) indicates the number of violating diffraction peaks. *N (Int > 3σ)* indicates the number of intense violating diffraction peaks with intensities higher than three times the standard deviation (σ). Mean intensity represents the average intensity of violating reflections. *Mean int/σ* indicates the average of intensity/σ of violating reflections.

Table S10. Systematic absence statistics for space group selection of AgSBu (CCDC-2413041).

Space group exceptions	*-a-*	*-c-*	*-n-*
N(total)	1119	1028	1021
N (Int > 3σ)	474	99	573
Mean intensity	9.5	0.3	10.7
Mean int/σ	8.9	1.2	10.4

N(total) indicates the number of violating diffraction peaks. *N (Int > 3σ)* indicates the number of intense violating diffraction peaks with intensities higher than three times the standard deviation (σ). Mean intensity represents the average intensity of violating reflections. *Mean int/σ* indicates the average of intensity/σ of violating reflections.

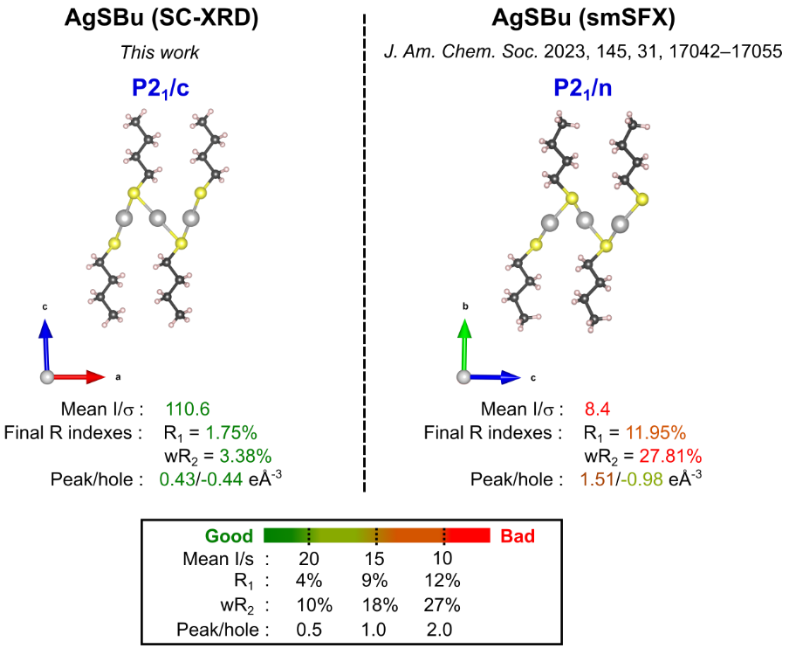

Figure S24. Comparison Between the Structural Refinement Results in This Work and Previous Reports of AgSBu.

13.3 Confirmation of space group assignment for AgSPh-*p*F to P_{bca}

Our SCXRD analysis determined that AgSPh-*p*F is best assigned to the orthorhombic *Pbca* space group. In contrast, the structure obtained from Le Bail refinement was reported as orthorhombic $P2_12_12_1$.[31]

Although the systematic absence statistics for Bravais-lattice selection and space group assignment (**Tables S11–S12**) initially suggested that $P2_12_12_1$ might be a reasonable choice, we found that modeling the structure in this space group was not feasible. The refinement process failed to yield a chemically valid structure, as the atomic positions became arbitrary and inconsistent with known bonding interactions.

It is important to highlight the fundamental difference between Le Bail refinement and SCXRD. Le Bail refinement, while useful for extracting unit cell parameters and assessing phase purity from powder diffraction data, does not determine atomic positions with high precision. In contrast, SCXRD provides detailed atomic coordinates, bond lengths, and angles, making it the more reliable method for precise structural determination. Given these considerations, we conclude that P_{bca} is the most appropriate space group assignment for AgSPh-pF, rather than $P2_12_12_1$.

Table S11. Systematic absence statistics for Bravais-lattice selection of AgSPh-*p*F (CCDC-2412916).

Lattice exceptions	*P*	*A*	*B*	*C*	*I*	*F*	All
N(total)	0	13448	13411	13545	13365	20202	26961
N (Int > 3σ)	0	7389	7978	6823	8091	11095	15597
Mean intensity	0	254.5	258.5	169.3	257.8	227.3	252.3
Mean int/σ	0	7.3	7.7	6.8	7.8	7.3	7.7

N(total) indicates the number of violating diffraction peaks. *N (Int > 3σ)* indicates the number of intense violating diffraction peaks with intensities higher than three times the standard deviation (σ). Mean intensity represents the average intensity of violating reflections. *Mean int/σ* indicates the average of intensity/σ of violating reflections.

Table S12. Systematic absence statistics for space group selection of AgSPh-*p*F (CCDC-2412916).

Space group exceptions	*b--*	*c--*	*n--*	*21--*	*-c-*	*-a-*	*-n-*	*-21-*	*--a*	*--b*	*--n*	*--21*
N (total)[a]	1561	1584	1575	13	103 1	1171	1060	23	235	240	231	123
N (Int > 3σ)	1	596	597	0	113	592	653	1	3	90	87	60
Mean intensity	1.5	466. 3	468. 9	1.2	5.9	426.6	472.3	2.8	2.1	60.5	62.3	9.2
Mean int/σ	0.4	6.1	6.2	0.4	1.4	10.2	11.3	0.8	0.5	4.3	4.4	2.9

N(total) indicates the number of violating diffraction peaks. *N (Int > 3σ)* indicates the number of intense violating diffraction peaks with intensities higher than three times the standard deviation (σ). Mean intensity represents the average intensity of violating reflections. *Mean int/σ* indicates the average of intensity/σ of violating reflections.

14. Additional Crystallographic Information

14.1 AgSPh (CCDC-2413037)

Table S13. Fractional Atomic Coordinates (×10^4) and Equivalent Isotropic Displacement Parameters (Å^2×10^3) for AgSPh (2413037). U_{eq} is defined as 1/3 of the trace of the orthogonalised U_{IJ} tensor.

**Atom**	***x***	***y***	***z***	**U(eq)**
Ag1	2231.1(8)	7403.6(11)	7378.9(2)	21.98(17)
Ag2	7286.8(8)	7407.6(11)	7583.9(2)	22.89(17)
S1	4849(3)	5432(3)	7049.7(7)	16.5(4)
S2	10093(3)	5444(3)	7932.7(7)	17.3(4)
C7	10236(10)	6401(13)	8533(3)	14.8(15)
C1	4693(10)	6347(13)	6445(3)	12.7(14)
C2	5458(11)	8434(14)	6312(3)	18.9(16)
C12	9540(11)	5013(15)	8889(3)	19.2(16)
C8	11080(11)	8470(14)	8665(3)	19.7(17)
C9	11228(12)	9167(16)	9138(3)	23.5(18)
C6	3872(10)	4900(15)	6090(3)	18.3(16)
C3	5388(11)	9087(16)	5837(3)	23.9(18)
C5	3822(11)	5605(14)	5617(3)	20.2(17)
C4	4576(10)	7658(15)	5487(3)	19.1(16)
C10	10542(11)	7791(15)	9486(3)	21.2(17)
C11	9694(11)	5739(15)	9362(3)	21.5(17)

Table S14. Anisotropic Displacement Parameters ($Å^2 \times 10^3$) for AgSPh (2413037). The Anisotropic displacement factor exponent takes the form: $-2\pi^2[h^2a^{*2}U_{11}+2hka^*b^*U_{12}+\ldots]$.

Atom	U_{11}	U_{22}	U_{33}	U_{23}	U_{13}	U_{12}
Ag1	21.9(3)	14.0(3)	31.2(4)	0.5(3)	10.3(2)	1.3(3)
Ag2	21.2(3)	13.2(3)	32.8(4)	-0.8(3)	-8.4(2)	1.2(3)
S1	13.9(9)	15.6(9)	20.1(9)	1.2(8)	0.8(7)	0.1(7)
S2	14.2(9)	15.1(9)	22.7(10)	0.8(8)	3.0(7)	0.5(7)
C7	13(4)	14(4)	17(4)	0(3)	0(3)	3(3)
C1	11(3)	13(4)	14(3)	-1(3)	2(3)	1(3)
C2	20(4)	12(4)	24(4)	-5(3)	2(3)	-2(3)
C12	16(4)	20(4)	21(4)	3(3)	-2(3)	-3(3)
C8	22(4)	13(4)	25(4)	5(3)	0(3)	-2(3)
C9	23(4)	23(4)	24(4)	-1(4)	-5(3)	-1(4)
C6	15(4)	21(4)	18(4)	0(3)	-1(3)	-1(3)
C3	21(4)	24(4)	27(4)	5(4)	5(3)	-1(4)
C5	17(4)	19(4)	25(4)	-4(3)	-1(3)	1(3)
C4	19(4)	19(4)	19(4)	2(3)	2(3)	2(3)
C10	21(4)	27(5)	15(4)	1(3)	-3(3)	2(4)
C11	23(4)	19(4)	22(4)	3(3)	-1(3)	-4(3)

Table S15. Bond Lengths for AgSPh (2413037).

Atom	Atom	Length/Å	Atom	Atom	Length/Å
Ag1	Ag2[1]	2.9247(9)	C7	C8	1.390(11)
Ag1	Ag2[2]	2.9293(9)	C1	C2	1.398(11)
Ag1	S1	2.472(2)	C1	C6	1.408(11)
Ag1	S2[2]	2.566(2)	C2	C3	1.385(12)
Ag1	S2[3]	2.554(2)	C12	C11	1.391(12)
Ag2	S1[2]	2.614(2)	C8	C9	1.385(12)
Ag2	S1	2.527(2)	C9	C10	1.386(12)
Ag2	S2	2.493(2)	C6	C5	1.389(11)
S1	C1	1.774(8)	C3	C4	1.389(12)
S2	C7	1.772(8)	C5	C4	1.374(12)
C7	C12	1.408(11)	C10	C11	1.378(12)

[1]1-X,-1/2+Y,3/2-Z; [2]1-X,1/2+Y,3/2-Z; [3]-1+X,+Y,+Z

Table S16. Bond Angles for AgSPh (2413037).

Atom	Atom	Atom	Angle/°
Ag2[1]	Ag1	Ag2[2]	165.80(3)
S1	Ag1	Ag2[1]	57.21(5)
S1	Ag1	Ag2[2]	112.21(5)
S1	Ag1	S2[3]	123.29(7)
S1	Ag1	S2[2]	134.99(7)
S2[3]	Ag1	Ag2[2]	119.89(5)
S2[3]	Ag1	Ag2[1]	67.30(5)
S2[2]	Ag1	Ag2[1]	140.46(5)
S2[2]	Ag1	Ag2[2]	53.47(5)
S2[3]	Ag1	S2[2]	95.28(4)
Ag1[2]	Ag2	Ag1[1]	165.80(3)
S1	Ag2	Ag1[2]	123.40(5)
S1[2]	Ag2	Ag1[2]	52.65(5)
S1[2]	Ag2	Ag1[1]	136.66(5)
S1	Ag2	Ag1[1]	69.47(5)
S1	Ag2	S1[2]	96.88(4)
S2	Ag2	Ag1[1]	55.79(5)
S2	Ag2	Ag1[2]	110.21(5)
S2	Ag2	S1[2]	130.89(7)
S2	Ag2	S1	123.77(7)
Ag1	S1	Ag2[1]	70.14(5)
Ag1	S1	Ag2	95.97(7)
Ag2	S1	Ag2[1]	119.38(8)
C1	S1	Ag1	102.7(2)

Atom	Atom	Atom	Angle/°
C1	S1	Ag2[1]	125.3(3)
C1	S1	Ag2	115.3(3)
Ag1[4]	S2	Ag1[1]	121.43(8)
Ag2	S2	Ag1[4]	94.77(7)
Ag2	S2	Ag1[1]	70.75(6)
C7	S2	Ag1[4]	116.2(3)
C7	S2	Ag1[1]	122.3(3)
C7	S2	Ag2	102.8(3)
C12	C7	S2	119.7(6)
C8	C7	S2	121.3(6)
C8	C7	C12	118.9(7)
C2	C1	S1	121.0(6)
C2	C1	C6	119.2(7)
C6	C1	S1	119.7(6)
C3	C2	C1	120.4(8)
C11	C12	C7	119.7(8)
C9	C8	C7	120.7(8)
C8	C9	C10	120.1(8)
C5	C6	C1	118.9(8)
C2	C3	C4	120.2(8)
C4	C5	C6	121.7(8)
C5	C4	C3	119.4(8)
C11	C10	C9	120.0(8)
C10	C11	C12	120.6(8)

[1]1-X,-1/2+Y,3/2-Z; [2]1-X,1/2+Y,3/2-Z; [3]-1+X,+Y,+Z; [4]1+X,+Y,+Z

Table S17. Torsion Angles for AgSPh (2413037).

A	B	C	D	Angle/°	A	B	C	D	Angle/°
Ag1	S1	C1	C2	84.7(6)	S2	C7	C12	C11	178.4(6)
Ag1	S1	C1	C6	-98.7(6)	S2	C7	C8	C9	-178.2(6)
Ag1[1]	S2	C7	C12	-161.3(5)	C7	C12	C11	C10	-0.8(12)
Ag1[2]	S2	C7	C12	21.6(7)	C7	C8	C9	C10	0.5(12)
Ag1[1]	S2	C7	C8	16.6(7)	C1	C2	C3	C4	-0.8(12)
Ag1[2]	S2	C7	C8	-160.6(5)	C1	C6	C5	C4	1.0(12)
Ag2	S1	C1	C2	-18.3(7)	C2	C1	C6	C5	-1.0(11)
Ag2[2]	S1	C1	C2	159.2(5)	C2	C3	C4	C5	0.8(12)
Ag2[2]	S1	C1	C6	-24.2(7)	C12	C7	C8	C9	-0.3(12)
Ag2	S1	C1	C6	158.3(5)	C8	C7	C12	C11	0.5(12)
Ag2	S2	C7	C12	96.7(6)	C8	C9	C10	C11	-0.8(13)
Ag2	S2	C7	C8	-85.4(6)	C9	C10	C11	C12	0.9(13)
S1	C1	C2	C3	177.5(6)	C6	C1	C2	C3	0.9(11)
S1	C1	C6	C5	-177.6(6)	C6	C5	C4	C3	-0.9(12)

[1]1+X,+Y,+Z; [2]1-X,-1/2+Y,3/2-Z

Table S18. Hydrogen Atom Coordinates (Å×10^4) and Isotropic Displacement Parameters (Å^2×10^3) for AgSPh (2413037).

Atom	*x*	*y*	*z*	U(eq)
H2	6017.02	9390.98	6544.79	23
H12	8979.54	3617.21	8808.03	23
H8	11550.77	9395.98	8432.93	24
H9	11786.81	10560.34	9221.44	28
H6	3372.4	3493.93	6170.51	22
H3	5887.79	10486.94	5752.38	29
H5	3264.17	4664.43	5381.27	24
H4	4542.08	8086.7	5167.37	23
H10	10655.11	8252.08	9804.32	25
H11	9220.36	4831.67	9596.73	26

14.2 AgSePh (CCDC-2407479)

Table S19. Fractional Atomic Coordinates (×10[4]) and Equivalent Isotropic Displacement Parameters (Å[2]×10[3]) for AgSePh (2407479). U_{eq} is defined as 1/3 of the trace of the orthogonalised U_{IJ} tensor.

Atom	*x*	*y*	*z*	U(eq)
Ag1	7376.4(6)	3703.9(5)	2487.2(2)	22.53(9)
Ag2	7593.3(6)	7681.2(5)	2538.9(2)	22.96(9)
Se1	4547.2(7)	5698.0(6)	2962.9(2)	13.98(10)
Se2	10415.8(7)	5688.3(6)	2054.5(2)	14.12(10)
C1	5810(7)	5698(6)	3601.2(14)	14.6(8)
C7	9134(7)	5640(6)	1418.2(14)	14.8(8)
C12	6979(8)	6385(7)	1287.3(16)	19.9(9)
C6	7963(7)	4943(6)	3730.8(16)	19.2(9)
C8	10419(8)	4867(6)	1089.6(16)	19.2(9)
C2	4491(8)	6402(6)	3933.1(16)	19.1(9)
C11	6142(8)	6418(7)	822.4(16)	22.6(9)
C5	8784(8)	4866(7)	4198.2(16)	23.2(10)
C9	9553(8)	4892(7)	626.7(16)	24.1(10)
C3	5332(8)	6334(7)	4395.9(17)	24.2(10)
C4	7469(9)	5568(7)	4528.8(16)	24.2(10)
C10	7430(8)	5664(7)	494.6(16)	24.0(10)

Table S20. Anisotropic Displacement Parameters (Å[2]×10[3]) for AgSePh (2407479). The Anisotropic displacement factor exponent takes the form: $-2\pi^2[h^2a^{*2}U_{11}+2hka^*b^*U_{12}+\ldots]$.

Atom	U_{11}	U_{22}	U_{33}	U_{23}	U_{13}	U_{12}
Ag1	20.60(17)	13.64(17)	33.7(2)	-1.74(13)	4.42(14)	-1.19(12)
Ag2	20.98(17)	13.38(17)	34.8(2)	-1.48(14)	4.11(14)	-1.43(13)
Se1	14.44(19)	10.8(2)	16.04(19)	0.16(15)	-1.57(14)	-0.07(15)
Se2	14.05(19)	11.4(2)	16.2(2)	0.06(15)	-1.67(14)	0.06(15)
C1	16.4(19)	13(2)	14.0(19)	-0.9(15)	-0.9(15)	0.0(15)
C7	15.1(19)	14(2)	15.1(19)	2.2(15)	-1.6(15)	-2.7(15)
C12	17(2)	21(2)	20(2)	-1.5(18)	0.3(16)	2.1(17)
C6	17(2)	20(2)	21(2)	-0.5(17)	-0.5(16)	2.5(17)
C8	16.0(19)	18(2)	23(2)	0.4(17)	0.8(16)	4.3(17)
C2	16.7(19)	18(2)	22(2)	0.3(17)	0.7(16)	0.0(17)
C11	18(2)	25(2)	24(2)	1.8(19)	-3.7(17)	2.6(18)
C5	20(2)	24(2)	23(2)	-0.2(19)	-7.0(17)	1.0(18)
C9	28(2)	24(2)	21(2)	-1.8(19)	4.9(18)	4(2)
C3	26(2)	23(2)	24(2)	-3.3(19)	5.4(18)	-0.2(19)
C4	30(2)	25(3)	16(2)	-0.5(18)	-2.9(18)	-3(2)
C10	26(2)	27(3)	18(2)	1.7(18)	-3.5(18)	-3(2)

Table S21. Bond Lengths for AgSePh (2407479).

Atom	Atom	Length/Å	Atom	Atom	Length/Å
Ag1	Ag2[1]	3.0461(5)	C1	C6	1.392(6)
Ag1	Ag2[2]	2.9967(5)	C1	C2	1.394(6)
Ag1	Se1[2]	2.7347(6)	C7	C12	1.391(6)
Ag1	Se1	2.6900(6)	C7	C8	1.394(6)
Ag1	Se2[1]	2.8152(6)	C12	C11	1.391(6)
Ag1	Se2	2.7013(6)	C6	C5	1.397(6)
Ag2	Se1[3]	2.8567(6)	C8	C9	1.390(6)
Ag2	Se1	2.6901(6)	C2	C3	1.387(6)
Ag2	Se2[4]	2.6976(6)	C11	C10	1.388(7)
Ag2	Se2	2.7017(6)	C5	C4	1.389(7)
Se1	C1	1.928(4)	C9	C10	1.383(7)
Se2	C7	1.926(4)	C3	C4	1.389(7)

[1]2-X,-1/2+Y,1/2-Z; [2]1-X,-1/2+Y,1/2-Z; [3]1-X,1/2+Y,1/2-Z; [4]2-X,1/2+Y,1/2-Z

Table S22. Bond Angles for AgSePh (2407479).

Atom	Atom	Atom	Angle/°	Atom	Atom	Atom	Angle/°
Ag2^{1}	Ag1	Ag2^{2}	151.299(18)	Ag1	Se1	Ag2	65.322(15)
Se1^{1}	Ag1	Ag2^{2}	98.298(15)	Ag2	Se1	Ag1^{3}	67.059(14)
Se1	Ag1	Ag2^{2}	142.400(18)	Ag2	Se1	Ag2^{1}	117.096(16)
Se1	Ag1	Ag2^{1}	60.023(13)	C1	Se1	Ag1^{3}	124.08(13)
Se1^{1}	Ag1	Ag2^{1}	55.759(13)	C1	Se1	Ag1	107.45(13)
Se1	Ag1	Se1^{1}	115.708(14)	C1	Se1	Ag2^{1}	126.72(13)
Se1	Ag1	Se2^{2}	117.042(18)	C1	Se1	Ag2	103.57(13)
Se1	Ag1	Se2	114.840(18)	Ag1	Se2	Ag1^{4}	119.288(16)
Se1^{1}	Ag1	Se2^{2}	75.461(15)	Ag1	Se2	Ag2	65.012(15)
Se2^{2}	Ag1	Ag2^{2}	54.723(13)	Ag2	Se2	Ag1^{4}	66.990(14)
Se2	Ag1	Ag2^{2}	55.595(13)	Ag2^{2}	Se2	Ag1^{4}	105.590(17)
Se2	Ag1	Ag2^{1}	143.613(18)	Ag2^{2}	Se2	Ag1	68.695(15)
Se2^{2}	Ag1	Ag2^{1}	102.465(15)	Ag2^{2}	Se2	Ag2	117.867(16)
Se2	Ag1	Se1^{1}	117.643(18)	C7	Se2	Ag1^{4}	125.80(13)
Se2	Ag1	Se2^{2}	110.242(14)	C7	Se2	Ag1	103.16(12)
Ag1^{3}	Ag2	Ag1^{4}	151.299(18)	C7	Se2	Ag2	108.20(13)
Se1	Ag2	Ag1^{4}	146.138(18)	C7	Se2	Ag2^{2}	121.04(13)
Se1^{3}	Ag2	Ag1^{4}	100.026(15)	C6	C1	Se1	120.6(3)
Se1^{3}	Ag2	Ag1^{3}	54.654(13)	C6	C1	C2	120.5(4)
Se1	Ag2	Ag1^{3}	57.181(13)	C2	C1	Se1	118.8(3)
Se1	Ag2	Se1^{3}	111.766(14)	C12	C7	Se2	120.7(3)
Se1	Ag2	Se2	114.822(18)	C12	C7	C8	120.5(4)
Se1	Ag2	Se2^{4}	120.795(19)	C8	C7	Se2	118.8(3)
Se2	Ag2	Ag1^{4}	58.287(13)	C7	C12	C11	119.6(4)
Se2	Ag2	Ag1^{3}	139.824(18)	C1	C6	C5	119.5(4)
Se2^{4}	Ag2	Ag1^{3}	100.622(16)	C9	C8	C7	119.4(4)
Se2^{4}	Ag2	Ag1^{4}	55.708(13)	C3	C2	C1	119.5(4)
Se2	Ag2	Se1^{3}	113.982(18)	C10	C11	C12	119.9(4)
Se2^{4}	Ag2	Se1^{3}	75.346(15)	C4	C5	C6	120.0(4)
Se2^{4}	Ag2	Se2	113.914(14)	C10	C9	C8	120.2(4)
Ag1	Se1	Ag1^{3}	115.534(16)	C2	C3	C4	120.4(4)
Ag1	Se1	Ag2^{1}	65.323(14)	C3	C4	C5	120.1(4)
Ag1^{3}	Se1	Ag2^{1}	103.490(17)	C9	C10	C11	120.4(4)

^{1}1-X,-1/2+Y,1/2-Z; ^{2}2-X,-1/2+Y,1/2-Z; ^{3}1-X,1/2+Y,1/2-Z; ^{4}2-X,1/2+Y,1/2-Z

Table S23. Torsion Angles for AgSePh (2407479).

A	B	C	D	Angle/°	A	B	C	D	Angle/°
Se1	C1	C6	C5	-176.4(4)	C12	C7	C8	C9	1.8(7)
Se1	C1	C2	C3	177.0(4)	C12	C11	C10	C9	-0.6(8)
Se2	C7	C12	C11	176.7(4)	C6	C1	C2	C3	-0.4(7)
Se2	C7	C8	C9	-177.3(4)	C6	C5	C4	C3	0.4(8)
C1	C6	C5	C4	-1.0(7)	C8	C7	C12	C11	-2.4(7)
C1	C2	C3	C4	-0.2(7)	C8	C9	C10	C11	-0.1(8)
C7	C12	C11	C10	1.8(7)	C2	C1	C6	C5	1.0(7)
C7	C8	C9	C10	-0.6(7)	C2	C3	C4	C5	0.2(8)

Table S24. Hydrogen Atom Coordinates (Å×10^4) and Isotropic Displacement Parameters (Å^2×10^3) for AgSePh (2407479).

Atom	*x*	*y*	*z*	U(eq)
H12	6085	6868.63	1513.69	24
H6	8867.48	4482.71	3503.19	23
H8	11871.64	4327.36	1181.09	23
H2	3026.71	6923.45	3842.89	23
H11	4689.72	6956.02	729.52	27
H5	10240.99	4334.98	4290.02	28
H9	10424.05	4377.96	400.34	29
H3	4440.46	6814.7	4623.17	29
H4	8032.75	5523.06	4846.47	29
H10	6849.53	5677.47	177.7	29

14.3 AgTePh (CCDC-2412938)

Table S25. Fractional Atomic Coordinates (×10^4^) and Equivalent Isotropic Displacement Parameters (Å^2×10^3) for AgTePh (2412938). U_{eq} is defined as 1/3 of the trace of the orthogonalised U_{IJ} tensor.

Atom	*x*	*y*	*z*	U(eq)
Te2	5680.7(16)	5763.7(11)	6998.2(3)	16.5(3)
Te1	-642.2(17)	5704.5(11)	7987.5(3)	16.8(3)
Ag1	2571(2)	3826.2(12)	7490.4(4)	21.6(3)
Ag2	2473(2)	7653.7(13)	7494.0(4)	22.2(3)
C4	2650(30)	5653(19)	9519(5)	21(3)
C1	870(20)	5622(19)	8651(4)	18(3)
C7	4170(30)	5730(18)	6330(5)	20(3)
C6	3000(20)	6440(20)	8744(5)	24(3)
C12	5450(20)	6469(19)	6002(5)	25(3)
C11	4600(30)	6380(20)	5557(5)	27(3)
C8	2050(30)	4980(20)	6242(5)	23(3)
C2	-390(30)	4840(20)	8976(5)	27(3)
C9	1240(30)	4890(20)	5795(5)	26(3)
C5	3840(30)	6460(20)	9182(5)	28(3)
C3	540(30)	4850(20)	9408(5)	26(3)
C10	2490(30)	5605(19)	5456(5)	22(3)

Table S26. Anisotropic Displacement Parameters (Å^2×10^3) for AgTePh (2412938). The Anisotropic displacement factor exponent takes the form: $-2\pi^2[h^2a^{*2}U_{11}+2hka^*b^*U_{12}+\ldots]$.

Atom	U_{11}	U_{22}	U_{33}	U_{23}	U_{13}	U_{12}
Te2	16.2(5)	12.5(5)	20.6(5)	-0.7(4)	-1.8(4)	0.2(4)
Te1	16.6(5)	13.0(5)	20.6(5)	0.0(4)	-1.8(4)	-0.1(4)
Ag1	22.9(5)	11.1(5)	31.0(5)	0.1(6)	3.7(4)	0.4(6)
Ag2	22.1(5)	14.1(5)	30.5(5)	0.3(5)	3.2(4)	1.4(6)
C4	26(8)	24(9)	13(7)	4(6)	-1(6)	7(7)
C1	22(8)	21(8)	10(6)	2(5)	-1(6)	0(6)
C7	30(9)	14(8)	17(7)	-1(6)	0(6)	5(6)
C6	28(8)	18(7)	28(8)	3(6)	15(6)	0(7)
C12	20(7)	15(7)	39(9)	2(6)	1(6)	3(6)
C11	28(8)	32(9)	23(8)	7(7)	10(6)	2(7)
C8	26(8)	21(8)	24(7)	1(6)	3(6)	4(7)
C2	24(8)	26(8)	32(8)	-10(7)	2(7)	-2(7)
C9	17(7)	28(9)	33(8)	4(7)	-7(6)	-3(7)
C5	23(8)	25(8)	36(9)	-7(7)	7(6)	0(7)
C3	29(8)	23(8)	24(8)	4(6)	0(6)	-1(7)
C10	19(8)	20(8)	25(8)	6(6)	-5(6)	1(6)

Table S27. Bond Lengths for AgTePh (2412938).

Atom	Atom	Length/Å	Atom	Atom	Length/Å
Te2	Ag1	2.8043(17)	C4	C5	1.39(2)
Te2	Ag1[1]	2.9104(14)	C4	C3	1.40(2)
Te2	Ag2[2]	2.9519(14)	C1	C6	1.40(2)
Te2	Ag2	2.8315(16)	C1	C2	1.38(2)
Te2	C7	2.155(14)	C7	C12	1.38(2)
Te1	Ag1[3]	2.9321(14)	C7	C8	1.37(2)
Te1	Ag1	2.8317(17)	C6	C5	1.38(2)
Te1	Ag2[4]	2.8749(14)	C12	C11	1.41(2)
Te1	Ag2	2.8133(16)	C11	C10	1.38(2)
Te1	C1	2.141(13)	C8	C9	1.40(2)
Ag1	Ag2[4]	3.0755(19)	C2	C3	1.38(2)
Ag1	Ag2	2.8568(13)	C9	C10	1.39(2)
Ag1	Ag2[2]	3.0215(19)			

[1]1-X,1/2+Y,3/2-Z; [2]1-X,-1/2+Y,3/2-Z; [3]-X,1/2+Y,3/2-Z; [4]-X,-1/2+Y,3/2-Z

Table S28. Bond Angles for AgTePh (2412938).

Atom	Atom	Atom	Angle/°	Atom	Atom	Atom	Angle/°
Ag1	Te2	$Ag1^{1}$	110.14(3)	Ag2	Ag1	$Ag2^{4}$	105.36(4)
Ag1	Te2	$Ag2^{2}$	63.26(4)	Ag2	Ag1	$Ag2^{2}$	107.99(4)
Ag1	Te2	Ag2	60.91(4)	Te2	Ag2	$Te2^{1}$	115.51(4)
$Ag1^{1}$	Te2	$Ag2^{2}$	103.58(4)	Te2	Ag2	$Te1^{3}$	111.56(5)
Ag2	Te2	$Ag1^{1}$	63.48(4)	Te2	Ag2	Ag1	59.07(4)
Ag2	Te2	$Ag2^{2}$	110.64(4)	$Te2^{1}$	Ag2	$Ag1^{1}$	55.98(4)
C7	Te2	Ag1	104.0(4)	Te2	Ag2	$Ag1^{1}$	59.53(4)
C7	Te2	$Ag1^{1}$	128.2(4)	$Te2^{1}$	Ag2	$Ag1^{3}$	95.32(4)
C7	Te2	$Ag2^{2}$	126.5(4)	Te2	Ag2	$Ag1^{3}$	144.12(6)
C7	Te2	Ag2	104.3(4)	Te1	Ag2	Te2	118.99(4)
Ag1	Te1	$Ag1^{3}$	112.47(4)	Te1	Ag2	$Te2^{1}$	111.21(5)
Ag1	Te1	$Ag2^{4}$	65.22(4)	$Te1^{3}$	Ag2	$Te2^{1}$	75.80(3)
$Ag2^{4}$	Te1	$Ag1^{3}$	104.98(4)	Te1	Ag2	$Te1^{3}$	116.23(5)
Ag2	Te1	Ag1	60.80(4)	Te1	Ag2	Ag1	59.92(4)
Ag2	Te1	$Ag1^{3}$	64.69(4)	$Te1^{3}$	Ag2	$Ag1^{1}$	96.27(4)
Ag2	Te1	$Ag2^{4}$	112.14(4)	Te1	Ag2	$Ag1^{1}$	141.88(6)
C1	Te1	$Ag1^{3}$	127.6(4)	$Te1^{3}$	Ag2	$Ag1^{3}$	56.71(4)
C1	Te1	Ag1	103.1(4)	Te1	Ag2	$Ag1^{3}$	59.52(4)
C1	Te1	Ag2	105.1(4)	Ag1	Ag2	$Te2^{1}$	141.36(6)
C1	Te1	$Ag2^{4}$	124.9(4)	Ag1	Ag2	$Te1^{3}$	142.83(6)
Te2	Ag1	$Te2^{2}$	117.73(4)	Ag1	Ag2	$Ag1^{1}$	105.67(4)
Te2	Ag1	$Te1^{4}$	113.15(5)	Ag1	Ag2	$Ag1^{3}$	107.70(4)
$Te2^{2}$	Ag1	$Te1^{4}$	75.58(3)	$Ag1^{1}$	Ag2	$Ag1^{3}$	146.59(5)
Te2	Ag1	Te1	119.29(4)	C5	C4	C3	118.5(14)
$Te2^{2}$	Ag1	$Ag2^{4}$	94.49(4)	C6	C1	Te1	119.5(10)
Te2	Ag1	$Ag2^{4}$	143.31(6)	C2	C1	Te1	117.6(11)
Te2	Ag1	Ag2	60.01(4)	C2	C1	C6	122.7(13)
Te2	Ag1	$Ag2^{2}$	60.75(4)	C12	C7	Te2	116.7(11)
$Te2^{2}$	Ag1	$Ag2^{2}$	56.98(3)	C8	C7	Te2	120.5(10)
Te1	Ag1	$Te2^{2}$	109.45(5)	C8	C7	C12	122.8(14)
Te1	Ag1	$Te1^{4}$	113.85(4)	C5	C6	C1	117.5(13)
Te1	Ag1	$Ag2^{2}$	142.72(6)	C7	C12	C11	118.9(14)
Te1	Ag1	Ag2	59.28(4)	C10	C11	C12	119.7(13)
$Te1^{4}$	Ag1	$Ag2^{4}$	55.79(3)	C7	C8	C9	117.4(14)
$Te1^{4}$	Ag1	$Ag2^{2}$	96.94(4)	C1	C2	C3	117.8(14)
Te1	Ag1	$Ag2^{4}$	58.07(4)	C10	C9	C8	121.5(14)
Ag2	Ag1	$Te2^{2}$	142.15(6)	C6	C5	C4	121.7(14)
Ag2	Ag1	$Te1^{4}$	142.08(6)	C2	C3	C4	121.8(14)
$Ag2^{2}$	Ag1	$Ag2^{4}$	146.59(5)	C11	C10	C9	119.7(14)

^{1}1-X,1/2+Y,3/2-Z; ^{2}1-X,-1/2+Y,3/2-Z; ^{3}-X,1/2+Y,3/2-Z; ^{4}-X,-1/2+Y,3/2-Z

Table S29. Torsion Angles for AgTePh (2412938).

A	B	C	D	Angle/°	A	B	C	D	Angle/°
Te2	C7	C12	C11	176.2(11)	C6	C1	C2	C3	-2(2)
Te2	C7	C8	C9	-175.6(11)	C12	C7	C8	C9	3(2)
Te1	C1	C6	C5	175.9(11)	C12	C11	C10	C9	-1(2)
Te1	C1	C2	C3	-177.5(11)	C8	C7	C12	C11	-3(2)
C1	C6	C5	C4	1(2)	C8	C9	C10	C11	2(2)
C1	C2	C3	C4	2(2)	C2	C1	C6	C5	0(2)
C7	C12	C11	C10	1(2)	C5	C4	C3	C2	0(2)
C7	C8	C9	C10	-3(2)	C3	C4	C5	C6	-1(2)

Table S30. Hydrogen Atom Coordinates (Å×10[4]) and Isotropic Displacement Parameters (Å[2]×10[3]) for AgTePh (2412938).

Atom	*x*	*y*	*z*	U(eq)
H4	3249.6	5652.93	9811.57	26
H6	3816.34	6960.87	8520.12	29
H12	6851.4	7012.85	6075.16	29
H11	5451.85	6847.94	5331.6	33
H8	1184.79	4545.34	6469.14	28
H2	-1817.05	4332.79	8906.17	33
H9	-161.18	4344	5723.98	32
H5	5232.7	7016.47	9254.07	33
H3	-259.9	4303.12	9629.62	31
H10	1905.94	5559.08	5162.92	26

14.4 AgSBu (CCDC-2413041)

Table S31. Fractional Atomic Coordinates (×10^4) and Equivalent Isotropic Displacement Parameters (Å^2×10^3) for AgSBu (2413041). U_{eq} is defined as 1/3 of the trace of the orthogonalised U_{IJ} tensor.

Atom	*x*	*y*	*z*	U(eq)
Ag1	5000	0	5000	11.45(7)
Ag2	0	5000	5000	11.63(7)
S1	1604.8(11)	1309.9(12)	5545.7(2)	10.92(10)
C2	2117(5)	4413(5)	6333.4(6)	13.8(4)
C3	4081(5)	5798(5)	6702.0(7)	16.7(4)
C1	3951(4)	3419(5)	5953.2(6)	13.4(4)
C4	2281(5)	6805(6)	7086.5(7)	20.8(5)

Table S32. Anisotropic Displacement Parameters (Å^2×10^3) for AgSBu (2413041). The Anisotropic displacement factor exponent takes the form: $-2\pi^2[h^2a^{*2}U_{11}+2hka^*b^*U_{12}+\ldots]$.

Atom	U_{11}	U_{22}	U_{33}	U_{23}	U_{13}	U_{12}
Ag1	11.43(11)	12.06(11)	10.96(11)	-1.35(8)	1.71(8)	0.39(8)
Ag2	11.35(11)	11.54(11)	11.88(11)	0.42(8)	-1.11(8)	0.27(8)
S1	11.2(2)	11.7(2)	9.9(2)	-0.63(19)	0.40(16)	-0.1(2)
C2	13.9(10)	16.2(10)	11.4(9)	-1.4(8)	0.7(8)	0.3(8)
C3	16.9(10)	20.5(11)	12.6(9)	-3.0(9)	0.3(8)	-1.2(9)
C1	13.5(10)	16.3(10)	10.3(9)	-1.7(8)	0.3(7)	-0.3(8)
C4	23.1(11)	26.1(13)	13.1(10)	-4.1(9)	0.1(8)	0.1(10)

Table S33. Bond Lengths for AgSBu (2413041).

Atom	Atom	Length/Å	Atom	Atom	Length/Å
Ag1	Ag2^1	3.08811(5)	Ag2	S1	2.3951(5)
Ag1	Ag2^2	3.08811(5)	Ag2	S1^5	2.3952(5)
Ag1	Ag2^3	3.08811(5)	S1	C1	1.841(2)
Ag1	Ag2	3.08811(5)	C2	C3	1.529(3)
Ag1	S1	2.3852(5)	C2	C1	1.523(3)
Ag1	S1^4	2.3853(5)	C3	C4	1.527(3)

^{1}1+X,+Y,+Z; ^{2}+X,-1+Y,+Z; ^{3}1+X,-1+Y,+Z; ^{4}1-X,-Y,1-Z; ^{5}-X,1-Y,1-Z

Table S34. Bond Angles for AgSBu (2413041).

Atom	Atom	Atom	Angle/°	Atom	Atom	Atom	Angle/°
$Ag2^1$	Ag1	$Ag2^2$	180.0	$Ag1^7$	Ag2	Ag1	92.847(1)
$Ag2^2$	Ag1	$Ag2^3$	92.847(1)	$Ag1^5$	Ag2	$Ag1^7$	180.0
Ag2	Ag1	$Ag2^3$	180.0	$Ag1^7$	Ag2	$Ag1^6$	87.153(2)
$Ag2^1$	Ag1	$Ag2^3$	87.153(2)	S1	Ag2	Ag1	49.620(12)
$Ag2^1$	Ag1	Ag2	92.847(1)	S1	Ag2	$Ag1^5$	104.860(12)
$Ag2^2$	Ag1	Ag2	87.153(2)	$S1^8$	Ag2	Ag1	130.379(12)
S1	Ag1	Ag2	49.899(12)	S1	Ag2	$Ag1^7$	75.140(12)
S1	Ag1	$Ag2^1$	108.773(13)	$S1^8$	Ag2	$Ag1^7$	104.860(12)
$S1^4$	Ag1	$Ag2^3$	49.900(12)	$S1^8$	Ag2	$Ag1^5$	75.140(12)
S1	Ag1	$Ag2^2$	71.227(13)	S1	Ag2	$Ag1^6$	130.380(12)
$S1^4$	Ag1	$Ag2^2$	108.773(13)	$S1^8$	Ag2	$Ag1^6$	49.621(12)
$S1^4$	Ag1	Ag2	130.100(12)	S1	Ag2	$S1^8$	180.0
S1	Ag1	$Ag2^3$	130.101(12)	Ag1	S1	Ag2	80.482(15)
$S1^4$	Ag1	$Ag2^1$	71.227(13)	C1	S1	Ag1	103.76(7)
S1	Ag1	$S1^4$	180.0	C1	S1	Ag2	108.00(7)
$Ag1^5$	Ag2	Ag1	87.153(2)	C1	C2	C3	111.88(17)
$Ag1^5$	Ag2	$Ag1^6$	92.847(2)	C4	C3	C2	112.63(18)
Ag1	Ag2	$Ag1^6$	180.0	C2	C1	S1	110.80(14)

^{1}1+X,+Y,+Z; ^{2}+X,-1+Y,+Z; ^{3}1+X,-1+Y,+Z; ^{4}1-X,-Y,1-Z; ^{5}+X,1+Y,+Z; ^{6}-1+X,1+Y,+Z; ^{7}-1+X,+Y,+Z; ^{8}-X,1-Y,1-Z

Table S35. Torsion Angles for AgSBu (2413041).

A	B	C	D	Angle/°	A	B	C	D	Angle/°
Ag1	S1	C1	C2	-179.39(13)	C3	C2	C1	S1	172.78(15)
Ag2	S1	C1	C2	96.32(14)	C1	C2	C3	C4	-179.92(19)

Table S36. Hydrogen Atom Coordinates (Å×10^4) and Isotropic Displacement Parameters (Å^2×10^3) for AgSBu (2413041).

**Atom**	***x***	***y***	***z***	**U(eq)**
H2A	1027.23	2568.07	6441.37	17
H2B	618.13	5994.92	6234.27	17
H3A	5582.96	4214.96	6799.64	20
H3B	5169.89	7640.48	6593.08	20
H1A	5611.1	2041.27	6057.66	16
H1B	4828.59	5300.12	5819.88	16
H4A	813.43	8400.35	6993.1	31
H4B	3635	7681.44	7312.57	31
H4C	1239.52	4979.14	7200.77	31

14.5 AgSPh-*p*CH_3 (CCDC-2412937)

Table S37. Fractional Atomic Coordinates (×10[4]) and Equivalent Isotropic Displacement Parameters ($Å^2$×10[3]) for AgSPh-*p*CH_3 (2412937). U_{eq} is defined as 1/3 of the trace of the orthogonalised U_{IJ} tensor.

Atom	*x*	*y*	*z*	U(eq)
Ag1	2117.8(10)	4971.7(14)	7404.1(2)	20.8(2)
S1	4880(3)	3358(4)	7100.9(7)	19.3(5)
C1	4937(12)	4696(16)	6607(3)	15.1(19)
C2	4185(13)	3525(18)	6271(3)	20(2)
C6	5750(13)	6846(17)	6548(3)	21(2)
C3	4306(14)	4557(17)	5877(3)	23(2)
C5	5870(14)	7820(18)	6159(3)	24(2)
C4	5155(14)	6668(18)	5815(3)	22(2)
C7	5304(16)	7730(20)	5390(3)	31(3)

Table S38. Anisotropic Displacement Parameters ($Å^2$×10[3]) for AgSPh-*p*CH_3 (2412937). The Anisotropic displacement factor exponent takes the form: $-2\pi^2[h^2a^{*2}U_{11}+2hka^*b^*U_{12}+\ldots]$.

Atom	U_{11}	U_{22}	U_{33}	U_{23}	U_{13}	U_{12}
Ag1	22.2(4)	13.9(3)	26.4(4)	-0.1(3)	6.0(3)	0.8(3)
S1	19.7(12)	15.6(11)	22.7(11)	2.2(10)	-1.2(10)	-0.5(11)
C1	12(4)	14(5)	19(4)	-4(4)	13(4)	-4(4)
C2	20(5)	20(5)	19(5)	0(4)	-4(4)	-2(5)
C6	19(5)	13(5)	32(5)	-4(4)	1(4)	-1(4)
C3	25(5)	23(6)	22(5)	-4(4)	-8(4)	3(5)
C5	23(5)	17(5)	31(5)	1(4)	0(4)	-4(4)
C4	24(5)	21(5)	22(5)	0(4)	2(4)	4(5)
C7	32(6)	33(6)	29(6)	2(5)	1(5)	2(5)

Table S39. Bond Lengths for AgSPh-*p*CH_3 (2412937).

Atom	Atom	Length/Å	Atom	Atom	Length/Å
Ag1	Ag1^1	2.9362(3)	C1	C6	1.391(13)
Ag1	Ag1^2	2.9362(3)	C2	C3	1.411(13)
Ag1	S1^2	2.638(3)	C6	C5	1.379(14)
Ag1	S1^3	2.485(3)	C3	C4	1.383(15)
Ag1	S1	2.452(3)	C5	C4	1.400(14)
S1	C1	1.774(9)	C4	C7	1.509(13)
C1	C2	1.397(13)			

^{1}1/2-X,-1/2+Y,+Z; ^{2}1/2-X,1/2+Y,+Z; ^{3}-1/2+X,+Y,3/2-Z

Table S40. Bond Angles for AgSPh-*p*CH_3 (2412937).

Atom	Atom	Atom	Angle/°	Atom	Atom	Atom	Angle/°
Ag1^1	Ag1	Ag1^2	157.77(5)	C1	S1	Ag1	102.4(3)
S1^1	Ag1	Ag1^1	51.85(6)	C1	S1	Ag1^2	132.1(3)
S1^3	Ag1	Ag1^2	76.19(6)	C1	S1	Ag1^4	113.7(3)
S1	Ag1	Ag1^1	102.21(7)	C2	C1	S1	118.8(7)
S1	Ag1	Ag1^2	57.79(6)	C6	C1	S1	121.5(7)
S1^1	Ag1	Ag1^2	146.51(6)	C6	C1	C2	119.7(9)
S1^3	Ag1	Ag1^1	119.72(6)	C1	C2	C3	118.4(9)
S1^3	Ag1	S1^1	98.20(6)	C5	C6	C1	121.1(9)
S1	Ag1	S1^3	132.23(11)	C4	C3	C2	122.0(9)
S1	Ag1	S1^1	126.84(10)	C6	C5	C4	120.5(9)
Ag1	S1	Ag1^4	98.99(9)	C3	C4	C5	118.4(9)
Ag1	S1	Ag1^2	70.37(6)	C3	C4	C7	121.4(9)
Ag1^4	S1	Ag1^2	114.27(9)	C5	C4	C7	120.3(10)

^{1}1/2-X,1/2+Y,+Z; ^{2}1/2-X,-1/2+Y,+Z; ^{3}-1/2+X,+Y,3/2-Z; ^{4}1/2+X,+Y,3/2-Z

Table S41. Torsion Angles for AgSPh-*p*CH_3 (2412937).

A	B	C	D	Angle/°	A	B	C	D	Angle/°
$Ag1^1$	S1	C1	C2	160.3(7)	C1	C2	C3	C4	0.5(15)
Ag1	S1	C1	C2	-93.9(7)	C1	C6	C5	C4	0.7(16)
$Ag1^2$	S1	C1	C2	-19.3(10)	C2	C1	C6	C5	-1.7(15)
$Ag1^2$	S1	C1	C6	162.3(6)	C2	C3	C4	C5	-1.6(15)
$Ag1^1$	S1	C1	C6	-18.1(9)	C2	C3	C4	C7	178.8(10)
Ag1	S1	C1	C6	87.7(8)	C6	C1	C2	C3	1.1(14)
S1	C1	C2	C3	-177.3(7)	C6	C5	C4	C3	1.0(15)
S1	C1	C6	C5	176.6(8)	C6	C5	C4	C7	-179.5(10)

^{1}1/2+X,+Y,3/2-Z; ^{2}1/2-X,-1/2+Y,+Z

Table S42. Hydrogen Atom Coordinates (Å×10^4) and Isotropic Displacement Parameters (Å^2×10^3) for AgSPh-*p*CH_3 (2412937).

Atom	*x*	*y*	*z*	U(eq)
H2	3605.52	2067.5	6306.93	23
H6	6231.18	7658.13	6778.17	25
H3	3788.33	3780.05	5646.19	28
H5	6442.48	9282.85	6124.65	28
H7A	6478.78	7335.17	5269.82	47
H7B	5192.11	9420.23	5411.47	47
H7C	4337.56	7123.69	5213.67	47

14.6 AgSPh-*p*F (CCDC-2412916)

Table S43. Fractional Atomic Coordinates (×10^4^) and Equivalent Isotropic Displacement Parameters ($Å^2 \times 10^3$) for AgSPh-*p*F (2412916). U_{eq} is defined as 1/3 of the trace of the orthogonalised U_{IJ} tensor.

**Atom**	***x***	***y***	***z***	**U(eq)**
Ag1	2622.7(11)	6664.8(9)	2602.1(2)	30.9(2)
S1	5035(3)	4098(2)	2910.9(6)	22.6(4)
F1	5156(10)	2884(8)	4897.1(15)	39.6(12)
C1	5045(12)	3807(9)	3516(2)	19.0(13)
C2	3248(13)	4397(10)	3782(3)	23.8(15)
C6	6916(13)	2930(10)	3714(3)	24.7(15)
C4	5113(14)	3193(11)	4442(2)	26.6(16)
C5	6956(14)	2604(12)	4185(3)	29.0(16)
C3	3281(14)	4084(11)	4252(3)	28.9(16)

Table S44. Anisotropic Displacement Parameters ($Å^2 \times 10^3$) for AgSPh-*p*F (2412916). The Anisotropic displacement factor exponent takes the form: $-2\pi^2[h^2a^{*2}U_{11}+2hka^*b^*U_{12}+\ldots]$.

**Atom**	**U_{11}**	**U_{22}**	**U_{33}**	**U_{23}**	**U_{13}**	**U_{12}**
Ag1	27.1(3)	19.5(3)	46.0(4)	2.1(2)	3.1(3)	1.7(2)
S1	28.1(9)	16.0(8)	23.9(8)	-0.1(6)	0.5(7)	-0.1(7)
F1	50(3)	46(3)	23(2)	5(2)	1(2)	3(3)
C1	21(3)	13(3)	22(3)	2(2)	3(3)	-5(3)
C2	23(3)	16(3)	32(4)	2(3)	1(3)	2(3)
C6	25(4)	17(3)	32(4)	-5(3)	2(3)	4(3)
C4	35(4)	24(4)	21(3)	2(3)	2(3)	-3(3)
C5	29(4)	26(4)	32(4)	5(3)	-4(3)	5(4)
C3	30(4)	26(4)	32(4)	-6(3)	5(3)	3(3)

Table S45. Bond Lengths for AgSPh-*p*F (2412916).

Atom	Atom	Length/Å	Atom	Atom	Length/Å
Ag1	$Ag1^{1}$	3.0018(3)	F1	C4	1.352(8)
Ag1	$Ag1^{2}$	3.0018(3)	C1	C2	1.380(10)
Ag1	$S1^{3}$	2.5215(19)	C1	C6	1.396(10)
Ag1	S1	2.5054(19)	C2	C3	1.394(11)
Ag1	$S1^{1}$	2.8318(19)	C6	C5	1.398(11)
Ag1	$S1^{4}$	2.6927(19)	C4	C5	1.387(11)
S1	C1	1.786(7)	C4	C3	1.373(11)

^{1}-1/2+X,+Y,1/2-Z; ^{2}1/2+X,+Y,1/2-Z; ^{3}1/2-X,1/2+Y,+Z; ^{4}1-X,1/2+Y,1/2-Z

Table S46. Bond Angles for AgSPh-*p*F (2412916).

Atom	Atom	Atom	Angle/°	Atom	Atom	Atom	Angle/°
Ag1^{1}	Ag1	Ag1^{2}	157.02(5)	Ag1^{5}	S1	Ag1^{1}	126.89(7)
S1^{2}	Ag1	Ag1^{2}	50.77(4)	Ag1^{6}	S1	Ag1^{1}	81.78(5)
S1^{3}	Ag1	Ag1^{1}	132.78(5)	Ag1	S1	Ag1^{6}	124.92(7)
S1^{4}	Ag1	Ag1^{2}	112.98(5)	C1	S1	Ag1^{1}	127.0(2)
S1	Ag1	Ag1^{1}	61.10(5)	C1	S1	Ag1^{6}	118.4(2)
S1^{2}	Ag1	Ag1^{1}	114.91(5)	C1	S1	Ag1^{5}	106.0(2)
S1^{4}	Ag1	Ag1^{1}	52.22(4)	C1	S1	Ag1	116.6(2)
S1	Ag1	Ag1^{2}	128.84(5)	C2	C1	S1	121.4(6)
S1^{3}	Ag1	Ag1^{2}	57.57(4)	C2	C1	C6	120.7(7)
S1	Ag1	S1^{2}	90.61(7)	C6	C1	S1	117.9(5)
S1	Ag1	S1^{3}	137.61(8)	C1	C2	C3	119.7(7)
S1^{3}	Ag1	S1^{2}	108.31(3)	C1	C6	C5	119.9(7)
S1	Ag1	S1^{4}	113.29(3)	F1	C4	C5	118.1(7)
S1^{3}	Ag1	S1^{4}	93.54(7)	F1	C4	C3	119.4(7)
S1^{4}	Ag1	S1^{2}	114.03(7)	C3	C4	C5	122.5(7)
Ag1	S1	Ag1^{1}	68.13(4)	C4	C5	C6	118.1(7)
Ag1	S1	Ag1^{5}	92.06(6)	C4	C3	C2	119.1(7)
Ag1^{5}	S1	Ag1^{6}	70.21(5)				

^{1}1/2+X,+Y,1/2-Z; ^{2}-1/2+X,+Y,1/2-Z; ^{3}1/2-X,1/2+Y,+Z; ^{4}1-X,1/2+Y,1/2-Z; ^{5}1/2-X,-1/2+Y,+Z; ^{6}1-X,-1/2+Y,1/2-Z

Table S47. Torsion Angles for AgSPh-*p*F (2412916).

A	B	C	D	Angle/°	A	B	C	D	Angle/°
Ag1	S1	C1	C2	24.9(7)	S1	C1	C6	C5	-177.4(6)
$Ag1^1$	S1	C1	C2	-151.6(5)	F1	C4	C5	C6	-179.7(7)
$Ag1^2$	S1	C1	C2	-75.8(6)	F1	C4	C3	C2	-180.0(7)
$Ag1^3$	S1	C1	C2	106.6(6)	C1	C2	C3	C4	0.1(12)
$Ag1^2$	S1	C1	C6	103.1(5)	C1	C6	C5	C4	-0.7(12)
Ag1	S1	C1	C6	-156.3(5)	C2	C1	C6	C5	1.5(11)
$Ag1^3$	S1	C1	C6	-74.5(6)	C6	C1	C2	C3	-1.2(11)
$Ag1^1$	S1	C1	C6	27.2(6)	C5	C4	C3	C2	0.7(12)
S1	C1	C2	C3	177.6(6)	C3	C4	C5	C6	-0.4(12)

^{1}1-X,-1/2+Y,1/2-Z; ^{2}1/2-X,-1/2+Y,+Z; ^{3}1/2+X,+Y,1/2-Z

Table S48. Hydrogen Atom Coordinates (Å×10^4) and Isotropic Displacement Parameters (Å^2×10^3) for AgSPh-*p*F (2412916).

Atom	*x*	*y*	*z*	U(eq)
H2	1995.17	5013.87	3645.6	29
H6	8160.54	2555.91	3529.14	30
H5	8207.87	1996.37	4324.05	35
H3	2052.46	4481.58	4438.08	35

14.7 AgSPh-Cl_2(2,3) (CCDC-2412923)

Table S49. Fractional Atomic Coordinates (×10^4) and Equivalent Isotropic Displacement Parameters ($Å^2$×10^3) for AgSPh-Cl_2(2,3) (2412923). U_{eq} is defined as 1/3 of the trace of the orthogonalised U_{IJ} tensor.

Atom	*x*	*y*	*z*	U(eq)
Ag1	-395(2)	5756.4(13)	5004.6(6)	33.4(2)
Cl1	5579(7)	7467(4)	6917(2)	35.1(6)
S1	3655(5)	3468(4)	5725.8(13)	19.5(4)
Cl2	4105(9)	7718(7)	9084(2)	73.3(15)
C6	3550(20)	5470(20)	7451(6)	25(2)
C1	2560(20)	3720(14)	6930(6)	23(2)
C2	820(20)	2188(16)	7382(7)	28(2)
C5	2790(20)	5620(30)	8410(6)	40(3)
C4	1090(30)	4080(30)	8840(7)	51(4)
C3	-10(30)	2360(20)	8329(7)	39(3)

Table S50. Anisotropic Displacement Parameters ($Å^2$×10^3) for AgSPh-Cl_2(2,3) (2412923). The Anisotropic displacement factor exponent takes the form: $-2\pi^2[h^2a^{*2}U_{11}+2hka^*b^*U_{12}+\ldots]$.

Atom	U_{11}	U_{22}	U_{33}	U_{23}	U_{13}	U_{12}
Ag1	45.5(5)	26.6(3)	27.3(3)	0.0(3)	-12.2(3)	8.8(5)
Cl1	28.8(15)	28.3(12)	48.6(15)	-13.3(11)	8.6(11)	-8.5(11)
S1	18.0(12)	22.3(11)	18.3(8)	-1.3(9)	1.8(8)	-0.6(9)
Cl2	35(2)	131(4)	54.1(18)	-69(2)	1.8(14)	-2.6(19)
C6	16(4)	37(6)	23(4)	-9(5)	1(3)	-2(4)
C1	22(5)	26(6)	20(4)	4(3)	-2(3)	7(4)
C2	19(5)	30(5)	36(5)	13(4)	-4(4)	-4(4)
C5	27(5)	69(7)	23(4)	-20(7)	-2(4)	2(8)
C4	35(7)	102(12)	15(5)	6(6)	-2(4)	9(7)
C3	22(6)	66(8)	28(5)	24(6)	3(4)	-8(6)

Table S51. Bond Lengths for AgSPh-Cl_2(2,3) (2412923).

Atom	Atom	Length/Å	Atom	Atom	Length/Å
Ag1	Ag1[1]	3.2564(10)	C6	C1	1.403(15)
Ag1	Ag1[2]	3.2565(10)	C6	C5	1.394(12)
Ag1	S1[1]	2.411(3)	C1	C2	1.383(13)
Ag1	S1	2.415(3)	C2	C3	1.386(15)
Cl1	C6	1.720(12)	C5	C4	1.37(2)
S1	C1	1.770(9)	C4	C3	1.392(19)
Cl2	C5	1.732(15)			

[1]-X,1/2+Y,1-Z; [2]-X,-1/2+Y,1-Z

Table S52. Bond Angles for AgSPh-Cl_2(2,3) (2412923).

Atom	Atom	Atom	Angle/°	Atom	Atom	Atom	Angle/°
Ag1[1]	Ag1	Ag1[2]	168.69(6)	C5	C6	C1	119.6(12)
S1	Ag1	Ag1[2]	47.52(6)	C6	C1	S1	119.9(7)
S1[1]	Ag1	Ag1[1]	47.61(6)	C2	C1	S1	121.3(8)
S1	Ag1	Ag1[1]	123.16(8)	C2	C1	C6	118.8(9)
S1[1]	Ag1	Ag1[2]	140.93(7)	C1	C2	C3	121.6(10)
S1[1]	Ag1	S1	169.96(10)	C6	C5	Cl2	120.7(12)
Ag1[2]	S1	Ag1	84.87(7)	C4	C5	Cl2	118.9(8)
C1	S1	Ag1	99.1(3)	C4	C5	C6	120.3(13)
C1	S1	Ag1[2]	108.9(3)	C5	C4	C3	121.0(10)
C1	C6	Cl1	120.9(7)	C2	C3	C4	118.6(10)
C5	C6	Cl1	119.4(11)				

[1]-X,1/2+Y,1-Z; [2]-X,-1/2+Y,1-Z

Table S53. Torsion Angles for AgSPh-Cl_2(2,3) (2412923).

A	B	C	D	Angle/°	A	B	C	D	Angle/°
Ag1	S1	C1	C6	-78.2(8)	Cl2	C5	C4	C3	-179.7(10)
Ag1[1]	S1	C1	C6	-165.9(7)	C6	C1	C2	C3	1.2(15)
Ag1[1]	S1	C1	C2	16.3(9)	C6	C5	C4	C3	-1.9(19)
Ag1	S1	C1	C2	103.9(8)	C1	C6	C5	Cl2	177.7(9)
Cl1	C6	C1	S1	4.5(12)	C1	C6	C5	C4	-0.2(17)
Cl1	C6	C1	C2	-177.7(8)	C1	C2	C3	C4	-3.1(17)
Cl1	C6	C5	Cl2	-4.1(14)	C5	C6	C1	S1	-177.4(8)
Cl1	C6	C5	C4	178.0(10)	C5	C6	C1	C2	0.5(15)
S1	C1	C2	C3	179.0(8)	C5	C4	C3	C2	3.5(19)

[1]-X,-1/2+Y,1-Z

Table S54. Hydrogen Atom Coordinates (Å×10^4) and Isotropic Displacement Parameters (Å^2×10^3) for AgSPh-Cl_2(2,3) (2412923).

Atom	*x*	*y*	*z*	U(eq)
H2	180.51	987.6	7035.79	34
H4	646.16	4180.84	9498.35	61
H3	-1292.05	1327.88	8621.56	46

14.8 AgSPh-Cl_2(2,6) (CCDC-2412941)

Table S55. Fractional Atomic Coordinates (×10^4) and Equivalent Isotropic Displacement Parameters (Å^2×10^3) for AgSPh-Cl2(2,6) (2412941). U_{eq} is defined as 1/3 of the trace of the orthogonalised U_{IJ} tensor.

**Atom**	***x***	***y***	***z***	**U(eq)**
Ag1	5208.6(5)	4192.7(3)	3672(2)	25.6(2)
Ag2	3459.2(5)	4874.4(3)	5890(2)	24.9(2)
Cl2	6639.7(16)	3412.7(8)	-1079(7)	24.8(5)
S1	6699.3(15)	4552.5(8)	1341(7)	21.3(5)
Cl3	1854.1(16)	4170.1(9)	10547(6)	24.7(5)
Cl1	8682.9(16)	4873.2(9)	5345(6)	25.4(5)
S2	4091.4(16)	4091.0(9)	8178(6)	21.8(5)
Cl4	4643.3(16)	3071.0(8)	4155(7)	26.6(5)
C2	8547(6)	4270(3)	3780(20)	20.4(17)
C1	7692(7)	4136(4)	2120(20)	21.9(19)
C7	3217(7)	3616(3)	7270(20)	22(2)
C6	7668(6)	3635(3)	1010(30)	21.6(18)
C3	9344(7)	3948(3)	4290(30)	21.7(19)
C11	2755(8)	2800(4)	5000(30)	27(2)
C9	1532(7)	3276(4)	7820(20)	25(2)
C8	2233(6)	3649(4)	8440(30)	23(2)
C12	3451(7)	3172(3)	5620(20)	20.9(18)
C5	8442(7)	3299(4)	1520(30)	24.8(19)
C10	1797(7)	2854(4)	6060(30)	27(2)
C4	9273(7)	3456(4)	3160(30)	27(2)

Table S56. Anisotropic Displacement Parameters (Å^2×10^3) for AgSPh-Cl2(2,6) (2412941). The Anisotropic displacement factor exponent takes the form: $-2\pi^2[h^2a^{*2}U_{11}+2hka^*b^*U_{12}+\ldots]$.

**Atom**	**U11**	**U22**	**U33**	**U23**	**U13**	**U12**
Ag1	18.8(3)	23.7(3)	34.4(4)	1.9(3)	4.6(3)	-0.2(3)
Ag2	19.0(3)	22.1(3)	33.5(4)	2.2(3)	-0.7(3)	0.4(3)
Cl2	20.6(10)	24.2(10)	29.6(12)	-3.4(10)	-3.5(11)	-0.7(8)
S1	16.3(10)	19.3(9)	28.4(13)	2.4(9)	-0.7(10)	-0.1(8)
Cl3	20.3(10)	23.0(10)	30.7(13)	-0.6(10)	5.1(9)	0.8(9)
Cl1	17.8(10)	21.9(10)	36.5(14)	-2.0(10)	-2.6(9)	-1.9(9)
S2	16.1(10)	22.6(10)	26.7(13)	-0.5(9)	-1.0(8)	-1.9(8)
Cl4	19.2(10)	24.7(10)	35.9(14)	-1.6(10)	2.2(11)	3.8(8)
C2	16(4)	23(4)	22(5)	2(4)	5(4)	-3(3)
C1	23(4)	21(4)	22(5)	3(4)	4(4)	-4(4)
C7	24(5)	21(4)	20(5)	7(4)	-4(4)	-4(4)
C6	15(4)	27(4)	23(5)	-1(4)	4(4)	-1(3)
C3	20(4)	20(4)	25(5)	3(4)	6(4)	0(3)
C11	34(5)	21(5)	27(6)	-2(4)	-5(4)	3(4)
C9	16(4)	26(5)	32(5)	10(4)	-1(4)	-4(4)
C8	14(4)	27(5)	29(6)	4(4)	4(4)	-1(3)
C12	19(4)	18(4)	26(5)	5(4)	0(4)	-1(3)
C5	25(4)	28(5)	22(5)	0(4)	7(4)	0(4)
C10	18(4)	25(4)	37(6)	-1(5)	4(5)	-8(3)
C4	18(4)	29(5)	32(6)	3(4)	2(4)	7(4)

Table S57. Bond Lengths for AgSPh-Cl2(2,6) (2412941).

Atom	Atom	Length/Å	Atom	Atom	Length/Å
Ag1	Ag2[1]	3.2002(10)	Cl4	C12	1.744(9)
Ag1	Ag2	3.1179(10)	C2	C1	1.395(13)
Ag1	S1	2.433(2)	C2	C3	1.392(12)
Ag1	S2	2.432(3)	C1	C6	1.410(13)
Ag1	S2[2]	2.766(3)	C7	C8	1.418(13)
Ag2	S1[3]	2.754(3)	C7	C12	1.402(14)
Ag2	S1[1]	2.449(3)	C6	C5	1.389(13)
Ag2	S2	2.445(2)	C3	C4	1.394(13)
Cl2	C6	1.744(10)	C11	C12	1.389(14)
S1	C1	1.769(10)	C11	C10	1.375(14)
Cl3	C8	1.720(10)	C9	C8	1.395(13)
Cl1	C2	1.740(9)	C9	C10	1.390(15)
S2	C7	1.769(10)	C5	C4	1.381(14)

[1]1-X,1-Y,+Z; [2]+X,+Y,-1+Z; [3]1-X,1-Y,1+Z

Table S58. Bond Angles for AgSPh-Cl2(2,6) (2412941).

**Atom**	**Atom**	**Atom**	**Angle/°**	**Atom**	**Atom**	**Atom**	**Angle/°**
Ag2	Ag1	Ag2^{1}	83.65(3)	Ag2	S2	Ag1^{5}	115.70(9)
S1	Ag1	Ag2^{1}	49.26(6)	C7	S2	Ag1^{5}	127.7(3)
S1	Ag1	Ag2	121.21(6)	C7	S2	Ag1	109.1(4)
S1	Ag1	S2^{2}	98.94(8)	C7	S2	Ag2	106.9(3)
S2	Ag1	Ag2	50.45(6)	C1	C2	Cl1	120.8(7)
S2^{2}	Ag1	Ag2^{1}	128.61(6)	C3	C2	Cl1	115.3(7)
S2	Ag1	Ag2^{1}	102.07(6)	C3	C2	C1	123.9(9)
S2^{2}	Ag1	Ag2	83.79(5)	C2	C1	S1	123.9(7)
S2	Ag1	S1	150.29(9)	C2	C1	C6	115.2(9)
S2	Ag1	S2^{2}	107.29(8)	C6	C1	S1	120.9(7)
Ag1	Ag2	Ag1^{1}	86.41(3)	C8	C7	S2	120.4(8)
S1^{3}	Ag2	Ag1^{1}	81.74(5)	C12	C7	S2	123.7(8)
S1^{1}	Ag2	Ag1^{1}	48.82(5)	C12	C7	C8	115.7(9)
S1^{1}	Ag2	Ag1	101.26(6)	C1	C6	Cl2	120.1(7)
S1^{3}	Ag2	Ag1	128.85(5)	C5	C6	Cl2	117.3(7)
S1^{1}	Ag2	S1^{3}	107.16(8)	C5	C6	C1	122.6(9)
S2	Ag2	Ag1	50.07(6)	C2	C3	C4	118.2(9)
S2	Ag2	Ag1^{1}	125.23(6)	C10	C11	C12	120.2(10)
S2	Ag2	S1^{3}	99.93(8)	C10	C9	C8	119.9(9)
S2	Ag2	S1^{1}	149.87(9)	C7	C8	Cl3	120.4(7)
Ag1	S1	Ag2^{4}	119.08(9)	C9	C8	Cl3	117.7(7)
Ag1	S1	Ag2^{1}	81.93(8)	C9	C8	C7	121.8(10)
Ag2^{1}	S1	Ag2^{4}	107.17(8)	C7	C12	Cl4	120.8(7)
C1	S1	Ag1	107.9(3)	C11	C12	Cl4	116.6(7)
C1	S1	Ag2^{4}	123.9(3)	C11	C12	C7	122.5(9)
C1	S1	Ag2^{1}	108.2(3)	C4	C5	C6	119.6(9)
Ag1	S2	Ag1^{5}	107.29(8)	C11	C10	C9	119.7(9)
Ag1	S2	Ag2	79.48(8)	C5	C4	C3	120.5(9)

^{1}1-X,1-Y,+Z; ^{2}+X,+Y,-1+Z; ^{3}1-X,1-Y,1+Z; ^{4}1-X,1-Y,-1+Z; ^{5}+X,+Y,1+Z

Table S59. Torsion Angles for AgSPh-Cl2(2,6) (2412941).

A	B	C	D	Angle/˚	A	B	C	D	Angle/˚
Ag1	S1	C1	C2	-121.7(8)	S2	C7	C12	Cl4	-3.0(12)
Ag1	S1	C1	C6	59.5(9)	S2	C7	C12	C11	178.6(8)
Ag1[1]	S2	C7	C8	82.3(9)	C2	C1	C6	Cl2	179.5(7)
Ag1	S2	C7	C8	-146.0(7)	C2	C1	C6	C5	-0.3(15)
Ag1[1]	S2	C7	C12	-92.7(9)	C2	C3	C4	C5	1.7(15)
Ag1	S2	C7	C12	39.0(9)	C1	C2	C3	C4	-2.1(15)
Ag2[2]	S1	C1	C2	92.1(9)	C1	C6	C5	C4	-0.1(16)
Ag2[3]	S1	C1	C2	-34.5(9)	C6	C5	C4	C3	-0.6(16)
Ag2[2]	S1	C1	C6	-86.8(8)	C3	C2	C1	S1	-177.5(8)
Ag2[3]	S1	C1	C6	146.6(7)	C3	C2	C1	C6	1.4(14)
Ag2	S2	C7	C8	-61.6(9)	C8	C7	C12	Cl4	-178.2(7)
Ag2	S2	C7	C12	123.5(8)	C8	C7	C12	C11	3.5(14)
Cl2	C6	C5	C4	-179.9(8)	C8	C9	C10	C11	1.3(17)
S1	C1	C6	Cl2	-1.5(12)	C12	C7	C8	Cl3	179.2(7)
S1	C1	C6	C5	178.7(8)	C12	C7	C8	C9	-3.4(15)
Cl1	C2	C1	S1	1.9(12)	C12	C11	C10	C9	-1.3(17)
Cl1	C2	C1	C6	-179.2(7)	C10	C11	C12	Cl4	-179.6(9)
Cl1	C2	C3	C4	178.4(8)	C10	C11	C12	C7	-1.2(16)
S2	C7	C8	Cl3	3.8(12)	C10	C9	C8	Cl3	178.6(9)
S2	C7	C8	C9	-178.8(8)	C10	C9	C8	C7	1.2(16)

[1]+X,+Y,1+Z; [2]1-X,1-Y,-1+Z; [3]1-X,1-Y,+Z

Table S60. Hydrogen Atom Coordinates (Å×10^4) and Isotropic Displacement Parameters (Å^2×10^3) for AgSPh-Cl2(2,6) (2412941).

Atom	*x*	*y*	*z*	U(eq)
H3	9920.61	4060.43	5372.76	26
H11	2941.38	2508.35	3838.96	32
H9	874.76	3311.43	8593.02	30
H5	8399.31	2964.24	738.83	30
H10	1317.93	2602.06	5597.11	33
H4	9799.63	3225.79	3533.44	32

14.9 AgSPh-Me2(2,6) (CCDC-2412942)

Table S61. Fractional Atomic Coordinates ($\times10^4$) and Equivalent Isotropic Displacement Parameters ($Å^2\times10^3$) for AgSPh-Me2(2,6) (2412942). U_{eq} is defined as 1/3 of the trace of the orthogonalised U_{IJ} tensor.

**Atom**	***x***	***y***	***z***	**U(eq)**
Ag1	5237.4(2)	1502.3(5)	3195.6(2)	15.68(7)
Ag2	3483.9(2)	4784.7(5)	2479.2(2)	16.06(7)
S1	4326.7(5)	6319.0(17)	3320.3(3)	12.95(14)
S2	3053.6(5)	16.5(17)	2020.6(3)	12.84(14)
C1	3499(2)	6032(7)	3780.3(10)	13.0(5)
C6	3844(2)	4425(7)	4227.6(11)	15.0(6)
C7	2150(2)	9307(8)	3245.7(12)	18.5(6)
C9	2040(2)	606(7)	1503.0(11)	13.9(6)
C2	2552(2)	7571(7)	3713.4(11)	16.4(6)
C16	995(2)	3591(8)	2060.5(12)	21.9(7)
C5	3209(3)	4372(8)	4589.8(11)	19.4(6)
C8	4854(2)	2745(8)	4332.7(11)	19.3(6)
C14	1162(2)	2316(7)	1568.4(11)	17.1(6)
C10	2117(2)	-686(7)	1040.1(11)	16.3(6)
C3	1955(2)	7485(8)	4089.9(12)	19.3(6)
C15	2995(2)	-2708(8)	963.9(11)	18.0(6)
C4	2274(3)	5890(8)	4525.6(12)	22.0(7)
C13	409(2)	2847(8)	1154.3(12)	21.5(7)
C11	1337(3)	-76(8)	637.1(12)	21.0(7)
C12	498(3)	1718(9)	691.1(13)	24.6(7)

Table S62. Anisotropic Displacement Parameters ($Å^2 \times 10^3$) for AgSPh-Me_2(2,6) (2412942). The Anisotropic displacement factor exponent takes the form: $-2\pi^2[h^2a^{*2}U_{11}+2hka^*b^*U_{12}+\ldots]$.

**Atom**	U_{11}	U_{22}	U_{33}	U_{23}	U_{13}	U_{12}
Ag1	16.69(11)	16.49(12)	13.84(11)	0.76(8)	2.39(8)	1.84(9)
Ag2	17.91(12)	16.88(12)	13.16(11)	-2.66(8)	1.81(8)	-0.39(9)
S1	14.5(3)	13.9(3)	10.7(3)	0.3(3)	2.7(2)	-0.4(3)
S2	13.9(3)	14.6(3)	9.3(3)	0.3(3)	-0.3(2)	-0.1(3)
C1	15.9(14)	12.3(13)	11.0(13)	-1.3(11)	2.7(10)	-2.2(11)
C6	19.6(14)	14.7(14)	9.9(13)	-2.4(11)	0.2(11)	-3.0(12)
C7	15.0(14)	21.6(16)	18.7(15)	2.6(12)	2.1(12)	3.7(12)
C9	16.0(14)	14.6(14)	11.0(13)	1.7(11)	2.1(11)	-3.1(11)
C2	17.5(14)	16.3(15)	15.1(14)	-4.0(12)	2.1(11)	-2.8(12)
C16	16.9(15)	26.3(18)	22.7(16)	-2.4(14)	3.8(12)	1.1(13)
C5	25.0(16)	21.6(16)	12.1(14)	0.0(12)	4.9(12)	-4.2(13)
C8	20.5(15)	21.4(16)	14.9(14)	4.1(12)	-0.4(12)	-0.1(13)
C14	15.0(14)	16.7(15)	18.5(15)	2.8(12)	-0.5(11)	-1.0(12)
C10	18.4(14)	15.4(15)	14.2(14)	1.7(11)	0.2(11)	-4.8(12)
C3	18.2(15)	20.4(16)	20.3(15)	-4.8(13)	6.2(12)	-2.6(13)
C15	18.2(14)	23.2(16)	12.6(14)	-2.5(12)	2.5(11)	-0.4(13)
C4	25.8(17)	25.2(17)	16.8(15)	-2.4(13)	8.8(13)	-5.2(14)
C13	14.2(14)	23.0(17)	25.4(16)	2.4(14)	-2.5(12)	-0.3(13)
C11	23.6(16)	23.6(17)	14.0(14)	-0.3(13)	-2.2(12)	-6.4(13)
C12	18.3(15)	30.0(19)	22.6(16)	3.8(14)	-5.6(12)	-1.2(14)

Table S63. Bond Lengths for AgSPh-Me_2(2,6) (2412942).

Atom	Atom	Length/Å	Atom	Atom	Length/Å
Ag1	Ag2[1]	3.0511(3)	C6	C8	1.506(4)
Ag1	Ag2	3.1206(3)	C7	C2	1.505(4)
Ag1	S1[2]	2.6097(8)	C9	C14	1.413(4)
Ag1	S1	2.4721(8)	C9	C10	1.403(4)
Ag1	S2[1]	2.5048(7)	C2	C3	1.394(4)
Ag2	S1	2.4750(7)	C16	C14	1.504(4)
Ag2	S2[3]	2.6222(8)	C5	C4	1.384(5)
Ag2	S2	2.4479(8)	C14	C13	1.396(4)
S1	C1	1.799(3)	C10	C15	1.497(4)
S2	C9	1.797(3)	C10	C11	1.402(4)
C1	C6	1.418(4)	C3	C4	1.384(5)
C1	C2	1.402(4)	C13	C12	1.381(5)
C6	C5	1.396(4)	C11	C12	1.383(5)

[1]1-X,+Y,1/2-Z; [2]+X,-1+Y,+Z; [3]+X,1+Y,+Z

Table S64. Bond Angles for AgSPh-Me_2(2,6) (2412942).

Atom	Atom	Atom	Angle/°	Atom	Atom	Atom	Angle/°
Ag2[1]	Ag1	Ag2	80.245(9)	Ag2	S2	Ag2[2]	118.84(3)
S1	Ag1	Ag2	50.932(17)	C9	S2	Ag1[1]	110.96(10)
S1	Ag1	Ag2[1]	90.703(18)	C9	S2	Ag2	111.16(10)
S1[2]	Ag1	Ag2[1]	142.879(18)	C9	S2	Ag2[2]	125.12(10)
S1[2]	Ag1	Ag2	99.859(18)	C6	C1	S1	119.4(2)
S1	Ag1	S1[2]	118.41(3)	C2	C1	S1	120.3(2)
S1	Ag1	S2[1]	136.72(3)	C2	C1	C6	120.1(3)
S2[1]	Ag1	Ag2	124.929(18)	C1	C6	C8	123.6(3)
S2[1]	Ag1	Ag2[1]	51.135(18)	C5	C6	C1	118.3(3)
S2[1]	Ag1	S1[2]	104.86(2)	C5	C6	C8	118.0(3)
Ag1[1]	Ag2	Ag1	74.816(9)	C14	C9	S2	119.6(2)
S1	Ag2	Ag1[1]	118.694(19)	C10	C9	S2	119.9(2)
S1	Ag2	Ag1	50.851(18)	C10	C9	C14	120.5(3)
S1	Ag2	S2[3]	103.70(2)	C1	C2	C7	122.2(3)
S2	Ag2	Ag1	90.919(19)	C3	C2	C1	119.2(3)
S2[3]	Ag2	Ag1	141.219(18)	C3	C2	C7	118.5(3)
S2[3]	Ag2	Ag1[1]	102.580(18)	C4	C5	C6	121.7(3)
S2	Ag2	Ag1[1]	52.819(18)	C9	C14	C16	123.5(3)
S2	Ag2	S1	137.43(3)	C13	C14	C9	118.2(3)
S2	Ag2	S2[3]	118.84(3)	C13	C14	C16	118.3(3)
Ag1	S1	Ag1[3]	118.41(3)	C9	C10	C15	122.3(3)
Ag1	S1	Ag2	78.22(2)	C11	C10	C9	118.7(3)
Ag2	S1	Ag1[3]	105.03(3)	C11	C10	C15	119.1(3)
C1	S1	Ag1	113.63(10)	C4	C3	C2	121.3(3)
C1	S1	Ag1[3]	119.74(10)	C3	C4	C5	119.2(3)
C1	S1	Ag2	113.51(10)	C12	C13	C14	121.8(3)
Ag1[1]	S2	Ag2[2]	101.89(3)	C12	C11	C10	121.3(3)
Ag2	S2	Ag1[1]	76.05(2)	C13	C12	C11	119.3(3)

[1]1-X,+Y,1/2-Z; [2]+X,-1+Y,+Z; [3]+X,1+Y,+Z

Table S65. Torsion Angles for AgSPh-Me$_2$(2,6) (2412942).

**A**	**B**	**C**	**D**	**Angle/°**	**A**	**B**	**C**	**D**	**Angle/°**
Ag1	S1	C1	C6	42.0(3)	C1	C6	C5	C4	-1.4(5)
Ag1[1]	S1	C1	C6	-106.0(2)	C1	C2	C3	C4	-0.6(5)
Ag1[1]	S1	C1	C2	69.4(3)	C6	C1	C2	C7	-179.9(3)
Ag1	S1	C1	C2	-142.6(2)	C6	C1	C2	C3	-0.3(5)
Ag1[2]	S2	C9	C14	-128.2(2)	C6	C5	C4	C3	0.5(5)
Ag1[2]	S2	C9	C10	53.1(3)	C7	C2	C3	C4	179.0(3)
Ag2	S1	C1	C6	128.9(2)	C9	C14	C13	C12	0.8(5)
Ag2	S1	C1	C2	-55.7(3)	C9	C10	C11	C12	-1.0(5)
Ag2	S2	C9	C14	-45.6(3)	C2	C1	C6	C5	1.3(4)
Ag2[3]	S2	C9	C14	109.1(2)	C2	C1	C6	C8	-179.3(3)
Ag2	S2	C9	C10	135.7(2)	C2	C3	C4	C5	0.5(5)
Ag2[3]	S2	C9	C10	-69.6(3)	C16	C14	C13	C12	-179.1(3)
S1	C1	C6	C5	176.7(2)	C8	C6	C5	C4	179.2(3)
S1	C1	C6	C8	-3.9(4)	C14	C9	C10	C15	-175.3(3)
S1	C1	C2	C7	4.7(4)	C14	C9	C10	C11	4.1(5)
S1	C1	C2	C3	-175.7(2)	C14	C13	C12	C11	2.2(5)
S2	C9	C14	C16	-2.9(4)	C10	C9	C14	C16	175.8(3)
S2	C9	C14	C13	177.3(2)	C10	C9	C14	C13	-4.0(5)
S2	C9	C10	C15	3.4(4)	C10	C11	C12	C13	-2.2(5)
S2	C9	C10	C11	-177.2(2)	C15	C10	C11	C12	178.4(3)

[1]+X,1+Y,+Z; [2]1-X,+Y,1/2-Z; [3]+X,-1+Y,+Z

Table S66. Hydrogen Atom Coordinates (Å×10^4) and Isotropic Displacement Parameters (Å^2×10^3) for AgSPh-Me$_2$(2,6) (2412942).

**Atom**	***x***	***y***	***z***	**U(eq)**
H7A	2665.71	10804.77	3180.25	28
H7B	1514.26	10374.36	3285.22	28
H7C	2009.41	7872.2	2966.95	28
H16A	1145.74	2006.24	2316.04	33
H16B	277.16	4249.67	2037.85	33
H16C	1451.62	5347.01	2148.02	33
H5	3424.41	3265.41	4888.07	23
H8A	4799.03	811.44	4148.07	29
H8B	5024.5	2319.39	4688.86	29
H8C	5395.79	4010.75	4229.51	29
H3	1315.73	8542.46	4046.42	23
H15A	3593.64	-1443.01	929.28	27
H15B	2797.34	-3931.6	662.59	27
H15C	3169.19	-4075.03	1249.89	27
H4	1856.1	5837.15	4777.79	26
H13	-181.4	4016.54	1192.27	26
H11	1387.28	-911.91	320.92	25
H12	-12.51	2168.89	412.41	30

14.10 AgSPy (CCDC-2412929)

Table S67. Fractional Atomic Coordinates ($\times10^4$) and Equivalent Isotropic Displacement Parameters ($Å^2\times10^3$) for AgSPy (2412929). U_{eq} is defined as 1/3 of the trace of the orthogonalised U_{IJ} tensor.

**Atom**	***x***	***y***	***z***	**U(eq)**
Ag3	9936.9(2)	-8896.7(2)	-4065.7(2)	14.28(3)
Ag2	6452.6(2)	-9110.3(2)	-4596.3(2)	12.81(3)
Ag1	9291.1(2)	-8167.7(2)	-5454.7(2)	17.18(4)
S3	9532.1(5)	-6741.9(4)	-4523.7(2)	10.17(7)
S1	6934.7(5)	-10284.9(4)	-5706.4(2)	10.63(8)
S2	7714.1(5)	-11206.8(4)	-4137.1(2)	11.34(8)
N3	6073.7(19)	-6988.0(15)	-4367.9(7)	11.6(3)
N2	9242.0(19)	-10066.8(15)	-3158.4(7)	11.7(3)
N1	7496(2)	-8159.9(15)	-6356.5(8)	14.3(3)
C12	7207(2)	-4866.5(17)	-4252.6(9)	12.6(3)
C15	4481(2)	-6499.1(18)	-4276.8(9)	13.5(3)
C14	4158(2)	-5214.6(18)	-4177.8(9)	14.0(3)
C11	7431(2)	-6181.8(17)	-4363.5(8)	9.7(3)
C10	9754(2)	-9984.5(18)	-2546.9(9)	14.1(3)
C13	5558(2)	-4380.4(18)	-4164.4(9)	14.0(3)
C1	6748(2)	-9317.3(17)	-6387.5(8)	11.4(3)
C2	5963(2)	-9763(2)	-6944.0(9)	17.1(4)
C9	9308(3)	-10864.0(19)	-2084.2(9)	16.6(3)
C6	8277(2)	-11079.4(17)	-3331.3(8)	11.0(3)
C5	7493(3)	-7424(2)	-6882.8(10)	20.4(4)
C7	7769(2)	-12016.2(18)	-2887.1(9)	13.9(3)
C8	8286(2)	-11898.3(19)	-2259.9(9)	16.1(3)
C4	6797(3)	-7806(2)	-7454.0(10)	24.6(4)
C3	6015(3)	-9000(2)	-7485.2(10)	23.6(4)

Table S68. Anisotropic Displacement Parameters (Å^2×10^3) for AgSPy (2412929). The Anisotropic displacement factor exponent takes the form: $-2\pi^2[h^2a^{*2}U_{11}+2hka^*b^*U_{12}+\ldots]$.

**Atom**	**U_{11}**	**U_{22}**	**U_{33}**	**U_{23}**	**U_{13}**	**U_{12}**
Ag3	11.00(6)	13.66(6)	18.17(7)	6.62(5)	0.11(4)	0.62(4)
Ag2	10.80(6)	9.12(6)	18.52(7)	-0.34(5)	0.71(4)	-0.11(4)
Ag1	14.07(6)	24.60(8)	12.89(7)	-6.63(5)	1.85(5)	-2.02(5)
S3	9.10(16)	9.30(17)	12.11(18)	-0.49(14)	0.21(13)	-0.45(13)
S1	9.16(16)	10.68(18)	12.06(18)	1.16(15)	0.16(13)	0.18(14)
S2	11.73(17)	10.78(18)	11.50(18)	1.13(15)	-0.16(14)	1.42(14)
N3	10.6(6)	10.5(7)	13.7(7)	0.6(5)	0.7(5)	0.0(5)
N2	12.1(6)	11.3(7)	11.9(7)	0.3(5)	2.0(5)	1.7(5)
N1	14.3(7)	11.6(7)	16.9(7)	1.6(6)	4.4(5)	1.1(5)
C12	14.1(7)	10.0(7)	13.7(8)	-1.2(6)	-0.1(6)	-0.7(6)
C15	10.8(7)	13.2(8)	16.6(8)	-0.2(6)	2.2(6)	-0.7(6)
C14	12.1(7)	14.4(8)	15.6(8)	-0.1(7)	2.0(6)	1.9(6)
C11	10.5(7)	10.4(7)	8.1(7)	0.5(6)	0.3(5)	0.9(6)
C10	14.5(7)	14.3(8)	13.6(8)	-2.2(6)	0.1(6)	1.7(6)
C13	16.5(8)	10.7(8)	14.6(8)	-1.1(6)	0.2(6)	2.0(6)
C1	8.6(6)	13.0(8)	12.6(8)	1.0(6)	1.6(5)	2.7(6)
C2	12.0(7)	24.4(10)	14.8(8)	-2.3(7)	-1.7(6)	1.7(7)
C9	19.4(8)	18.4(9)	12.0(8)	-0.2(7)	1.1(6)	5.6(7)
C6	9.4(6)	11.8(7)	11.8(8)	1.5(6)	1.3(5)	3.6(6)
C5	19.9(9)	16.3(9)	25.1(10)	7.3(8)	8.5(7)	5.2(7)
C7	13.3(7)	12.9(8)	15.7(8)	3.3(6)	2.0(6)	1.1(6)
C8	18.5(8)	16.0(9)	13.9(8)	5.8(7)	3.9(6)	4.0(7)
C4	21.6(9)	32.5(12)	19.8(10)	13.0(9)	6.3(7)	12.7(8)
C3	16.8(8)	39.6(13)	14.4(9)	0.3(8)	-1.9(7)	10.0(8)

Table S69. Bond Lengths for AgSPy (2412929).

Atom	Atom	Length/Å	Atom	Atom	Length/Å
Ag3	Ag2	2.9225(2)	N3	C15	1.350(2)
Ag3	Ag1	3.0495(2)	N3	C11	1.348(2)
Ag3	Ag1^{1}	3.2864(3)	N2	C10	1.343(2)
Ag3	S3	2.4689(5)	N2	C6	1.345(2)
Ag3	S1^{1}	2.6173(4)	N1	C1	1.344(2)
Ag3	N2	2.3280(15)	N1	C5	1.346(3)
Ag2	Ag2^{2}	3.3693(3)	C12	C11	1.406(2)
Ag2	Ag1	3.0160(2)	C12	C13	1.389(2)
Ag2	S1	2.6605(5)	C15	C14	1.382(3)
Ag2	S1^{2}	2.7769(4)	C14	C13	1.392(3)
Ag2	S2	2.5843(5)	C10	C9	1.383(3)
Ag2	N3	2.2898(15)	C1	C2	1.393(3)
Ag1	S3	2.4629(5)	C2	C3	1.388(3)
Ag1	S1	2.9160(5)	C9	C8	1.389(3)
Ag1	S2^{1}	2.5630(5)	C6	C7	1.409(2)
Ag1	N1	2.3408(17)	C5	C4	1.370(3)
S3	C11	1.7646(17)	C7	C8	1.378(3)
S1	C1	1.7558(18)	C4	C3	1.389(4)
S2	C6	1.7477(18)			

^{1}2-X,-2-Y,-1-Z; ^{2}1-X,-2-Y,-1-Z

Table S70. Bond Angles for AgSPy (2412929).

Atom	Atom	Atom	Angle/°
Ag2	Ag3	Ag1[1]	88.940(6)
Ag2	Ag3	Ag1	60.622(5)
Ag1	Ag3	Ag1[1]	88.301(6)
S3	Ag3	Ag2	78.854(11)
S3	Ag3	Ag1[1]	139.156(12)
S3	Ag3	Ag1	51.717(11)
S3	Ag3	S1[1]	110.065(14)
S1[1]	Ag3	Ag2	139.459(12)
S1[1]	Ag3	Ag1	93.109(11)
S1[1]	Ag3	Ag1[1]	57.875(10)
N2	Ag3	Ag2	93.06(4)
N2	Ag3	Ag1	151.01(4)
N2	Ag3	Ag1[1]	78.61(4)
N2	Ag3	S3	140.20(4)
N2	Ag3	S1[1]	101.32(4)
Ag3	Ag2	Ag2[2]	147.832(7)
Ag3	Ag2	Ag1	61.772(6)
Ag1	Ag2	Ag2[2]	111.590(8)
S1	Ag2	Ag3	103.548(10)
S1[2]	Ag2	Ag3	143.381(11)
S1	Ag2	Ag2[2]	53.282(10)
S1[2]	Ag2	Ag2[2]	50.170(10)
S1	Ag2	Ag1	61.443(10)
S1[2]	Ag2	Ag1	154.745(11)
S1	Ag2	S1[2]	103.452(12)
S2	Ag2	Ag3	64.942(11)
S2	Ag2	Ag2[2]	88.212(12)
S2	Ag2	Ag1	102.954(11)
S2	Ag2	S1[2]	94.445(14)
S2	Ag2	S1	83.085(14)
N3	Ag2	Ag3	88.03(4)
N3	Ag2	Ag2[2]	123.67(4)
N3	Ag2	Ag1	84.43(4)
N3	Ag2	S1	130.50(4)
N3	Ag2	S1[2]	92.89(4)
N3	Ag2	S2	142.47(4)
N1	Ag1	Ag3	149.99(4)
N1	Ag1	Ag2	92.96(4)
N1	Ag1	S3	132.86(4)
N1	Ag1	S1	59.21(4)
N1	Ag1	S2[1]	105.41(4)
Ag1	S3	Ag3	76.389(14)
C11	S3	Ag3	110.13(6)
C11	S3	Ag1	106.63(6)
Ag3[1]	S1	Ag2	116.471(16)
Ag3[1]	S1	Ag2[2]	147.660(18)
Ag3[1]	S1	Ag1	72.648(11)
Ag2	S1	Ag2[2]	76.547(12)
Ag2[2]	S1	Ag1	136.319(16)
Ag2	S1	Ag1	65.295(11)
C1	S1	Ag3[1]	96.43(6)
C1	S1	Ag2	115.75(6)
C1	S1	Ag2[2]	104.10(6)
C1	S1	Ag1	76.01(6)
Ag1[1]	S2	Ag2	115.672(17)
C6	S2	Ag2	112.79(6)
C6	S2	Ag1[1]	96.96(6)
C15	N3	Ag2	121.00(12)
C11	N3	Ag2	120.45(11)
C11	N3	C15	118.40(16)
C10	N2	Ag3	132.71(13)
C10	N2	C6	117.94(16)
C6	N2	Ag3	108.93(11)
C1	N1	Ag1	106.87(12)
C1	N1	C5	118.48(18)
C5	N1	Ag1	131.53(14)
C13	C12	C11	119.64(16)
N3	C15	C14	123.76(17)
C15	C14	C13	118.05(16)
N3	C11	S3	120.78(13)
N3	C11	C12	121.08(15)
C12	C11	S3	118.10(13)

Ag3	Ag1	Ag3[1]	91.699(6)	N2	C10	C9	123.63(18)
Ag2	Ag1	Ag3[1]	90.691(6)	C12	C13	C14	119.04(17)
Ag2	Ag1	Ag3	57.606(5)	N1	C1	S1	116.50(14)
S3	Ag1	Ag3	51.894(11)	N1	C1	C2	121.86(17)
S3	Ag1	Ag3[1]	142.659(12)	C2	C1	S1	121.50(15)
S3	Ag1	Ag2	77.104(11)	C3	C2	C1	118.6(2)
S3	Ag1	S1	130.363(14)	C10	C9	C8	118.46(18)
S3	Ag1	S2[1]	110.777(15)	N2	C6	S2	117.11(13)
S1	Ag1	Ag3	94.709(10)	N2	C6	C7	121.73(17)
S1	Ag1	Ag3[1]	49.477(9)	C7	C6	S2	121.14(14)
S1	Ag1	Ag2	53.262(9)	N1	C5	C4	123.3(2)
S2[1]	Ag1	Ag3[1]	59.510(11)	C8	C7	C6	119.22(18)
S2[1]	Ag1	Ag3	96.400(11)	C7	C8	C9	118.99(17)
S2[1]	Ag1	Ag2	141.336(12)	C5	C4	C3	118.21(19)
S2[1]	Ag1	S1	108.237(14)	C2	C3	C4	119.5(2)
N1	Ag1	Ag3[1]	82.20(4)				

[1]2-X,-2-Y,-1-Z; [2]1-X,-2-Y,-1-Z

Table S71. Torsion Angles for AgSPy (2412929).

A	B	C	D	Angle/°	A	B	C	D	Angle/°
Ag3	S3	C11	N3	-36.65(15)	S1	C1	C2	C3	-173.43(14)
Ag3	S3	C11	C12	145.44(13)	S2	C6	C7	C8	178.03(14)
Ag3[1]	S1	C1	N1	-80.50(13)	N3	C15	C14	C13	-0.7(3)
Ag3[1]	S1	C1	C2	95.36(14)	N2	C10	C9	C8	0.6(3)
Ag3	N2	C10	C9	-173.41(13)	N2	C6	C7	C8	-0.5(3)
Ag3	N2	C6	S2	-3.40(15)	N1	C1	C2	C3	2.2(3)
Ag3	N2	C6	C7	175.19(13)	N1	C5	C4	C3	1.8(3)
Ag2	S1	C1	N1	43.03(15)	C15	N3	C11	S3	-176.42(13)
Ag2[2]	S1	C1	N1	124.68(12)	C15	N3	C11	C12	1.4(3)
Ag2	S1	C1	C2	-141.11(13)	C15	C14	C13	C12	0.2(3)
Ag2[2]	S1	C1	C2	-59.46(15)	C11	N3	C15	C14	-0.1(3)
Ag2	S2	C6	N2	-49.26(14)	C11	C12	C13	C14	1.0(3)
Ag2	S2	C6	C7	132.13(13)	C10	N2	C6	S2	-176.95(13)
Ag2	N3	C15	C14	-175.74(14)	C10	N2	C6	C7	1.6(2)
Ag2	N3	C11	S3	-0.76(19)	C10	C9	C8	C7	0.6(3)
Ag2	N3	C11	C12	177.09(13)	C13	C12	C11	S3	176.00(14)
Ag1	S3	C11	N3	44.60(15)	C13	C12	C11	N3	-1.9(3)
Ag1	S3	C11	C12	-133.31(13)	C1	N1	C5	C4	-1.5(3)
Ag1	S1	C1	N1	-10.22(12)	C1	C2	C3	C4	-1.8(3)
Ag1	S1	C1	C2	165.64(15)	C6	N2	C10	C9	-1.7(3)
Ag1[1]	S2	C6	N2	72.38(13)	C6	C7	C8	C9	-0.6(3)
Ag1[1]	S2	C6	C7	-106.22(14)	C5	N1	C1	S1	175.26(14)
Ag1	N1	C1	S1	12.95(15)	C5	N1	C1	C2	-0.6(3)
Ag1	N1	C1	C2	-162.89(14)	C5	C4	C3	C2	-0.1(3)
Ag1	N1	C5	C4	155.65(16)					

[1]2-X,-2-Y,-1-Z; [2]1-X,-2-Y,-1-Z

Table S72. Hydrogen Atom Coordinates (Å×10^4) and Isotropic Displacement Parameters (Å^2×10^3) for AgSPy (2412929).

**Atom**	***x***	***y***	***z***	**U(eq)**
H12	8178.91	-4313.23	-4238.03	15
H15	3528.13	-7070.67	-4280.93	16
H14	3014.17	-4910.06	-4120.7	17
H10	10463	-9283.22	-2425.64	17
H13	5386.94	-3491.52	-4095.9	17
H2	5404.86	-10571.2	-6953.04	20
H9	9690.7	-10763.2	-1655.98	20
H5	7998.39	-6597.68	-6857.12	24
H7	7079.6	-12720.58	-3018.52	17
H8	7947.98	-12515.07	-1952.32	19
H4	6848.21	-7269.37	-7818.86	30
H3	5518.59	-9291.59	-7874.1	28

14.11 [AgSPh-pNH$_2$]·1H$_2$O (CCDC-2412913)

Table S73. Fractional Atomic Coordinates (×10^4) and Equivalent Isotropic Displacement Parameters (Å^2×10^3) for [AgSPh-pNH$_2$]·1H$_2$O (2412913). U_{eq} is defined as 1/3 of the trace of the orthogonalised U_{IJ} tensor.

**Atom**	***x***	***y***	***z***	**U(eq)**
Ag2	1403.4(13)	2612.1(2)	2484.7(10)	19.5(2)
Ag1	6449.1(13)	2573.2(2)	7333.3(10)	21.1(2)
S1	4629(4)	2883.1(7)	4634(3)	15.3(5)
S2	-463(4)	2879.9(7)	-294(3)	16.1(5)
O1	8010(15)	4927(2)	7574(11)	30.5(18)
N2	2688(16)	4600(3)	-1015(12)	23(2)
C1	5583(16)	3388(3)	4386(12)	13.3(19)
C7	533(16)	3387(3)	-526(12)	14.0(19)
N1	7507(17)	4619(3)	4069(12)	24(2)
C12	2599(18)	3480(3)	-1412(13)	21(2)
C8	-810(18)	3695(3)	184(14)	20(2)
C6	4051(18)	3668(3)	3567(14)	20(2)
C2	7739(18)	3518(3)	5082(13)	18(2)
C11	3326(18)	3880(3)	-1574(13)	19(2)
C10	1989(17)	4188(3)	-848(13)	18(2)
C5	4684(19)	4068(3)	3438(14)	23(2)
C3	8400(17)	3925(3)	4967(14)	20(2)
C4	6863(18)	4202(3)	4128(13)	18(2)
C9	-81(19)	4090(3)	43(13)	20(2)

Table S74. Anisotropic Displacement Parameters (Å^2×10^3) for [AgSPh-pNH$_2$]·1H$_2$O (2412913). The Anisotropic displacement factor exponent takes the form: $-2\pi^2[h^2a^{*2}U_{11}+2hka^*b^*U_{12}+\ldots]$.

**Atom**	**U_{11}**	**U_{22}**	**U_{33}**	**U_{23}**	**U_{13}**	**U_{12}**
Ag2	11.9(4)	26.4(4)	20.1(4)	3.6(3)	-3.0(3)	-1.0(3)
Ag1	12.6(4)	30.6(5)	20.0(4)	8.9(3)	-3.1(3)	-0.7(3)
S1	14.5(11)	16.5(11)	14.7(12)	-0.8(9)	-4.2(9)	-1.7(9)
S2	16.2(12)	17.3(12)	14.6(12)	1.5(9)	-4.1(10)	-1.3(9)
O1	42(5)	26(4)	23(4)	-3(3)	-3(4)	6(4)
N2	27(5)	23(5)	21(5)	-3(4)	0(4)	-8(4)
C1	13(5)	16(5)	12(5)	-1(4)	3(4)	-5(4)
C7	11(5)	18(5)	13(5)	0(4)	-8(4)	-2(4)
N1	34(5)	19(4)	18(5)	2(3)	-3(4)	-5(4)
C12	23(5)	29(6)	11(5)	3(4)	1(4)	1(4)
C8	19(5)	22(5)	19(5)	1(4)	1(4)	1(4)
C6	18(5)	24(5)	17(5)	3(4)	-5(4)	-2(4)
C2	21(5)	20(5)	14(5)	-3(4)	2(4)	1(4)
C11	20(5)	22(5)	15(5)	4(4)	-1(4)	-4(4)
C10	18(5)	19(5)	17(5)	4(4)	-3(4)	-7(4)
C5	28(6)	22(5)	17(5)	0(4)	-4(4)	2(4)
C3	12(5)	30(6)	19(5)	3(4)	-1(4)	-1(4)
C4	22(5)	17(5)	16(5)	4(4)	6(4)	-2(4)
C9	23(5)	21(5)	15(5)	-2(4)	-6(4)	5(4)

Table S75. Bond Lengths for [AgSPh-pNH$_2$]·1H$_2$O (2412913).

**Atom**	**Atom**	**Length/Å**	**Atom**	**Atom**	**Length/Å**
Ag2	Ag1^1	2.9021(11)	C1	C2	1.373(13)
Ag2	Ag1^2	2.8462(11)	C7	C12	1.384(14)
Ag2	S1	2.551(2)	C7	C8	1.386(14)
Ag2	S2^3	2.571(3)	N1	C4	1.434(12)
Ag2	S2	2.464(2)	C12	C11	1.396(14)
Ag1	S1	2.460(2)	C8	C9	1.382(14)
Ag1	S1^3	2.526(3)	C6	C5	1.382(14)
Ag1	S2^4	2.648(2)	C2	C3	1.405(14)
S1	C1	1.775(9)	C11	C10	1.389(15)
S2	C7	1.785(9)	C10	C9	1.391(15)
N2	C10	1.430(13)	C5	C4	1.388(14)
C1	C6	1.395(13)	C3	C4	1.399(14)

^{1}+X,1/2-Y,-1/2+Z; ^{2}-1+X,1/2-Y,-1/2+Z; ^{3}+X,1/2-Y,1/2+Z; ^{4}1+X,+Y,1+Z

Table S76. Bond Angles for $[AgSPh\text{-}pNH_2]\cdot 1H_2O$ (2412913).

Atom	Atom	Atom	Angle/°	Atom	Atom	Atom	Angle/°
Ag1[1]	Ag2	Ag1[2]	154.83(4)	Ag2	S2	Ag2[2]	98.07(8)
S1	Ag2	Ag1[2]	54.73(6)	Ag2	S2	Ag1[6]	132.77(10)
S1	Ag2	Ag1[1]	141.04(7)	Ag2[2]	S2	Ag1[6]	66.08(6)
S1	Ag2	S2[3]	96.56(8)	C7	S2	Ag2	107.2(3)
S2	Ag2	Ag1[1]	69.61(6)	C7	S2	Ag2[2]	113.9(3)
S2[3]	Ag2	Ag1[1]	58.27(5)	C7	S2	Ag1[6]	120.0(3)
S2	Ag2	Ag1[2]	115.96(7)	C6	C1	S1	119.4(7)
S2[3]	Ag2	Ag1[2]	107.70(6)	C2	C1	S1	121.5(7)
S2	Ag2	S1	132.83(8)	C2	C1	C6	118.9(9)
S2	Ag2	S2[3]	126.91(10)	C12	C7	S2	121.8(8)
Ag2[4]	Ag1	Ag2[3]	154.83(4)	C12	C7	C8	119.1(9)
S1[3]	Ag1	Ag2[3]	55.54(5)	C8	C7	S2	119.1(8)
S1	Ag1	Ag2[3]	74.70(6)	C7	C12	C11	120.4(10)
S1	Ag1	Ag2[4]	120.49(7)	C9	C8	C7	120.7(10)
S1[3]	Ag1	Ag2[4]	104.75(6)	C5	C6	C1	121.0(9)
S1	Ag1	S1[3]	129.87(10)	C1	C2	C3	120.7(9)
S1	Ag1	S2[5]	129.49(8)	C10	C11	C12	120.5(10)
S1[3]	Ag1	S2[5]	92.66(8)	C11	C10	N2	121.4(9)
S2[5]	Ag1	Ag2[4]	55.65(6)	C11	C10	C9	118.6(9)
S2[5]	Ag1	Ag2[3]	132.65(6)	C9	C10	N2	120.0(9)
Ag1[2]	S1	Ag2	69.74(6)	C6	C5	C4	120.6(9)
Ag1	S1	Ag2	129.71(10)	C4	C3	C2	120.1(9)
Ag1	S1	Ag1[2]	97.91(8)	C5	C4	N1	121.3(9)
C1	S1	Ag2	118.7(3)	C5	C4	C3	118.8(9)
C1	S1	Ag1	111.2(3)	C3	C4	N1	119.9(9)
C1	S1	Ag1[2]	111.7(3)	C8	C9	C10	120.8(10)

[1]-1+X,1/2-Y,-1/2+Z; [2]+X,1/2-Y,-1/2+Z; [3]+X,1/2-Y,1/2+Z; [4]1+X,1/2-Y,1/2+Z; [5]1+X,+Y,1+Z; [6]-1+X,+Y,-1+Z

Table S77. Torsion Angles for $[AgSPh\text{-}pNH_2]\cdot 1H_2O$ (2412913).

A	B	C	D	Angle/°	A	B	C	D	Angle/°
Ag2	S1	C1	C6	24.5(9)	N2	C10	C9	C8	178.6(9)
Ag2	S1	C1	C2	-159.6(7)	C1	C6	C5	C4	-0.2(16)
Ag2	S2	C7	C12	88.9(8)	C1	C2	C3	C4	-0.4(15)
$Ag2^{1}$	S2	C7	C12	-18.4(9)	C7	C12	C11	C10	0.0(14)
$Ag2^{1}$	S2	C7	C8	160.9(7)	C7	C8	C9	C10	1.5(15)
Ag2	S2	C7	C8	-91.8(7)	C12	C7	C8	C9	-1.3(14)
Ag1	S1	C1	C6	-149.2(7)	C12	C11	C10	N2	-179.3(9)
$Ag1^{1}$	S1	C1	C6	102.5(8)	C12	C11	C10	C9	0.1(14)
$Ag1^{1}$	S1	C1	C2	-81.6(8)	C8	C7	C12	C11	0.5(14)
Ag1	S1	C1	C2	26.7(9)	C6	C1	C2	C3	-0.2(14)
$Ag1^{2}$	S2	C7	C12	-93.6(8)	C6	C5	C4	N1	-177.5(10)
$Ag1^{2}$	S2	C7	C8	85.8(8)	C6	C5	C4	C3	-0.4(15)
S1	C1	C6	C5	176.5(8)	C2	C1	C6	C5	0.5(15)
S1	C1	C2	C3	-176.1(8)	C2	C3	C4	N1	177.8(9)
S2	C7	C12	C11	179.8(7)	C2	C3	C4	C5	0.7(15)
S2	C7	C8	C9	179.4(7)	C11	C10	C9	C8	-0.8(14)

^{1}+X,1/2-Y,-1/2+Z; ^{2}-1+X,+Y,-1+Z

Table S78. Hydrogen Atom Coordinates (Å×10^4) and Isotropic Displacement Parameters (Å^2×10^3) for [AgSPh-pNH_2]·1H_2O (2412913).

**Atom**	***x***	***y***	***z***	**U(eq)**
H1C	7654.73	4828.69	6549.2	46
H1D	9402.61	4838.49	7815.81	46
H2A	4102.96	4620.2	-1450.52	28
H2B	2708.28	4709.57	39.92	28
H1A	8903.25	4684.23	3692.97	28
H1B	6437.45	4751.23	3450.48	28
H12	3506.93	3275.84	-1901.57	25
H8	-2217.13	3635.28	760.18	24
H6	2581.22	3582.97	3100.19	24
H2	8772.68	3334.87	5634.12	22
H11	4716.26	3939.83	-2172.01	23
H5	3639.52	4249.28	2883.55	27
H3	9860.1	4009.42	5449.14	24
H9	-983.37	4293.41	550.48	24

14.12 [AgSPh-mNO$_2$]$_4$·1DAP (CCDC-2412943)

Table S79. Fractional Atomic Coordinates ($\times10^4$) and Equivalent Isotropic Displacement Parameters ($Å^2\times10^3$) for [AgSPh-mNO$_2$]$_4$·1DAP (2412943). U_{eq} is defined as 1/3 of the trace of the orthogonalised U_{IJ} tensor.

Atom	*x*	*y*	*z*	U(eq)
Ag1	7081.5(5)	6385.2(2)	5084.0(2)	21.03(9)
Ag2	8894.1(5)	7959.9(3)	6197.1(2)	22.91(9)
Ag3	6870.8(5)	8371.2(3)	4870.0(2)	23.43(9)
Ag4	3672.3(5)	8102.5(3)	5923.9(2)	25.43(10)
S4	9330.8(15)	6596.5(9)	5648.2(4)	17.8(2)
S2	6489.4(15)	8717.4(9)	5747.9(5)	19.3(3)
S1	6084.6(16)	6097.1(9)	4251.8(5)	20.0(3)
S3	4409.1(17)	6570.2(10)	5587.1(5)	23.3(3)
O1	9231(5)	3252(3)	4888.4(16)	36.3(10)
O2	9040(6)	2216(3)	4338.9(17)	37.0(11)
O7	7407(6)	5086(4)	7305.5(16)	40.0(11)
N5	8709(6)	8037(3)	7035.6(16)	25.5(10)
O5	1290(5)	6477(3)	7261.9(15)	30.9(9)
N6	3551(6)	8234(4)	6800.1(17)	29.7(11)
N1	8755(6)	2973(3)	4489.9(17)	25.9(10)
O6	1424(6)	5135(3)	7558.9(16)	36.1(11)
N2	6212(7)	11693(3)	6712.6(19)	33.0(12)
O8	8571(6)	3790(3)	7358.2(17)	42.0(12)
O4	5522(6)	11256(3)	7026.9(17)	43.6(12)
C14	3022(6)	6027(3)	6450.6(18)	18.8(10)
C26	5988(7)	7386(4)	7010(2)	22.8(11)
C7	6916(6)	9877(3)	5800.6(19)	18.3(10)
C19	9416(6)	5534(3)	5945.4(19)	16.9(10)
N3	1777(6)	5698(3)	7244.0(17)	26.3(10)
N4	8241(6)	4484(4)	7134.7(18)	31.3(12)
C12	7821(6)	10341(4)	5449.5(19)	20.2(10)
C2	7485(6)	4429(3)	4348.0(19)	18.9(10)
C8	6388(6)	10339(3)	6214(2)	20.6(10)
C1	6563(6)	4997(3)	4056.5(19)	18.5(10)
C13	4046(6)	5806(4)	6070.2(19)	19.7(11)
C20	8753(7)	5422(3)	6411.3(19)	19.6(10)
C24	10145(6)	4805(4)	5716(2)	21.3(11)
C25	7575(7)	7408(4)	7268(2)	25.9(12)

O3	6496(8)	12503(3)	6753(2)	54.1(16)
C15	2807(6)	5429(4)	6836.3(19)	21.0(11)
C11	8163(7)	11248(4)	5507(2)	25.1(12)
C23	10260(7)	3985(4)	5951(2)	25.4(12)
C6	5985(7)	4685(4)	3605.5(19)	23.7(12)
C3	7780(6)	3569(3)	4178.1(19)	21.3(11)
C21	8891(6)	4590(4)	6636(2)	21.9(11)
C10	7629(7)	11714(4)	5917(2)	27.5(12)
C27	5057(7)	8252(4)	7077(2)	25.8(12)
C16	3549(8)	4604(4)	6849(2)	30.2(13)
C5	6314(7)	3818(4)	3447(2)	25.8(12)
C9	6762(7)	11240(4)	6266(2)	25.2(12)
C18	4809(7)	4969(4)	6079(2)	27.3(13)
C22	9644(7)	3866(4)	6420(2)	26.7(12)
C4	7236(7)	3248(4)	3731(2)	25.0(12)
C17	4547(8)	4378(4)	6466(2)	34.2(14)

Table S80. Anisotropic Displacement Parameters (Å^2×10^3) for [AgSPh-mNO$_2$]$_4$·1DAP (2412943). The Anisotropic displacement factor exponent takes the form: -2π^2[h^{2}a*^{2}U$_{11}$+2hka*b*U$_{12}$+…].

**Atom**	**U11**	**U22**	**U33**	**U23**	**U13**	**U12**
Ag1	27.2(2)	16.51(17)	19.35(18)	0.00(15)	-5.22(16)	1.15(15)
Ag2	26.0(2)	22.55(19)	20.17(19)	0.49(16)	3.23(16)	1.27(17)
Ag3	31.4(2)	17.16(17)	21.71(19)	2.31(15)	-2.23(17)	-0.05(16)
Ag4	23.3(2)	25.4(2)	27.5(2)	5.03(18)	5.30(17)	2.37(17)
S4	19.5(6)	17.4(6)	16.6(6)	0.4(5)	-0.9(5)	0.9(5)
S2	19.0(6)	18.1(6)	20.8(6)	1.7(5)	0.9(5)	-2.6(5)
S1	19.4(6)	21.7(6)	18.7(6)	-3.1(5)	-2.5(5)	2.9(5)
S3	23.2(6)	28.5(7)	18.3(6)	-1.5(5)	5.3(5)	-2.0(6)
O1	50(3)	27(2)	32(2)	-3.4(19)	-13(2)	3.9(19)
O2	52(3)	18.3(19)	40(3)	-3.4(18)	-4(2)	7(2)
O7	42(3)	57(3)	21(2)	-2(2)	14(2)	0(2)
N5	22(2)	36(3)	18(2)	-7(2)	-1.4(18)	-2(2)
O5	35(2)	34(2)	23(2)	4.8(18)	4.2(18)	0(2)
N6	20(2)	43(3)	26(3)	-6(2)	4.1(19)	1(2)
N1	31(2)	18(2)	28(2)	4(2)	0(2)	-3(2)
O6	42(3)	40(2)	26(2)	10.3(19)	1.3(19)	-13(2)
N2	39(3)	31(3)	29(3)	-7(2)	-9(2)	15(2)
O8	36(3)	57(3)	33(2)	26(2)	1(2)	-5(2)
O4	49(3)	51(3)	31(2)	-9(2)	10(2)	6(2)
C14	18(2)	19(2)	19(2)	-1.7(19)	-1(2)	0(2)
C26	32(3)	21(2)	15(3)	1(2)	4(2)	-5(2)
C7	15(2)	21(2)	20(2)	-0.3(19)	-3(2)	1(2)
C19	14(2)	18(2)	19(2)	-3(2)	-1(2)	2.7(18)
N3	24(2)	37(3)	18(2)	6(2)	0.1(19)	-9(2)
N4	24(3)	47(3)	23(2)	13(2)	-3(2)	-15(2)
C12	18(3)	24(3)	18(2)	2(2)	-2(2)	-3(2)
C2	20(3)	21(3)	16(2)	-3(2)	5.4(19)	-3(2)
C8	21(3)	21(2)	20(2)	0(2)	0(2)	2(2)
C1	15(2)	22(2)	18(2)	0(2)	5(2)	-2.2(18)
C13	15(3)	20(2)	25(3)	-4(2)	-2(2)	-3(2)
C20	17(2)	24(2)	19(2)	0(2)	-1(2)	-2(2)
C24	23(3)	23(3)	18(3)	-2(2)	1(2)	-1(2)
C25	36(3)	27(3)	14(3)	-1(2)	3(2)	2(2)
O3	92(5)	27(2)	43(3)	-13(2)	-6(3)	15(3)

C15	20(3)	25(3)	19(2)	1(2)	-6(2)	-5(2)
C11	25(3)	20(2)	30(3)	9(2)	-2(2)	-4(2)
C23	32(3)	18(3)	26(3)	-3(2)	0(2)	4(2)
C6	27(3)	28(3)	16(2)	2(2)	0(2)	0(2)
C3	22(3)	21(2)	21(2)	1(2)	0(2)	-3(2)
C21	13(2)	31(3)	22(3)	5(2)	-2(2)	-5(2)
C10	32(3)	19(3)	31(3)	1(2)	-10(2)	-1(2)
C27	29(3)	27(3)	21(3)	-2(2)	1(2)	0(2)
C16	32(3)	23(3)	35(3)	7(2)	-9(3)	-4(2)
C5	33(3)	29(3)	15(2)	-4(2)	-2(2)	-3(3)
C9	28(3)	24(3)	23(3)	-4(2)	-4(2)	8(2)
C18	23(3)	24(3)	35(3)	-7(2)	-2(2)	-1(2)
C22	26(3)	24(3)	31(3)	6(2)	-3(2)	-2(2)
C4	31(3)	22(3)	22(3)	-2(2)	7(2)	-2(2)
C17	31(3)	20(3)	51(4)	1(3)	-7(3)	4(2)

Table S81. Bond Lengths for $[AgSPh\text{-}mNO_2]_4 \cdot 1DAP$ (2412943).

Atom	Atom	Length/Å	Atom	Atom	Length/Å
Ag1	Ag3	3.0245(6)	N2	C9	1.465(7)
Ag1	Ag4[1]	3.1434(6)	O8	N4	1.233(7)
Ag1	S4	2.4533(13)	C14	C13	1.385(7)
Ag1	S1	2.4511(14)	C14	C15	1.389(7)
Ag1	S3	2.6420(14)	C26	C25	1.505(8)
Ag2	S4	2.5496(14)	C26	C27	1.521(8)
Ag2	S2	2.6162(14)	C7	C12	1.403(7)
Ag2	S1[1]	2.6173(14)	C7	C8	1.392(7)
Ag2	N5	2.289(4)	C19	C20	1.394(7)
Ag3	S4[2]	2.5561(13)	C19	C24	1.394(7)
Ag3	S2	2.4636(14)	N3	C15	1.463(7)
Ag3	S3[1]	2.4683(15)	N4	C21	1.470(7)
Ag4	S2	2.5811(13)	C12	C11	1.392(7)
Ag4	S1[2]	2.5243(15)	C2	C1	1.395(7)
Ag4	S3	2.5389(15)	C2	C3	1.385(7)
Ag4	N6	2.393(5)	C8	C9	1.387(7)
S4	C19	1.781(5)	C1	C6	1.399(7)
S2	C7	1.772(5)	C13	C18	1.403(8)
S1	C1	1.770(5)	C20	C21	1.389(7)
S3	C13	1.766(5)	C24	C23	1.384(8)
O1	N1	1.228(6)	C15	C16	1.380(8)
O2	N1	1.224(6)	C11	C10	1.387(8)
O7	N4	1.229(7)	C23	C22	1.388(8)
N5	C25	1.478(7)	C6	C5	1.391(8)
O5	N3	1.232(7)	C3	C4	1.384(7)
N6	C27	1.471(7)	C21	C22	1.383(8)
N1	C3	1.477(7)	C10	C9	1.390(8)
O6	N3	1.235(6)	C16	C17	1.377(9)
N2	O4	1.222(7)	C5	C4	1.386(8)
N2	O3	1.236(7)	C18	C17	1.391(9)

[1]1/2+X,3/2-Y,1-Z; [2]-1/2+X,3/2-Y,1-Z

Table S82. Bond Angles for [AgSPh-mNO_2]$_4$·1DAP (2412943).

Atom	Atom	Atom	Angle/°	Atom	Atom	Atom	Angle/°
Ag3	Ag1	Ag4[1]	67.597(14)	C27	N6	Ag4	118.3(3)
S4	Ag1	Ag3	92.26(3)	O1	N1	C3	118.8(5)
S4	Ag1	Ag4[1]	100.79(3)	O2	N1	O1	123.1(5)
S4	Ag1	S3	108.42(4)	O2	N1	C3	118.1(5)
S1	Ag1	Ag3	88.52(3)	O4	N2	O3	123.5(6)
S1	Ag1	Ag4[1]	51.86(3)	O4	N2	C9	118.9(5)
S1	Ag1	S4	149.64(5)	O3	N2	C9	117.6(6)
S1	Ag1	S3	101.93(5)	C13	C14	C15	119.5(5)
S3	Ag1	Ag3	87.00(3)	C25	C26	C27	112.4(5)
S3	Ag1	Ag4[1]	141.90(3)	C12	C7	S2	122.4(4)
S4	Ag2	S2	100.49(4)	C8	C7	S2	119.0(4)
S4	Ag2	S1[1]	93.13(4)	C8	C7	C12	118.5(5)
S2	Ag2	S1[1]	95.28(4)	C20	C19	S4	120.2(4)
N5	Ag2	S4	129.28(13)	C20	C19	C24	119.3(5)
N5	Ag2	S2	113.05(13)	C24	C19	S4	120.6(4)
N5	Ag2	S1[1]	119.04(12)	O5	N3	O6	122.3(5)
S4[2]	Ag3	Ag1	99.98(3)	O5	N3	C15	119.0(4)
S2	Ag3	Ag1	91.52(3)	O6	N3	C15	118.7(5)
S2	Ag3	S4[2]	114.96(4)	O7	N4	O8	123.8(5)
S2	Ag3	S3[1]	126.36(5)	O7	N4	C21	118.8(5)
S3[1]	Ag3	Ag1	94.65(4)	O8	N4	C21	117.5(5)
S3[1]	Ag3	S4[2]	116.21(4)	C11	C12	C7	121.0(5)
S2	Ag4	Ag1[2]	98.10(3)	C3	C2	C1	118.2(5)
S1[2]	Ag4	Ag1[2]	49.79(3)	C9	C8	C7	119.3(5)
S1[2]	Ag4	S2	125.81(4)	C2	C1	S1	121.2(4)
S1[2]	Ag4	S3	124.55(5)	C2	C1	C6	119.2(5)
S3	Ag4	Ag1[2]	90.45(3)	C6	C1	S1	119.6(4)
S3	Ag4	S2	91.71(5)	C14	C13	S3	120.6(4)
N6	Ag4	Ag1[2]	146.14(12)	C14	C13	C18	118.8(5)
N6	Ag4	S2	101.23(12)	C18	C13	S3	120.6(4)
N6	Ag4	S1[2]	96.50(12)	C21	C20	C19	118.3(5)
N6	Ag4	S3	116.31(13)	C23	C24	C19	121.0(5)
Ag1	S4	Ag2	110.98(5)	N5	C25	C26	112.7(5)
Ag1	S4	Ag3[1]	107.43(5)	C14	C15	N3	118.2(5)
Ag2	S4	Ag3[1]	115.32(5)	C16	C15	C14	122.2(5)

C19	S4	Ag1	101.56(17)
C19	S4	Ag2	116.75(17)
C19	S4	Ag3[1]	103.50(17)
Ag3	S2	Ag2	105.18(5)
Ag3	S2	Ag4	102.96(5)
Ag4	S2	Ag2	117.82(5)
C7	S2	Ag2	103.16(17)
C7	S2	Ag3	104.88(18)
C7	S2	Ag4	121.14(18)
Ag1	S1	Ag2[2]	125.20(5)
Ag1	S1	Ag4[1]	78.35(4)
Ag4[1]	S1	Ag2[2]	105.19(5)
C1	S1	Ag1	111.22(18)
C1	S1	Ag2[2]	121.16(18)
C1	S1	Ag4[1]	100.75(17)
Ag3[2]	S3	Ag1	118.40(5)
Ag3[2]	S3	Ag4	86.54(5)
Ag4	S3	Ag1	119.19(5)
C13	S3	Ag1	117.67(19)
C13	S3	Ag3[2]	104.40(18)
C13	S3	Ag4	105.70(18)
C25	N5	Ag2	115.9(3)
C16	C15	N3	119.6(5)
C10	C11	C12	120.7(5)
C24	C23	C22	120.7(5)
C5	C6	C1	120.8(5)
C2	C3	N1	117.7(5)
C4	C3	N1	118.5(5)
C4	C3	C2	123.7(5)
C20	C21	N4	118.1(5)
C22	C21	N4	118.6(5)
C22	C21	C20	123.3(5)
C11	C10	C9	117.6(5)
N6	C27	C26	111.4(5)
C17	C16	C15	118.3(6)
C4	C5	C6	120.6(5)
C8	C9	N2	117.3(5)
C8	C9	C10	122.8(5)
C10	C9	N2	119.8(5)
C17	C18	C13	120.4(6)
C21	C22	C23	117.5(5)
C3	C4	C5	117.4(5)
C16	C17	C18	120.8(6)

[1]1/2+X,3/2-Y,1-Z; [2]-1/2+X,3/2-Y,1-Z

Table S83. Torsion Angles for $[AgSPh\text{-}mNO_2]_4 \cdot 1DAP$ (2412943).

A	B	C	D	Angle/°	A	B	C	D	Angle/°
Ag1	S4	C19	C20	-99.9(4)	O6	N3	C15	C16	-9.6(8)
Ag1	S4	C19	C24	80.2(4)	O8	N4	C21	C20	-169.7(5)
Ag1	S1	C1	C2	-3.9(5)	O8	N4	C21	C22	8.8(8)
Ag1	S1	C1	C6	175.1(4)	O4	N2	C9	C8	3.3(8)
Ag1	S3	C13	C14	157.1(4)	O4	N2	C9	C10	-176.3(6)
Ag1	S3	C13	C18	-21.9(5)	C14	C13	C18	C17	-0.5(8)
Ag2	S4	C19	C20	21.0(5)	C14	C15	C16	C17	0.7(9)
Ag2	S4	C19	C24	-159.0(4)	C7	C12	C11	C10	0.7(8)
Ag2	S2	C7	C12	89.4(4)	C7	C8	C9	N2	-178.6(5)
Ag2	S2	C7	C8	-88.1(4)	C7	C8	C9	C10	1.0(8)
Ag2[1]	S1	C1	C2	-167.1(3)	C19	C20	C21	N4	177.9(5)
Ag2[1]	S1	C1	C6	11.9(5)	C19	C20	C21	C22	-0.5(8)
Ag2	N5	C25	C26	-46.3(6)	C19	C24	C23	C22	0.3(9)
Ag3[2]	S4	C19	C20	148.8(4)	N3	C15	C16	C17	-178.2(5)
Ag3[2]	S4	C19	C24	-31.1(5)	N4	C21	C22	C23	-179.5(5)
Ag3	S2	C7	C12	-20.5(5)	C12	C7	C8	C9	0.3(8)
Ag3	S2	C7	C8	162.0(4)	C12	C11	C10	C9	0.6(8)
Ag3[1]	S3	C13	C14	-69.3(4)	C2	C1	C6	C5	0.3(8)
Ag3[1]	S3	C13	C18	111.6(4)	C2	C3	C4	C5	-1.6(8)
Ag4	S2	C7	C12	-136.1(4)	C8	C7	C12	C11	-1.1(8)
Ag4	S2	C7	C8	46.4(5)	C1	C2	C3	N1	-179.9(5)
Ag4[2]	S1	C1	C2	77.7(4)	C1	C2	C3	C4	1.3(8)
Ag4[2]	S1	C1	C6	-103.4(4)	C1	C6	C5	C4	-0.7(9)
Ag4	S3	C13	C14	21.1(5)	C13	C14	C15	N3	177.2(5)
Ag4	S3	C13	C18	-157.9(4)	C13	C14	C15	C16	-1.8(8)
Ag4	N6	C27	C26	-62.2(5)	C13	C18	C17	C16	-0.5(9)
S4	C19	C20	C21	-177.9(4)	C20	C19	C24	C23	-2.0(8)
S4	C19	C24	C23	178.0(4)	C20	C21	C22	C23	-1.2(9)
S2	C7	C12	C11	-178.7(4)	C24	C19	C20	C21	2.0(8)
S2	C7	C8	C9	177.9(4)	C24	C23	C22	C21	1.2(9)
S1	C1	C6	C5	-178.6(4)	C25	C26	C27	N6	176.9(4)
S3	C13	C18	C17	178.5(5)	O3	N2	C9	C8	-177.6(6)
O1	N1	C3	C2	1.6(7)	O3	N2	C9	C10	2.7(9)
O1	N1	C3	C4	-179.5(5)	C15	C14	C13	S3	-177.4(4)
O2	N1	C3	C2	-179.0(5)	C15	C14	C13	C18	1.7(8)
O2	N1	C3	C4	-0.1(8)	C15	C16	C17	C18	0.5(9)

O7	N4	C21	C20	10.2(8)	C11	C10	C9	N2	178.1(5)
O7	N4	C21	C22	-171.4(5)	C11	C10	C9	C8	-1.5(8)
O5	N3	C15	C14	-8.5(7)	C6	C5	C4	C3	1.3(8)
O5	N3	C15	C16	170.5(5)	C3	C2	C1	S1	178.4(4)
N1	C3	C4	C5	179.5(5)	C3	C2	C1	C6	-0.6(7)
O6	N3	C15	C14	171.5(5)	C27	C26	C25	N5	-69.1(6)

[1]-1/2+X,3/2-Y,1-Z; [2]1/2+X,3/2-Y,1-Z

Table S84. Hydrogen Atom Coordinates (Å×10^4) and Isotropic Displacement Parameters (Å^2×10^3) for $[AgSPh\text{-}mNO_2]_4\cdot 1DAP$ (2412943).

**Atom**	***x***	***y***	***z***	**U(eq)**
H5A	9693.26	7934.39	7164.52	31
H5B	8423.23	8604.78	7119.43	31
H6A	3007.39	8744.66	6872.32	36
H6B	2962.04	7766.76	6915.74	36
H14	2471.47	6583.6	6447.8	23
H26A	5351.3	6880.17	7140.82	27
H26B	6160.37	7278.97	6654.95	27
H12	8206.68	10032.15	5168.22	24
H2	7899.42	4625.79	4654.56	23
H8	5777.44	10040.93	6458.74	25
H20	8219.97	5903.2	6570.69	23
H24	10568.32	4873.42	5394.64	26
H25A	7411.45	7585.68	7614.6	31
H25B	8040.41	6798.23	7265.53	31
H11	8767.4	11551.82	5263.17	30
H23	10766.79	3497.44	5788.84	30
H6	5361.59	5070.06	3404.63	28
H10	7848.52	12334.55	5957.01	33
H27A	4826.81	8342.76	7430.15	31
H27B	5712.34	8763.01	6962.17	31
H16	3376.13	4201.37	7114.05	36
H5	5901.23	3614.44	3141.64	31
H18	5507.59	4805.63	5819.33	33
H22	9736.32	3308.08	6585.82	32
H4	7485.91	2658.32	3623.4	30
H17	5062.66	3810.88	6466.57	41

14.13 AgSePh-Me2(2,6) (CCDC-2412918)

Table S85. Fractional Atomic Coordinates (×10^4) and Equivalent Isotropic Displacement Parameters (Å^2×10^3) for AgSePh-Me2(2,6) (2412918). U_{eq} is defined as 1/3 of the trace of the orthogonalised U_{IJ} tensor.

Atom	*x*	*y*	*z*	U(eq)
Ag1	-16.6(9)	4478.8(3)	6080.8(3)	15.15(10)
Ag2	4822.0(9)	6877.7(3)	4702.4(3)	15.99(10)
Se1	4962.3(11)	5525.1(4)	6279.8(4)	12.07(11)
Se2	-157.9(11)	7681.4(4)	4214.5(4)	12.49(12)
C1	4635(11)	6244(4)	7538(4)	13.3(10)
C9	407(11)	8468(4)	2955(4)	12.9(10)
C15	-3064(12)	7244(4)	2075(4)	16.9(11)
C6	6090(11)	5814(4)	8362(4)	14.3(10)
C8	8096(12)	4849(5)	8328(4)	18.3(11)
C16	3533(12)	9798(5)	3857(4)	20.5(12)
C14	2242(12)	9374(4)	2942(4)	17.3(11)
C7	1108(12)	7626(4)	6776(4)	17.4(11)
C2	2776(12)	7150(4)	7625(4)	14.0(10)
C10	-888(12)	8141(4)	2092(4)	15.5(10)
C5	5690(13)	6312(5)	9262(4)	19.5(11)
C3	2457(13)	7616(4)	8538(4)	18.1(11)
C13	2915(13)	9909(4)	2037(4)	20.2(12)
C11	-154(12)	8710(4)	1199(4)	18.8(11)
C4	3877(13)	7209(5)	9358(4)	21.8(12)
C12	1768(13)	9567(5)	1169(4)	21.5(12)

Table S86. Anisotropic Displacement Parameters ($Å^2 \times 10^3$) for AgSePh-Me_2(2,6) (2412918). The Anisotropic displacement factor exponent takes the form: $-2\pi^2[h^2a^{*2}U_{11}+2hka^*b^*U_{12}+\ldots]$.

**Atom**	**U_{11}**	**U_{22}**	**U_{33}**	**U_{23}**	**U_{13}**	**U_{12}**
Ag1	14.9(2)	13.45(19)	17.1(2)	-0.80(14)	-0.44(14)	1.69(15)
Ag2	12.6(2)	16.5(2)	18.7(2)	1.11(15)	0.12(15)	1.63(15)
Se1	12.0(2)	11.0(2)	13.2(2)	-1.27(18)	0.34(18)	1.91(18)
Se2	10.9(2)	12.8(2)	13.6(2)	0.84(18)	-0.51(18)	1.63(19)
C1	13(3)	15(2)	12(2)	0.4(19)	-0.1(19)	-2(2)
C9	12(2)	10(2)	16(2)	5.3(18)	1.6(19)	3.5(19)
C15	15(3)	16(2)	19(3)	-1(2)	-3(2)	2(2)
C6	12(3)	13(2)	17(3)	0.0(19)	1.7(19)	-2.6(19)
C8	16(3)	18(3)	20(3)	1(2)	-2(2)	6(2)
C16	16(3)	17(3)	29(3)	0(2)	-4(2)	-3(2)
C14	15(3)	15(2)	22(3)	2(2)	-2(2)	7(2)
C7	23(3)	13(2)	17(3)	1(2)	0(2)	4(2)
C2	16(3)	11(2)	15(3)	0.0(19)	2.4(19)	0(2)
C10	14(3)	15(2)	18(3)	3(2)	0(2)	4(2)
C5	23(3)	23(3)	13(3)	0(2)	-2(2)	3(2)
C3	21(3)	15(2)	18(3)	-3(2)	2(2)	4(2)
C13	20(3)	13(2)	27(3)	6(2)	5(2)	3(2)
C11	20(3)	17(3)	19(3)	2(2)	0(2)	5(2)
C4	20(3)	27(3)	19(3)	-4(2)	-2(2)	6(2)
C12	21(3)	21(3)	21(3)	8(2)	2(2)	6(2)

Table S87. Bond Lengths for AgSePh-Me_2(2,6) (2412918).

Atom	Atom	Length/Å	Atom	Atom	Length/Å
Ag1	Ag1[1]	3.1491(8)	C9	C10	1.403(8)
Ag1	Ag2[2]	3.1236(6)	C15	C10	1.498(8)
Ag1	Ag2[1]	3.0317(6)	C6	C8	1.506(7)
Ag1	Se1	2.6800(7)	C6	C5	1.395(8)
Ag1	Se1[3]	2.6808(7)	C16	C14	1.504(8)
Ag1	Se2[1]	2.6915(7)	C14	C13	1.397(8)
Ag2	Se1	2.6537(6)	C7	C2	1.504(7)
Ag2	Se2[4]	2.6021(7)	C2	C3	1.389(7)
Ag2	Se2	2.6117(7)	C10	C11	1.407(7)
Se1	C1	1.954(5)	C5	C4	1.393(8)
Se2	C9	1.939(5)	C3	C4	1.384(8)
C1	C6	1.406(7)	C13	C12	1.386(9)
C1	C2	1.413(7)	C11	C12	1.380(9)
C9	C14	1.402(8)			

[1]-X,1-Y,1-Z; [2]1-X,1-Y,1-Z; [3]-1+X,+Y,+Z; [4]1+X,+Y,+Z

Table S88. Bond Angles for AgSePh-Me$_2$(2,6) (2412918).

Atom	Atom	Atom	Angle/°	Atom	Atom	Atom	Angle/°
Ag2^{1}	Ag1	Ag1^{1}	82.405(18)	Ag2	Se2	Ag1^{1}	69.718(17)
Ag2^{2}	Ag1	Ag1^{1}	84.163(17)	Ag2^{3}	Se2	Ag1^{1}	72.303(18)
Ag2^{1}	Ag1	Ag2^{2}	98.572(17)	Ag2^{3}	Se2	Ag2	127.00(3)
Se1	Ag1	Ag1^{1}	86.09(2)	C9	Se2	Ag1^{1}	108.79(15)
Se1^{3}	Ag1	Ag1^{1}	84.314(19)	C9	Se2	Ag2	106.80(15)
Se1	Ag1	Ag2^{1}	163.42(2)	C9	Se2	Ag2^{3}	119.71(16)
Se1	Ag1	Ag2^{2}	68.292(17)	C6	C1	Se1	119.9(4)
Se1^{3}	Ag1	Ag2^{2}	164.58(2)	C6	C1	C2	120.7(5)
Se1^{3}	Ag1	Ag2^{1}	69.732(17)	C2	C1	Se1	119.3(4)
Se1	Ag1	Se1^{3}	121.01(2)	C14	C9	Se2	116.4(4)
Se1^{3}	Ag1	Se2^{1}	120.84(2)	C14	C9	C10	121.3(5)
Se1	Ag1	Se2^{1}	118.04(2)	C10	C9	Se2	122.3(4)
Se2^{1}	Ag1	Ag1^{1}	103.04(2)	C1	C6	C8	123.7(5)
Se2^{1}	Ag1	Ag2^{2}	52.525(15)	C5	C6	C1	118.4(5)
Se2^{1}	Ag1	Ag2^{1}	53.905(15)	C5	C6	C8	117.8(5)
Ag1^{1}	Ag2	Ag1^{2}	98.572(16)	C9	C14	C16	123.2(5)
Se1	Ag2	Ag1^{1}	88.988(18)	C13	C14	C9	118.6(5)
Se1	Ag2	Ag1^{2}	85.264(17)	C13	C14	C16	118.2(5)
Se2^{4}	Ag2	Ag1^{2}	55.172(16)	C1	C2	C7	123.0(5)
Se2	Ag2	Ag1^{1}	56.378(16)	C3	C2	C1	118.5(5)
Se2	Ag2	Ag1^{2}	143.13(2)	C3	C2	C7	118.5(5)
Se2^{4}	Ag2	Ag1^{1}	142.03(2)	C9	C10	C15	123.6(5)
Se2^{4}	Ag2	Se1	112.51(2)	C9	C10	C11	117.9(5)
Se2	Ag2	Se1	117.37(2)	C11	C10	C15	118.4(5)
Se2^{4}	Ag2	Se2	127.00(3)	C4	C5	C6	121.6(5)
Ag1	Se1	Ag1^{4}	121.01(2)	C4	C3	C2	121.8(5)
Ag2	Se1	Ag1	99.55(2)	C12	C13	C14	121.0(6)
Ag2	Se1	Ag1^{4}	104.01(2)	C12	C11	C10	121.3(6)
C1	Se1	Ag1	105.91(16)	C3	C4	C5	119.0(5)
C1	Se1	Ag1^{4}	111.71(16)	C11	C12	C13	119.7(5)
C1	Se1	Ag2	114.37(15)				

^{1}-X,1-Y,1-Z; ^{2}1-X,1-Y,1-Z; ^{3}-1+X,+Y,+Z; ^{4}1+X,+Y,+Z

Table S89. Torsion Angles for AgSePh-Me$_2$(2,6) (2412918).

A	B	C	D	Angle/°	A	B	C	D	Angle/°
Se1	C1	C6	C8	4.2(7)	C6	C1	C2	C3	1.1(8)
Se1	C1	C6	C5	-176.6(4)	C6	C5	C4	C3	-0.4(9)
Se1	C1	C2	C7	-2.8(7)	C8	C6	C5	C4	179.9(6)
Se1	C1	C2	C3	176.7(4)	C16	C14	C13	C12	-179.2(5)
Se2	C9	C14	C16	-5.3(7)	C14	C9	C10	C15	-174.4(5)
Se2	C9	C14	C13	174.5(4)	C14	C9	C10	C11	3.8(7)
Se2	C9	C10	C15	7.1(7)	C14	C13	C12	C11	2.4(8)
Se2	C9	C10	C11	-174.7(4)	C7	C2	C3	C4	178.7(6)
C1	C6	C5	C4	0.7(9)	C2	C1	C6	C8	179.8(5)
C1	C2	C3	C4	-0.8(9)	C2	C1	C6	C5	-1.1(8)
C9	C14	C13	C12	1.0(8)	C2	C3	C4	C5	0.4(9)
C9	C10	C11	C12	-0.4(8)	C10	C9	C14	C16	176.0(5)
C15	C10	C11	C12	178.0(5)	C10	C9	C14	C13	-4.1(7)
C6	C1	C2	C7	-178.4(5)	C10	C11	C12	C13	-2.7(8)

Table S88. Hydrogen Atom Coordinates (Å×10^4) and Isotropic Displacement Parameters (Å^2×10^3) for AgSePh-Me$_2$(2,6) (2412918).

Atom	*x*	*y*	*z*	U(eq)
H15A	-2220.24	6559.79	2332.43	25
H15B	-3658.96	7154.25	1395.51	25
H15C	-4735.75	7431.53	2486.91	25
H8A	7219.55	4269.61	7959.41	27
H8B	8466.28	4575.6	9001.37	27
H8C	9905.97	5075.82	7999.35	27
H16A	2007.43	9932.26	4347.41	31
H16B	4554.32	10482.22	3690.17	31
H16C	4877.44	9256.25	4129.27	31
H7A	2436.97	7945.78	6273.29	26
H7B	-189.27	8194.45	7012.72	26
H7C	-12.43	7048.01	6487.75	26
H5	6677.14	6033.79	9822.19	23
H3	1230.33	8230.85	8601.23	22
H13	4179.16	10517.73	2016.12	24
H11	-996.79	8499.59	605.5	23
H4	3619.56	7537.02	9977.14	26
H12	2302.71	9920.66	555.73	26

14.14 AgSePh-Cl_2(2,6) (CCDC-2412930)

Table S90. Fractional Atomic Coordinates ($\times 10^4$) and Equivalent Isotropic Displacement Parameters ($Å^2 \times 10^3$) for AgSePh-Cl_2(2,6) (2412930). U_{eq} is defined as 1/3 of the trace of the orthogonalised U_{IJ} tensor.

**Atom**	***x***	***y***	***z***	**U(eq)**
Ag1	10210(3)	3809.7(8)	4716.1(4)	23.3(3)
Ag2	4974(3)	5249.0(8)	5971.9(5)	25.6(3)
Se1	5248(3)	3732.5(10)	5259.3(6)	19.1(3)
Se2	-5(3)	5726.1(10)	6359.0(6)	20.4(3)
Cl1	8767(8)	1632(2)	4871.1(14)	20.4(7)
Cl4	-3643(8)	7873(3)	6052.4(15)	21.6(7)
Cl3	4075(8)	6002(3)	7439.8(15)	26.1(8)
Cl2	1176(8)	3268(3)	6333.4(15)	24.8(8)
C1	4880(30)	2441(11)	5596(5)	20(3)
C7	290(40)	7002(10)	6744(6)	24(3)
C8	2200(40)	7066(12)	7193(6)	26.1(8)
C5	2550(30)	1313(12)	6263(6)	24(3)
C2	6290(30)	1558(10)	5405(6)	19(3)
C3	5860(30)	607(11)	5622(6)	21(3)
C12	-1090(30)	7879(11)	6586(6)	23(3)
C6	2980(30)	2270(12)	6045(6)	24(3)
C11	-710(40)	8767(11)	6883(6)	27(4)
C4	3850(30)	493(11)	6049(6)	24(3)
C10	1130(30)	8828(12)	7308(6)	27(3)
C9	2720(40)	7956(14)	7472(7)	33(4)

Table S91. Anisotropic Displacement Parameters ($Å^2 \times 10^3$) for AgSePh-Cl_2(2,6) (2412930). The Anisotropic displacement factor exponent takes the form: $-2\pi^2[h^2a^{*2}U_{11}+2hka^*b^*U_{12}+\ldots]$.

**Atom**	U_{11}	U_{22}	U_{33}	U_{23}	U_{13}	U_{12}
Ag1	29.8(6)	16.6(5)	23.5(5)	1.9(4)	-0.8(5)	0.1(5)
Ag2	27.0(6)	20.3(5)	29.5(6)	-3.8(4)	0.0(5)	-0.7(5)
Se1	25.4(8)	11.6(6)	20.3(7)	1.9(5)	-1.0(6)	0.3(6)
Se2	25.0(7)	13.7(6)	22.4(7)	-1.0(5)	-0.9(6)	-0.3(6)
Cl1	25.0(17)	12.2(15)	24.2(17)	-0.6(13)	3.6(14)	-0.1(13)
Cl4	24.4(18)	15.5(16)	24.8(17)	0.3(13)	2.1(14)	2.1(13)
Cl3	30.3(19)	28.3(18)	19.8(16)	3.9(14)	-3.5(14)	3.0(15)
Cl2	28.5(19)	17.1(17)	29.0(19)	-2.1(14)	7.4(15)	4.3(14)
C1	28(8)	25(7)	7(6)	-7(5)	-3(6)	-2(7)
C7	35(9)	12(7)	25(7)	-2(6)	-2(7)	-11(6)
C8	30.3(19)	28.3(18)	19.8(16)	3.9(14)	-3.5(14)	3.0(15)
C5	25(8)	25(8)	22(7)	-3(6)	-4(6)	1(6)
C2	23(7)	14(7)	19(7)	0(5)	-4(6)	-7(6)
C3	28(8)	14(7)	23(7)	-4(6)	-8(6)	5(6)
C12	22(7)	20(7)	26(8)	0(6)	-1(6)	-3(6)
C6	28(8)	19(7)	25(8)	-6(6)	9(6)	2(6)
C11	33(9)	18(7)	29(8)	-1(6)	17(7)	-3(6)
C4	30(8)	17(7)	23(7)	2(6)	-7(6)	3(6)
C10	25(8)	25(8)	30(8)	-18(7)	8(7)	-2(6)
C9	20(8)	52(11)	26(8)	2(8)	5(6)	-9(8)

Table S92. Bond Lengths for AgSePh-Cl_2(2,6) (2412930).

Atom	Atom	Length/Å	Atom	Atom	Length/Å
Ag1	Ag2[1]	3.1775(18)	Cl2	C6	1.719(15)
Ag1	Ag2[2]	3.0743(18)	C1	C2	1.42(2)
Ag1	Se1	2.674(2)	C1	C6	1.43(2)
Ag1	Se1[3]	2.681(2)	C7	C8	1.41(2)
Ag1	Se2[1]	2.6928(18)	C7	C12	1.38(2)
Ag2	Se1	2.6580(18)	C8	C9	1.38(2)
Ag2	Se2[3]	2.587(2)	C5	C6	1.39(2)
Ag2	Se2	2.587(2)	C5	C4	1.35(2)
Se1	C1	1.905(14)	C2	C3	1.38(2)
Se2	C7	1.935(14)	C3	C4	1.41(2)
Cl1	C2	1.751(15)	C12	C11	1.39(2)
Cl4	C12	1.753(16)	C11	C10	1.34(2)
Cl3	C8	1.759(16)	C10	C9	1.43(2)

[1]1-X,1-Y,1-Z; [2]2-X,1-Y,1-Z; [3]1+X,+Y,+Z

Table S93. Bond Angles for AgSePh-Cl_2(2,6) (2412930).

Atom	Atom	Atom	Angle/°	Atom	Atom	Atom	Angle/°
Ag2[1]	Ag1	Ag2[2]	96.08(4)	Ag2	Se2	Ag2[4]	127.94(7)
Se1[3]	Ag1	Ag2[2]	158.46(5)	C7	Se2	Ag1[2]	105.9(4)
Se1	Ag1	Ag2[1]	157.28(6)	C7	Se2	Ag2	109.2(5)
Se1	Ag1	Ag2[2]	67.82(5)	C7	Se2	Ag2[4]	116.8(5)
Se1[3]	Ag1	Ag2[1]	69.37(5)	C2	C1	Se1	123.7(10)
Se1	Ag1	Se1[3]	120.52(6)	C2	C1	C6	114.2(13)
Se1	Ag1	Se2[2]	117.93(6)	C6	C1	Se1	122.0(11)
Se1[3]	Ag1	Se2[2]	120.79(7)	C8	C7	Se2	118.2(12)
Se2[2]	Ag1	Ag2[2]	51.49(4)	C12	C7	Se2	124.6(11)
Se2[2]	Ag1	Ag2[1]	52.81(5)	C12	C7	C8	117.0(13)
Ag1[1]	Ag2	Ag1[2]	96.08(4)	C7	C8	Cl3	121.6(12)
Se1	Ag2	Ag1[2]	89.53(5)	C9	C8	Cl3	115.6(12)
Se1	Ag2	Ag1[1]	84.81(5)	C9	C8	C7	122.8(15)
Se2	Ag2	Ag1[1]	139.40(6)	C4	C5	C6	121.3(15)
Se2[3]	Ag2	Ag1[2]	140.71(6)	C1	C2	Cl1	120.4(11)
Se2[3]	Ag2	Ag1[1]	56.01(5)	C3	C2	Cl1	115.7(11)
Se2	Ag2	Ag1[2]	54.54(5)	C3	C2	C1	123.8(14)
Se2	Ag2	Se1	118.12(7)	C2	C3	C4	118.6(13)
Se2[3]	Ag2	Se1	111.96(6)	C7	C12	Cl4	120.9(11)
Se2	Ag2	Se2[3]	127.94(7)	C7	C12	C11	120.7(14)
Ag1	Se1	Ag1[4]	120.52(6)	C11	C12	Cl4	118.1(12)
Ag2	Se1	Ag1[4]	104.30(6)	C1	C6	Cl2	119.9(12)
Ag2	Se1	Ag1	109.99(6)	C5	C6	Cl2	118.2(12)
C1	Se1	Ag1[4]	99.6(5)	C5	C6	C1	121.9(14)
C1	Se1	Ag1	109.2(5)	C10	C11	C12	122.0(15)
C1	Se1	Ag2	113.0(4)	C5	C4	C3	120.0(14)
Ag2	Se2	Ag1[2]	73.97(5)	C11	C10	C9	119.6(15)
Ag2[4]	Se2	Ag1[2]	71.18(5)	C8	C9	C10	117.8(15)

[1]2-X,1-Y,1-Z; [2]1-X,1-Y,1-Z; [3]1+X,+Y,+Z; [4]-1+X,+Y,+Z

Table S94. Torsion Angles for AgSePh-Cl_2(2,6) (2412930).

A	B	C	D	Angle/°	A	B	C	D	Angle/°
Se1	C1	C2	Cl1	6.0(18)	C8	C7	C12	Cl4	-176.9(12)
Se1	C1	C2	C3	-174.8(11)	C8	C7	C12	C11	-3(2)
Se1	C1	C6	Cl2	-5.8(18)	C2	C1	C6	Cl2	178.5(11)
Se1	C1	C6	C5	175.1(12)	C2	C1	C6	C5	-1(2)
Se2	C7	C8	Cl3	-4.9(19)	C2	C3	C4	C5	-5(2)
Se2	C7	C8	C9	175.6(13)	C12	C7	C8	Cl3	179.3(12)
Se2	C7	C12	Cl4	8(2)	C12	C7	C8	C9	0(2)
Se2	C7	C12	C11	-179.0(12)	C12	C11	C10	C9	-1(2)
Cl1	C2	C3	C4	-179.1(11)	C6	C1	C2	Cl1	-178.3(11)
Cl4	C12	C11	C10	178.0(12)	C6	C1	C2	C3	1(2)
Cl3	C8	C9	C10	-176.5(12)	C6	C5	C4	C3	5(2)
C1	C2	C3	C4	2(2)	C11	C10	C9	C8	-2(2)
C7	C8	C9	C10	3(2)	C4	C5	C6	Cl2	178.6(12)
C7	C12	C11	C10	4(2)	C4	C5	C6	C1	-2(2)

Table S95. Hydrogen Atom Coordinates ($Å\times10^4$) and Isotropic Displacement Parameters ($Å^2\times10^3$) for AgSePh-Cl_2(2,6) (2412930).

Atom	*x*	*y*	*z*	U(eq)
H5	1326.95	1233.75	6569.33	29
H3	6887.64	41.5	5486.51	26
H11	-1795.79	9348.47	6782.15	32
H4	3416.23	-161.19	6185.89	28
H10	1375.39	9449.01	7498.12	32
H9	4081.95	7986.81	7763.88	39

14.15 AgSePy (CCDC-2412932)

Table S96. Fractional Atomic Coordinates (×10^4) and Equivalent Isotropic Displacement Parameters (Å^2×10^3) for AgSePy (2412932). U_{eq} is defined as 1/3 of the trace of the orthogonalised U_{IJ} tensor.

Atom	*x*	*y*	*z*	U(eq)
Ag2	3823.2(3)	5781.2(2)	1493.0(2)	14.55(5)
Ag3	5433.6(3)	3600.2(2)	524.8(2)	14.62(5)
Ag1	7922.3(3)	6393.9(2)	1352.3(2)	14.79(5)
Se3	683.5(3)	3846.7(2)	430.4(2)	12.29(5)
Se1	4177.9(3)	7973.8(2)	1545.7(2)	13.20(5)
Se2	8236.4(3)	5172.9(2)	2621.7(2)	13.40(5)
N3	3023(3)	2323.9(19)	920.5(18)	14.9(4)
N1	8423(3)	8577.5(19)	2569.5(18)	15.1(4)
N2	5181(3)	6150.1(19)	3485.7(18)	16.1(4)
C11	1233(3)	2569(2)	881.3(19)	13.1(4)
C13	102(4)	979(2)	1473(2)	18.2(5)
C12	-271(4)	1914(2)	1156(2)	15.8(4)
C8	6654(4)	6460(2)	5833(2)	18.4(5)
C7	7655(4)	5999(2)	4987(2)	16.3(4)
C14	1949(4)	717(2)	1508(2)	18.2(5)
C6	6872(3)	5846(2)	3805(2)	13.6(4)
C15	3350(4)	1403(2)	1226(2)	18.1(4)
C1	6825(3)	9042(2)	2622(2)	13.7(4)
C9	4901(4)	6781(2)	5501(2)	19.8(5)
C10	4222(4)	6611(2)	4321(2)	18.8(5)
C3	8943(4)	11112(2)	4200(2)	20.0(5)
C2	7035(4)	10310(2)	3444(2)	17.9(4)
C5	10273(4)	9373(2)	3308(2)	17.0(4)
C4	10607(4)	10640(2)	4125(2)	19.0(5)

Table S97. Anisotropic Displacement Parameters ($Å^2 \times 10^3$) for AgSePy (2412932). The Anisotropic displacement factor exponent takes the form: $-2\pi^2[h^2a^{*2}U_{11}+2hka^*b^*U_{12}+\ldots]$.

Atom	U_{11}	U_{22}	U_{33}	U_{23}	U_{13}	U_{12}
Ag2	15.13(8)	12.81(8)	15.79(8)	7.54(6)	3.28(6)	3.72(6)
Ag3	15.63(8)	14.45(8)	13.25(8)	5.81(6)	4.44(6)	4.76(6)
Ag1	17.35(8)	15.26(8)	15.41(8)	8.89(7)	6.79(6)	6.76(6)
Se3	13.02(10)	12.37(10)	14.02(10)	7.86(8)	4.71(8)	4.54(8)
Se1	14.39(10)	11.11(10)	14.18(10)	5.52(8)	4.35(8)	4.91(8)
Se2	14.31(10)	14.28(11)	12.79(10)	7.11(8)	4.01(8)	5.01(8)
N3	16.7(9)	14.0(9)	14.4(9)	6.7(7)	4.8(7)	5.3(7)
N1	17.6(9)	14.1(9)	14.5(9)	7.2(7)	5.1(7)	5.0(7)
N2	16.4(9)	15.5(9)	15.4(9)	6.6(8)	4.6(7)	4.2(7)
C11	17.7(10)	10.1(9)	9.3(9)	3.6(8)	2.7(8)	3.2(8)
C13	24.4(12)	15.2(11)	14.3(10)	7.9(9)	5.0(9)	2.4(9)
C12	17.2(10)	16.0(11)	14.9(10)	8.2(9)	4.9(8)	4.0(8)
C8	26.9(12)	12.7(10)	11.1(10)	4.5(8)	3.7(9)	0.1(9)
C7	17.0(10)	13.8(10)	15.7(10)	7.2(9)	2.1(8)	1.8(8)
C14	27.1(12)	13.3(10)	14.6(10)	7.1(9)	4.8(9)	7.4(9)
C6	15.0(10)	12.2(10)	11.1(9)	4.6(8)	3.4(8)	1.3(8)
C15	23.2(11)	19.6(11)	14.4(10)	8.9(9)	5.6(9)	10.8(9)
C1	15.6(10)	13.6(10)	12.3(9)	6.5(8)	4.9(8)	3.4(8)
C9	25.0(12)	14.8(11)	17.0(11)	4.4(9)	9.9(9)	3.2(9)
C10	17.9(10)	17.0(11)	19.4(11)	6.5(9)	6.0(9)	5.4(9)
C3	23.6(12)	13.6(11)	19.1(11)	5.3(9)	7.6(9)	1.2(9)
C2	21.1(11)	13.2(10)	18.2(11)	6.0(9)	6.9(9)	4.5(9)
C5	15.3(10)	20.3(11)	17.6(10)	11.0(9)	5.6(8)	4.3(9)
C4	18.9(11)	19.3(11)	16.9(11)	10.0(9)	3.1(9)	-0.4(9)

Table S98. Bond Lengths for AgSePy (2412932).

Atom	Atom	Length/Å	Atom	Atom	Length/Å
Ag2	Ag3	2.9276(3)	N3	C11	1.340(3)
Ag2	Ag3[1]	3.0542(3)	N3	C15	1.351(3)
Ag2	Ag1[1]	3.2512(3)	N1	C1	1.342(3)
Ag2	Ag1	2.9240(3)	N1	C5	1.350(3)
Ag2	Se3	2.5595(3)	N2	C6	1.338(3)
Ag2	Se1	2.5724(3)	N2	C10	1.349(3)
Ag2	N2	2.322(2)	C11	C12	1.401(3)
Ag3	Ag1[1]	2.9874(2)	C13	C12	1.385(3)
Ag3	Ag1	3.1253(3)	C13	C14	1.389(4)
Ag3	Se1[1]	2.6221(3)	C8	C7	1.381(3)
Ag3	Se2	2.6308(3)	C8	C9	1.390(4)
Ag3	N3	2.367(2)	C7	C6	1.402(3)
Ag1	Se3[1]	2.5713(3)	C14	C15	1.378(4)
Ag1	Se2	2.5847(3)	C1	C2	1.402(3)
Ag1	N1	2.342(2)	C9	C10	1.384(4)
Se3	C11	1.914(2)	C3	C2	1.386(4)
Se1	C1	1.917(2)	C3	C4	1.391(4)
Se2	C6	1.918(2)	C5	C4	1.383(3)

[1]1-X,1-Y,-Z

Table S99. Bond Angles for AgSePy (2412932).

Atom	Atom	Atom	Angle/°
Ag3	Ag2	Ag3^1	88.630(7)
Ag3	Ag2	Ag1^1	57.542(6)
Ag3^1	Ag2	Ag1^1	59.328(6)
Ag1	Ag2	Ag3^1	59.914(6)
Ag1	Ag2	Ag3	64.566(6)
Ag1	Ag2	Ag1^1	92.390(7)
Se3	Ag2	Ag3	76.176(8)
Se3	Ag2	Ag3^1	105.164(8)
Se3	Ag2	Ag1	137.404(9)
Se3	Ag2	Ag1^1	50.841(7)
Se3	Ag2	Se1	127.901(10)
Se1	Ag2	Ag3	138.321(9)
Se1	Ag2	Ag3^1	54.745(7)
Se1	Ag2	Ag1	78.684(8)
Se1	Ag2	Ag1^1	107.864(8)
N2	Ag2	Ag3^1	145.27(5)
N2	Ag2	Ag3	91.79(5)
N2	Ag2	Ag1	89.15(5)
N2	Ag2	Ag1^1	144.45(5)
N2	Ag2	Se3	108.64(5)
N2	Ag2	Se1	107.26(5)
Ag2	Ag3	Ag2^1	91.369(7)
Ag2	Ag3	Ag1^1	66.677(6)
Ag2^1	Ag3	Ag1	63.474(6)
Ag2	Ag3	Ag1	57.660(6)
Ag1^1	Ag3	Ag2^1	57.878(6)
Ag1^1	Ag3	Ag1	93.747(7)
Se1^1	Ag3	Ag2	139.514(9)
Se1^1	Ag3	Ag2^1	53.236(7)
Se1^1	Ag3	Ag1	110.259(8)
Se1^1	Ag3	Ag1^1	76.772(8)
Se1^1	Ag3	Se2	129.369(10)
Se2	Ag3	Ag2^1	110.388(9)
Se2	Ag3	Ag2	77.099(8)
Se2	Ag3	Ag1^1	140.724(9)
Se2	Ag3	Ag1	52.510(7)
Se3^1	Ag1	Ag3^1	74.927(8)
Se3^1	Ag1	Se2	128.333(10)
Se2	Ag1	Ag2	77.866(8)
Se2	Ag1	Ag2^1	105.877(8)
Se2	Ag1	Ag3	53.865(7)
Se2	Ag1	Ag3^1	135.146(9)
N1	Ag1	Ag2	91.38(5)
N1	Ag1	Ag2^1	140.66(5)
N1	Ag1	Ag3^1	89.36(5)
N1	Ag1	Ag3	146.90(5)
N1	Ag1	Se3^1	107.55(5)
N1	Ag1	Se2	112.32(5)
Ag2	Se3	Ag1^1	78.640(9)
C11	Se3	Ag2	108.82(7)
C11	Se3	Ag1^1	107.56(7)
Ag2	Se1	Ag3^1	72.019(8)
C1	Se1	Ag2	106.92(7)
C1	Se1	Ag3^1	107.88(7)
Ag1	Se2	Ag3	73.625(8)
C6	Se2	Ag3	107.09(7)
C6	Se2	Ag1	105.94(7)
C11	N3	Ag3	117.31(15)
C11	N3	C15	117.9(2)
C15	N3	Ag3	124.72(16)
C1	N1	Ag1	119.75(15)
C1	N1	C5	118.3(2)
C5	N1	Ag1	121.65(16)
C6	N2	Ag2	120.39(15)
C6	N2	C10	119.0(2)
C10	N2	Ag2	120.53(16)
N3	C11	Se3	119.48(17)
N3	C11	C12	122.2(2)
C12	C11	Se3	118.29(17)
C12	C13	C14	119.1(2)
C13	C12	C11	118.8(2)
C7	C8	C9	119.3(2)

N3	Ag3	Ag2	91.81(5)	C8	C7	C6	119.4(2)
N3	Ag3	Ag2^1	141.88(5)	C15	C14	C13	118.5(2)
N3	Ag3	Ag1	144.60(5)	N2	C6	Se2	119.41(16)
N3	Ag3	Ag1^1	89.00(5)	N2	C6	C7	121.2(2)
N3	Ag3	Se1^1	104.73(5)	C7	C6	Se2	119.40(18)
N3	Ag3	Se2	107.35(5)	N3	C15	C14	123.4(2)
Ag2	Ag1	Ag2^1	87.610(7)	N1	C1	Se1	120.08(17)
Ag2	Ag1	Ag3^1	62.208(6)	N1	C1	C2	121.6(2)
Ag2	Ag1	Ag3	57.773(6)	C2	C1	Se1	118.34(17)
Ag3^1	Ag1	Ag2^1	55.781(6)	C10	C9	C8	118.1(2)
Ag3	Ag1	Ag2^1	57.197(6)	N2	C10	C9	123.0(2)
Ag3^1	Ag1	Ag3	86.252(7)	C2	C3	C4	119.2(2)
Se3^1	Ag1	Ag2^1	50.518(7)	C3	C2	C1	119.3(2)
Se3^1	Ag1	Ag2	132.846(9)	N1	C5	C4	123.5(2)
Se3^1	Ag1	Ag3	102.905(8)	C5	C4	C3	118.1(2)

^{1}1-X,1-Y,-Z

Table S100. Torsion Angles for 2412932.

A	B	C	D	Angle/°	A	B	C	D	Angle/°
Ag2	N2	C6	Se2	2.8(2)	C8	C7	C6	Se2	-178.79(18)
Ag2	N2	C6	C7	-176.61(16)	C8	C7	C6	N2	0.6(3)
Ag2	N2	C10	C9	176.22(19)	C8	C9	C10	N2	0.1(4)
Ag3	N3	C11	Se3	3.3(2)	C7	C8	C9	C10	0.5(4)
Ag3	N3	C11	C12	-176.97(16)	C14	C13	C12	C11	-0.1(3)
Ag3	N3	C15	C14	176.79(18)	C6	N2	C10	C9	-0.3(4)
Ag1	N1	C1	Se1	-8.2(2)	C15	N3	C11	Se3	-179.09(17)
Ag1	N1	C1	C2	172.52(17)	C15	N3	C11	C12	0.6(3)
Ag1	N1	C5	C4	-173.65(18)	C1	N1	C5	C4	0.6(3)
Se3	C11	C12	C13	179.44(17)	C9	C8	C7	C6	-0.8(4)
Se1	C1	C2	C3	-177.75(19)	C10	N2	C6	Se2	179.38(17)
N3	C11	C12	C13	-0.3(3)	C10	N2	C6	C7	-0.1(3)
N1	C1	C2	C3	1.5(4)	C2	C3	C4	C5	-1.3(4)
N1	C5	C4	C3	0.9(4)	C5	N1	C1	Se1	177.38(17)
C11	N3	C15	C14	-0.6(3)	C5	N1	C1	C2	-1.9(3)
C13	C14	C15	N3	0.3(4)	C4	C3	C2	C1	0.1(4)
C12	C13	C14	C15	0.1(4)					

Table S100. Hydrogen Atom Coordinates (Å×10^4) and Isotropic Displacement Parameters (Å^2×10^3) for AgSePy (2412932).

**Atom**	***x***	***y***	***z***	**U(eq)**
H13	-893.76	522.94	1664.06	22
H12	-1525.35	2108.52	1124.81	19
H8	7157.37	6558.24	6633.22	22
H7	8865.16	5787.02	5204.03	20
H14	2241.03	78.71	1721.12	22
H15	4610.57	1219.17	1246.92	22
H9	4189.39	7106.21	6067.91	24
H10	3026.03	6829.61	4088.91	23
H3	9112.63	11972.46	4763.28	24
H2	5881.2	10615.35	3481.16	22
H5	11404.59	9045.53	3263.68	20
H4	11935.44	11173.47	4621.13	23

15. References

(1) Schrauzer, G.; Prakash, H. Synthesis and electrical properties of transition metal mercaptides of 1, 4-dimercaptobenzene. *Inorganic Chemistry* **1975**, *14* (5), 1200–1204.
(2) Dance, I. G.; Fitzpatrick, L. J.; Rae, A. D.; Scudder, M. L. The intertwined double-(-AgSR-). infin.-strand chain structure of crystalline (3-methylpentane-3-thiolato) silver, in relation to (AgSr) 8 molecules in solution. *Inorganic Chemistry* **1983**, *22* (25), 3785–3788.
(3) Chadha, R.; Kumar, R.; Tuck, D. G. The preparation and crystal structure of the unusual copper–sulphur cage complex Cu 8 (SC 5 H 11) 4 (S 2 CSC 5 H 11) 4. *Journal of the Chemical Society, Chemical Communications* **1986**, (3), 188–189.
(4) Tang, K.; Aslam, M.; Block, E.; Nicholson, T.; Zubieta, J. Steric control of aggregation in neutral silver (I) thiolates,[AgSR] n. Crystal and molecular structures of [AgSCH (SiMe3) 2] 8, a discrete molecular biscycle of weakly interacting [AgSCH (SiMe3) 2] 4 units, and of [AgSC (SiPhMe2) 3] 3 and [AgSC (SiMe3) 3] 4, discrete molecular monocycles containing linearly coordinated silver (I) and doubly bridging mercapto sulfur donors from novel sterically hindered thiolate ligands. A comparison with the nonmolecular structure of [Ag4 {SCH2 (SiMe3)} 3] n1. *Inorganic Chemistry* **1987**, *26* (10), 1488–1497.
(5) Chadra, R. K.; Kumar, R.; Tuck, D. G. The synthesis, properties and crystal structure of the copper (I)-thiolato-thioxanthato complex Cu8 (SC5H11) 4 (S2CSC5H11) 4. *Polyhedron* **1988**, *7* (12), 1121–1128.
(6) Kitagawa, S.; Munakata, M.; Shimono, H.; Matsuyama, S.; Masuda, H. Synthesis and crystal structure of hexanuclear copper (I) complexes of μ3-pyridine-2-thionate. *Journal of the Chemical Society, Dalton Transactions* **1990**, (7), 2105–2109.
(7) Dance, I. G.; Fisher, K. J.; Banda, R. H.; Scudder, M. L. Layered structure of crystalline compounds silver thiolates (AgSR). *Inorganic chemistry* **1991**, *30* (2), 183–187.
(8) Baumgartner, M.; Schmalle, H.; Baerlocher, C. Synthesis, Characterization, and Crystal Structure of Three Homoleptic Copper (I) Thiolates:(Cu (CH3S-))∞,[(C6H5) 4P+] 2 [Cu5 (CH3S-) 7]· C2H6O2, and [(C3H7) 4N+] 2 [Cu4 (CH3S-) 6· CH4O. *Journal of Solid State Chemistry* **1993**, *107* (1), 63–75.
(9) Fijolek, H. G.; Grohal, J. R.; Sample, J. L.; Natan, M. J. A facile trans to gauche conversion in layered silver butanethiolate. *Inorganic Chemistry* **1997**, *36* (4), 622–628.
(10) Espinet, P.; Lequerica, M. C.; Martín-Alvarez, J. M. Synthesis, structural characterization and mesogenic behavior of copper (I) n-alkylthiolates. *Chemistry–A European Journal* **1999**, *5* (7), 1982–1986.
(11) Parikh, A.; Gillmor, S.; Beers, J.; Beardmore, K.; Cutts, R.; Swanson, B. Characterization of chain molecular assemblies in long-chain, layered silver thiolates: a joint infrared spectroscopy and X-ray diffraction study. *The Journal of Physical Chemistry B* **1999**, *103* (15), 2850–2861.
(12) Su, W.; Hong, M.; Weng, J.; Liang, Y.; Zhao, Y.; Cao, R.; Zhou, Z.; Chan, A. S. Tunable polymerization of silver complexes with organosulfur ligand: counterions effect, solvent-and temperature-dependence in the formation of silver (I)-thiolate (and/or thione) complexes. *Inorganica chimica acta* **2002**, *331* (1), 8–15.
(13) Cuthbert, H. L.; Wallbank, A. I.; Taylor, N. J.; Corrigan, J. F. Synthesis and Structural Characterization of [Cu20Se4 (μ3-SePh) 12 (PPh3) 6] and [Ag (SePh)]∞. *Zeitschrift für anorganische und allgemeine Chemie* **2002**, *628* (11), 2483–2488.

(14) Che, C. M.; Li, C. H.; Chui, S. S. Y.; Roy, V. e. A. e. L.; Low, K. H. Homoleptic Copper (I) Arylthiolates as a New Class of p-Type Charge Carriers: Structures and Charge Mobility Studies. *Chemistry–A European Journal* **2008**, *14* (10), 2965–2975.
(15) Low, K.-H.; Roy, V.; Chui, S. S.-Y.; Chan, S. L.-F.; Che, C.-M. Highly conducting two-dimensional copper (I) 4-hydroxythiophenolate network. *Chemical Communications* **2010**, *46* (39), 7328–7330.
(16) Luo, Z.; Yuan, X.; Yu, Y.; Zhang, Q.; Leong, D. T.; Lee, J. Y.; Xie, J. From aggregation-induced emission of Au (I)–thiolate complexes to ultrabright Au (0)@ Au (I)–thiolate core–shell nanoclusters. *Journal of the American Chemical Society* **2012**, *134* (40), 16662–16670.
(17) Lavenn, C.; Okhrimenko, L.; Guillou, N.; Monge, M.; Ledoux, G.; Dujardin, C.; Chiriac, R.; Fateeva, A.; Demessence, A. A luminescent double helical gold (i)–thiophenolate coordination polymer obtained by hydrothermal synthesis or by thermal solid-state amorphous-to-crystalline isomerization. *Journal of Materials Chemistry C* **2015**, *3* (16), 4115–4125.
(18) Kole, G. K.; Vivekananda, K.; Kumar, M.; Ganguly, R.; Dey, S.; Jain, V. K. Hemilabile silver (I) complexes containing pyridyl chalcogenolate (S, Se) ligands and their utility as molecular precursors for silver chalcogenides. *CrystEngComm* **2015**, *17* (23), 4367–4376.
(19) Lavenn, C.; Guillou, N.; Monge, M.; Podbevšek, D.; Ledoux, G.; Fateeva, A.; Demessence, A. Shedding light on an ultra-bright photoluminescent lamellar gold thiolate coordination polymer [Au (p-SPhCO 2 Me)] n. *Chemical Communications* **2016**, *52* (58), 9063–9066.
(20) Veselska, O.; Demessence, A. d10 coinage metal organic chalcogenolates: From oligomers to coordination polymers. *Coordination Chemistry Reviews* **2018**, *355*, 240–270.
(21) Yeung, M.; Popple, D. C.; Schriber, E. A.; Teat, S. J.; Beavers, C. M.; Demessence, A.; Kuykendall, T. R.; Hohman, J. N. Corrosion of Late-and Post-Transition Metals into Metal–Organic Chalcogenolates and Implications for Nanodevice Architectures. *ACS Applied Nano Materials* **2020**, *3* (4), 3568–3577.
(22) Vaidya, S.; Veselska, O.; Zhadan, A.; Diaz-Lopez, M.; Joly, Y.; Bordet, P.; Guillou, N.; Dujardin, C.; Ledoux, G.; Toche, F. Transparent and luminescent glasses of gold thiolate coordination polymers. *Chemical Science* **2020**, *11* (26), 6815–6823.
(23) Aleksich, M.; Paley, D. W.; Schriber, E. A.; Linthicum, W.; Oklejas, V.; Mittan-Moreau, D. W.; Kelly, R. P.; Kotei, P. A.; Ghodsi, A.; Sierra, R. G. XFEL microcrystallography of self-assembling silver n-alkanethiolates. *Journal of the American Chemical Society* **2023**, *145* (31), 17042–17055.
(24) Sakurada, T.; Cho, Y.; Paritmongkol, W.; Lee, W. S.; Wan, R.; Su, A.; Shcherbakov-Wu, W.; Müller, P.; Kulik, H. J.; Tisdale, W. A. 1D Hybrid Semiconductor Silver 2, 6-Difluorophenylselenolate. *Journal of the American Chemical Society* **2023**, *145* (9), 5183–5190.
(25) Khamlue, R.; Sakurada, T.; Cho, Y.; Lee, W. S.; Leangtanom, P.; Taylor, M. G.; Naewthong, W.; Sripetch, P.; Na Ranong, B.; Autila, T. Heterocyclic Modification Leading to Luminescent 0D Metal Organochalcogenide with Stable X-ray Scintillating Properties. *Chemistry of Materials* **2024**, *36* (10), 5238–5249.
(26) Fan, Q.; Willson, M. C.; Foell, K. A.; Paley, D. W.; Kotei, P. A.; Schriber, E. A.; Rosenberg, D. J.; Rani, K.; Tchoń, D. M.; Zeller, M. Nucleophilic Displacement Reactions of Silver-Based Metal–Organic Chalcogenolates. *Journal of the American Chemical Society* **2024**, *146* (44), 30349–30360.
(27) Ye, Z.; Zhao, J.; Kang, K.; McCormack, S. J.; Shao, Y. T.; Efremov, M. Y.; Schleife, A.; Kriven, W. M.; Zuo, J. M.; Allen, L. H. Birefringent Color Filter by Layered Metal-Organic

Chalcogenides: In-Plane Anisotropy and Odd/Even Effect. *Advanced Optical Materials* **2024**, 2402159.
(28) Lee, W. S.; Müller, P.; Samulewicz, N.; Deshpande, T.; Wan, R.; Tisdale, W. A. Synthesis and Structural Anisotropy of Single-Crystalline 2D AgEPh (E= S, Se, Te). *Chemistry of Materials* **2024**, *36* (19), 9904–9913.
(29) Schriber, E. A.; Paley, D. W.; Bolotovsky, R.; Rosenberg, D. J.; Sierra, R. G.; Aquila, A.; Mendez, D.; Poitevin, F.; Blaschke, J. P.; Bhowmick, A. Chemical crystallography by serial femtosecond X-ray diffraction. *Nature* **2022**, *601* (7893), 360–365.
(30) Paritmongkol, W.; Sakurada, T.; Lee, W. S.; Wan, R.; Muller, P.; Tisdale, W. A. Size and quality enhancement of 2D semiconducting metal–organic chalcogenolates by amine addition. *Journal of the American Chemical Society* **2021**, *143* (48), 20256–20263.
(31) Li, Y.; Jiang, X.; Fu, Z.; Huang, Q.; Wang, G.-E.; Deng, W.-H.; Wang, C.; Li, Z.; Yin, W.; Chen, B. Coordination assembly of 2D ordered organic metal chalcogenides with widely tunable electronic band gaps. *Nature Communications* **2020**, *11* (1), 261.
(32) Kotei, P. A.; Paley, D. W.; Oklejas, V.; Mittan-Moreau, D. W.; Schriber, E. A.; Aleksich, M.; Willson, M. C.; Inoue, I.; Owada, S.; Tono, K. Engineering Supramolecular Hybrid Architectures with Directional Organofluorine Bonds. *Small science* **2024**, *4* (1), 2300110.
(33) Hong, M.; Su, W.; Cao, R.; Zhang, W.; Lu, J. Controlled assembly based on multibridging thiolate ligands: new polymeric silver (I) complexes with one-dimensional chain and three-dimensional network structures. *Inorganic chemistry* **1999**, *38* (3), 600–602.
(34) Maserati, L.; Pecorario, S.; Prato, M.; Caironi, M. Understanding the synthetic pathway to large-area, high-quality [AgSePh]∞ nanocrystal films. *The Journal of Physical Chemistry C* **2020**, *124* (41), 22845–22852.
(35) Mendes, S. R.; Amado, A. M.; Tomkinson, J.; Marques, M. P. M.; de Carvalho, L. A. B. Vibrational and conformational studies of 1, 3-diaminopropane and its N-deuterated and N-ionised derivatives. *New Journal of Chemistry* **2017**, *41* (18), 10132–10147.
